# Comparative proteomic analyses of Duchenne muscular dystrophy and Becker muscular dystrophy muscles: changes contributing to preserve muscle function in Becker muscular dystrophy patients

**DOI:** 10.1002/jcsm.12527

**Published:** 2020-01-28

**Authors:** Daniele Capitanio, Manuela Moriggi, Enrica Torretta, Pietro Barbacini, Sara De Palma, Agnese Viganò, Hanns Lochmüller, Francesco Muntoni, Alessandra Ferlini, Marina Mora, Cecilia Gelfi

**Affiliations:** ^1^ Department of Biomedical Sciences for Health University of Milan Milan Italy; ^2^ IRCCS Istituto Ortopedico Galeazzi Milan Italy; ^3^ Department of Neuropediatrics and Muscle Disorders, Faculty of Medicine Medical Center—University of Freiburg Freiburg Germany; ^4^ Centro Nacional de Análisis Genómico (CNAG‐CRG), Center for Genomic Regulation Barcelona Institute of Science and Technology (BIST) Barcelona Catalonia Spain; ^5^ Children's Hospital of Eastern Ontario Research Institute University of Ottawa Ottawa Canada; ^6^ Division of Neurology, Department of Medicine The Ottawa Hospital Ottawa Canada; ^7^ Dubowitz Neuromuscular Centre University College London, Institute of Child Health London UK; ^8^ NIHR Great Ormond Street Hospital Biomedical Research Centre, Great Ormond Street Institute of Child Health, University College London, & Great Ormond Street Hospital Trust London UK; ^9^ Unit of Medical Genetics, Department of Medical Sciences University of Ferrara Ferrara Italy; ^10^ Neuromuscular Diseases and Neuroimmunology Unit Fondazione IRCCS Istituto Neurologico Carlo Besta Milan Italy

**Keywords:** Proteomics, Mechanotransduction, Bioenergetics, Duchenne muscular dystrophy, Becker muscular dystrophy, Reactive oxygen species

## Abstract

**Background:**

Duchenne muscular dystrophy (DMD) and Becker muscular dystrophy (BMD) are characterized by muscle wasting leading to loss of ambulation in the first or third decade, respectively. In DMD, the lack of dystrophin hampers connections between intracellular cytoskeleton and cell membrane leading to repeated cycles of necrosis and regeneration associated with inflammation and loss of muscle ordered structure. BMD has a similar muscle phenotype but milder. Here, we address the question whether proteins at variance in BMD compared with DMD contribute to the milder phenotype in BMD, thus identifying a specific signature to be targeted for DMD treatment.

**Methods:**

Proteins extracted from skeletal muscle from DMD/BMD patients and young healthy subjects were either reduced and solubilized prior two‐dimensional difference in gel electrophoresis/mass spectrometry differential analysis or tryptic digested prior label‐free liquid chromatography with tandem mass spectrometry. Statistical analyses of proteins and peptides were performed by DeCyder and Perseus software and protein validation and verification by immunoblotting.

**Results:**

Proteomic results indicate minor changes in the extracellular matrix (ECM) protein composition in BMD muscles with retention of mechanotransduction signalling, reduced changes in cytoskeletal and contractile proteins. Conversely, in DMD patients, increased levels of several ECM cytoskeletal and contractile proteins were observed whereas some proteins of fast fibres and of *Z*‐disc decreased. Detyrosinated alpha‐tubulin was unchanged in BMD and increased in DMD although neuronal nitric oxide synthase was unchanged in BMD and greatly reduced in DMD. Metabolically, the tissue is characterized by a decrement of anaerobic metabolism both in DMD and BMD compared with controls, with increased levels of the glycogen metabolic pathway in BMD. Oxidative metabolism is severely compromised in DMD with impairment of malate shuttle; conversely, it is active in BMD supporting the tricarboxylic acid cycle and respiratory chain. Adipogenesis characterizes DMD, whereas proteins involved in fatty acids beta‐oxidation are increased in BMD. Proteins involved in protein/amino acid metabolism, cell development, calcium handling, endoplasmic reticulum/sarcoplasmic reticulum stress response, and inflammation/immune response were increased in DMD. Both disorders are characterized by the impairment of *N*‐linked protein glycosylation in the endoplasmic reticulum. Authophagy was decreased in DMD whereas it was retained in BMD.

**Conclusions:**

The mechanosensing and metabolic disruption are central nodes of DMD/BMD phenotypes. The ECM proteome composition and the metabolic rewiring in BMD lead to preservation of energy levels supporting autophagy and cell renewal, thus promoting the retention of muscle function. Conversely, DMD patients are characterized by extracellular and cytoskeletal protein dysregulation and by metabolic restriction at the level of α‐ketoglutarate leading to shortage of glutamate‐derived molecules that over time triggers lipogenesis and lipotoxicity.

## Introduction

Mutations in the dystrophin gene cause Duchenne muscular dystrophy (DMD) and Becker muscular dystrophy (BMD),^1^, ^2^ characterized by a progressive skeletal muscle degeneration, weakness, loss of ambulation, and early death due to cardiorespiratory insufficiency.^3^ The absence of dystrophin causes DMD, whereas mutations resulting in a reduced amount or shortened dystrophin protein cause BMD. BMD has a mean onset at 12 years, and loss of ambulation occurs around the third decade of life, with a life expectancy that is not very dissimilar from the general population, in marked contrast to DMD patients, in whom mean life expectancy is in the third decade of life.^4^, ^5^, ^6^, ^7^ In DMD patients, signs and symptoms appear around 2 to 5 years of age.^8^, ^9^, ^10^ Over time, muscle dystrophic changes overwhelm muscle growth, causing progressive difficulty in walking and patients lose ambulation.^6^, ^11^, ^12^, ^13^, ^14^, ^15^

Muscles of DMD patients are characterized by variable degrees of atrophy, hypertrophy, muscle necrosis, regeneration, and fibrosis,^16^, ^17^ and the severity of muscle damage depends on age and muscle type. The primary event is the loss of the muscle system ordered structure due to lack of dystrophin that hampers the connection between the intracellular cytoskeleton and the cell membrane, reduced structural integrity of the sarcolemma leading to repeated cycles of necrosis and regeneration associated with inflammation and repair.^16^, ^17^, ^18^, ^19^, ^20^ As disease progresses, myofibres are replaced by fibro‐fatty tissue.^16^, ^21^

In BMD, skeletal muscle pathology is similar but milder.^22^, ^23^ In younger patients (15 years old), the muscle shows signs of muscle necrosis and regeneration, whereas in older patients (>15 years old), centralized nuclei, split fibres, hypertrophic muscle fibres, and fibrosis are observed.^22^, ^23^, ^24^

Recent studies indicate a role of neuronal nitric oxide synthase (nNOS), nitric oxide, reactive oxygen species (ROS), and reactive nitrogen species in the pathology of DMD and BMD.^18^ The nNOS is a signal transduction protein associated with the dystrophin complex producing nitric oxide, which regulates local vascular perfusion, calcium mobilization, glucose metabolism, and contractile function.^8^, ^12^, ^18^ In DMD patients, reduced nNOS activity results in ischaemia and Ca++ overload. Except for deletions of the dystrophin gene that remove the repeats 16–17 of the rod domain, which cause the loss of nNOS although maintaining a substantially functional protein,^25^ the presence of nNOS tethering at the sarcolemma level is generally retained in BMD. In DMD, the loss of nNOS is also associated with an increase in ROS and increased muscle protein carbonylation and lipid peroxidation.^18^, ^26^, ^27^, ^28^, ^29^ The combination of Ca++ overload and ROS signalling produces fibres overstretch contributing to muscle damage.^30^ Furthermore, accumulation of dysfunctional organelles, increased NADPH oxidase 2 activity, and the activation of Src kinases contribute to impaired autophagy in dystrophic muscle.^31^, ^32^, ^33^

Previous proteomic studies of muscle tissue of patients affected by Ullrich and Bethlem myopathies and in Col6a1−/− mice described an association between impaired autophagy and altered mechanotransduction induced by collagen VI deficiency in patients^34^, ^35^, ^36^, ^37^ and highlighted the role of metabolic rewiring in muscle wasting. In DMD patients, studies were mainly concentrated on search of biomarkers in biological fluids, where a body of literature exists.^38^, ^39^, ^40^, ^41^, ^42^, ^43^, ^44^, ^45^, ^46^ Furthermore, several proteomic analyses have been conducted in skeletal muscle and serum of dystrophin‐deficient mouse, dog, and pig models^47^, ^48^, ^49^, ^50^, ^51^, ^52^, ^53^; nevertheless, studies on the differential proteome composition of skeletal muscle of DMD and BMD patients are lacking, and there is a need of an omnicomprehensive view of proteins differentially expressed in DMD compared with BMD patients to build a map of pathways involved in the different patient outcome.

This study is focused on the identification of proteins differentially expressed between DMD and BMD muscles to find out key elements associated with the milder phenotype characterizing BMD, by means of two‐dimensional difference in gel electrophoresis (2D‐DIGE) and label‐free liquid chromatography with tandem mass spectrometry (LC‐MS/MS) proteomics. The study indicates a clear relationship between changes in structural and contractile proteins causing a disruption of the muscle organization in which post‐translational modification of detyrosinated tubulin plays a central role in triggering muscle wasting. Another important element introduced by this study is the conjunction of metabolic rewiring induced by altered mechanotransduction and ischaemia associated with the lack of nNOS tethering in DMD that is retained in BMD patients.

## Materials and methods

### Ethical statement

The protocol was approved by the University College London, the University of Newcastle, and the University of Ferrara (Local Ethical Committee approval no. 95‐2013) research ethics committees, and written informed consent was obtained. The investigation was conducted according to the Declaration of Helsinki.

### Patients

Skeletal muscle biopsies from 15 DMD and 15 BMD patients were collected by needle biopsy from vastus lateralis in the mid‐thigh, frozen in pre‐chilled isopentane, and stored in liquid nitrogen. Magnetic resonance imaging was adopted to minimize blood and fat contamination. Patients were diagnosed by genetic, biochemical, and immunohistochemical analysis (Supporting Information, *Table* S1). Biopsies from 15 young male healthy subjects were taken after an overnight fast, and in absence of strenuous exercise, samples were frozen in liquid nitrogen.

### Proteomic analysis

#### Protein extraction

Muscle biopsies were ground in a frozen mortar, suspended in lysis buffer [7 M urea, 2 M thiourea, 4% CHAPS, 30 mM Tris, 1 mM PMSF, 1% phosphatase inhibitor cocktail 1 and 2 (Sigma), pH 8.5], and solubilized by sonication on ice. Proteins were selectively precipitated using PlusOne 2D‐Clean up kit (GE Healthcare) in order to remove non‐protein impurities and resuspended in lysis buffer. Protein extracts were adjusted to pH 8.5 by addition of 1 M NaOH. Protein concentrations were determined by PlusOne 2D‐Quant kit (GE Healthcare).

#### Two‐dimensional difference in gel electrophoresis

Protein labelling, 2D separation, and analysis were performed exactly as previously described.^54^ Protein minimal labelling with cyanine dyes (Cy3 and Cy5) was performed, according to manufacturer's recommendations, by mixing 50 μg of each sample extract with 400 pmol CyDye (GE Healthcare) and incubating, on ice, in the dark for 30 min. The labelling reaction was quenched with 1 mL l‐lysine 10 mM on ice for 10 min in the dark. Sample proteins were labelled with Cy5 whereas the internal standard, generated by pooling individual samples (DMD, BMD, and control), was Cy3 labelled. Samples from each subject (40 μg) were combined with an equal amount of internal standard. Each sample was run in triplicate on 24 cm, 3–10 non‐linear pH‐gradient IPG strips, with a voltage gradient ranging from 200 to 8000 V, for a total of 75 000 Vh, using an IPGphor electrophoresis unit (GE Healthcare). After focusing, proteins were reduced and alkylated. The second dimension was carried out in 20 × 25 cm^2^, 12% T, 2.5% C constant concentration polyacrylamide gels at 20°C, and 15 mA per gel using the Ettan Dalt II system (GE Healthcare). CyDye‐labelled gels were visualized and acquired using a Typhoon 9200 Imager (GE Healthcare). Image analysis was performed using the DeCyder version 6.5 software (GE Healthcare). The proteomic profiles of vastus lateralis muscle of 15 DMD and 15 BMD patients were compared with 15 healthy control subjects. For each experimental group, spots present in at least 80% of samples were considered. Statistically significant differences of 2D‐DIGE data were computed by analysis of variance (ANOVA) and Tukey's tests (*P* < 0.01). False discovery rate was applied as a multiple test correction in order to keep the overall error rate as low as possible. In case the ANOVA test was not applicable, the non‐parametric Kruskal–Wallis test was used. Power analysis was conducted on statistically changed spots, and only spots that reached a sensitivity threshold > 0.8 were considered as differentially expressed. Protein identification was carried out by matrix‐assisted laser desorption/ionization–time‐of‐flight (MALDI‐ToF) mass spectrometry (MS). For protein identification, semi‐preparative gels were loaded with unlabelled sample (400 μg per strip); electrophoretic conditions were the same as 2D‐DIGE, and gels were stained with a total‐protein fluorescent stain (Krypton, Thermo Fisher Scientific). Image acquisition was performed using a Typhoon 9200 laser scanner. Spots of interest were excised from gel using the Ettan spot picker robotic system (GE Healthcare), destained in 50% methanol/50 mM ammonium bicarbonate, and incubated with 30 μL of 6 ng/mL trypsin (Promega) dissolved in 10 mM ammonium bicarbonate for 16 h at 37°C. Released peptides were subjected to reverse phase chromatography (Zip‐Tip C18 micro, Millipore), eluted with 50% acetonitrile (ACN)/0.1% trifluoroacetic acid. Peptides mixture (1 μL) was diluted in an equal volume of 10 mg/mL alpha‐cyano‐4‐hydroxycinnamic acid matrix dissolved in 70% ACN/30% citric acid and processed on an Ultraflex III MALDI‐ToF/ToF (Bruker Daltonics) mass spectrometer. MS was performed at an accelerating voltage of 20 kV, and spectra were externally calibrated using Peptide Mix calibration mixture (Bruker Daltonics); 1000 laser shots were taken per spectrum. Spectra were processed by FlexAnalysis software v. 3.0 (Bruker Daltonics) setting the signal to noise threshold value to 6, and search was carried out by correlation of uninterpreted spectra to *Homo sapiens* entries (327411sequences) in NCBIprot 20180429 (152462470 sequences; 55858910152 residues) using BioTools v. 3.2 (Bruker Daltonics) interfaced to the on‐line MASCOT software, which utilizes a robust probabilistic scoring algorithm. The significance threshold was set at *P*‐value < 0.05. No mass and pI constraints were applied, and trypsin was set as enzyme. One missed cleavage per peptide was allowed, and carbamidomethylation was set as fixed modification while methionine oxidation as variable modification. Mass tolerance was set at 30 ppm for MS spectra.

#### Label‐free liquid chromatography with tandem mass spectrometry

Proteins were precipitated with PlusOne 2D‐Clean up kit (GE Healthcare), resuspended in 50 mM ammonium bicarbonate and 0.1% RapiGest SF surfactant (Waters), reduced, carbamydomethylated, and digested with sequence grade trypsin (Promega) for 16 h at 37°C using a protein:trypsin ratio of 50:1. Nano LC‐ESI‐MS/MS analysis was performed on a Dionex UltiMate 3000 HPLC System with a PicoFrit ProteoPrep C18 column (200 mm, internal diameter of 75 μm) (New Objective, USA) Gradient: 2% ACN in 0.1% formic acid for 15 min, 2–35% ACN in 0.1% formic acid for 240 min, 35–60% ACN in 0.1% formic acid for 60 min, and 60–100% ACN in 0.1% formic acid for 3 min at a flow rate of 0.3 μL/min. The eluate was electrosprayed into an LTQ Orbitrap Velos (Thermo Fisher Scientific) through a Proxeon nanoelectrospray ion source (Thermo Fisher Scientific). Three technical replicates were performed for each sample. The LTQ Orbitrap was operated in positive mode in data‐dependent acquisition mode to automatically alternate between a full scan (m/z 350–2000) in the Orbitrap (at resolution 60 000, automatic gain control target 1 000 000) and subsequent collision‐induced dissociation MS/MS in the linear ion trap of the 20 most intense peaks from full scan (normalized collision energy of 35%, 10 ms activation). Isolation window: 3 Da, unassigned charge states: rejected, charge state 1: rejected, charge states 2+, 3+, 4+: not rejected; dynamic exclusion enabled (60 s, exclusion list size: 200). Data acquisition was controlled by Xcalibur 2.0 and Tune 2.4 software (Thermo Fisher Scientific). Mass spectra were analysed using MaxQuant software (version 1.3.0.5).^55^ The initial maximum allowed mass deviation was set to 6 ppm for monoisotopic precursor ions and 0.5 Da for MS/MS peaks. Enzyme specificity was set to trypsin, defined as *C*‐terminal to arginine and lysine excluding proline, and a maximum of two missed cleavages was allowed. Carbamidomethylcysteine was set as a fixed modification, *N*‐terminal acetylation, and methionine oxidation as variable modifications. The spectra were searched by the Andromeda search engine against the human UniProt sequence database (release 2016_11). Protein identification required at least one unique or razor peptide per protein group. Quantification in MaxQuant was performed using the built‐in extracted ion chromatogram‐based label‐free quantification algorithm^56^ using fast label‐free quantification. The required false positive rate was set to 1% at the peptide and 1% at the protein level, and the minimum required peptide length was set to six amino acids. Statistical analyses were performed using the Perseus software (version 1.4.0.6).^57^ Only proteins present and quantified in at least two out of three technical repeats were considered as positively identified in a sample. For each experimental group, the proteins identified in at least 80% of samples were considered. Statistically significant differences were computed by ANOVA and false discovery rate (*P* < 0.05) followed by Tukey post hoc test (*P* < 0.01).

### Immunoblotting

Protein extracts (50 μg) from pooled DMD, BMD, and healthy control muscles were loaded in triplicate and resolved on 6%, 10%, and 12% polyacrylamide gels, according to protein molecular weight. Blots were incubated with rabbit or goat polyclonal primary antibodies (Santa Cruz Biotechnology, except where otherwise indicated) as follows: anti‐detyrosinated alpha‐tubulin (Abcam, dilution 1:500), anti‐nNOS (1:500), anti‐PHD3 (Novus, 1:500), anti‐CS (1:1000), anti‐FASN (1:500), anti‐PPARα (1:1000), anti‐GLUL (1:1000), anti‐FBP1 (Novus, 1:1000), anti‐STT3B (Proteintech, 1:1000), anti‐LC3BI/II (Cell Signaling Technology, 1:500), and anti‐BNIP3 (1:500). After washing, membranes were incubated with anti‐rabbit (GE Healthcare) or anti‐goat (Santa Cruz Biotechnology) secondary antibodies conjugated with horseradish peroxidase. Signals were visualized by chemiluminescence using the ECL Prime detection kit and the Image Quant LAS 4000 (GE Healthcare) analysis system. Band quantification was performed using the Image Quant TL (Molecular Dynamics) software followed by statistical analysis (ANOVA + Tukey, *n* = 3 or *n* = 2, *P* < 0.05) (for full length blot images, see Supporting Information, *Figure* S2). Band intensities were normalized against the total amount of proteins stained by Sypro ruby total‐protein blot stain (Thermo Fisher Scientific).

To confirm proteomic data, the following blots were performed as previously described: anti‐ACO2, anti‐OGDH, anti‐SDHA, anti‐CKM, anti‐LDHA, anti‐CRYAB, and anti‐VIM (Monosan) (dilution: 1:1000).

### Immunofluorescence imaging

For immunofluorescence, 6 μm thick cryosections from muscle biopsies of DMD and BMD patients and controls were incubated in one of the following primary monoclonal antibodies to dystrophin dys‐1 and dys‐2 (both diluted 1:50), β‐sarcoglycan (1:10, Novocastra, Leica Biosystems, Wetzlar, Germany); α‐dystroglycan clone IIH6 (1:1, Upstate Biotechnology Inc., Lake Placid, NY, USA); α‐dystrobrevin (1:50), α‐syntrophin (1:10), and sarcospan (1:10) (all from Santa Cruz Biotechnology, Santa Cruz, CA, USA); followed by incubation in biotinylated anti‐mouse IgG or IgM as appropriate (1:250, Jackson ImmunoResearch, Westgrove, PA, USA) and in Avidin, NeutrAvidin™, and Rhodamine Red™‐X‐conjugate (1:250, Molecular Probes, Thermo Fisher Scientific Inc., Rockford, IL, USA). Muscle sections were examined under a Zeiss Axioplan fluorescence microscope (Carl Zeiss AG, Oberkochen, Germany).

## Results

**Figure 1:**
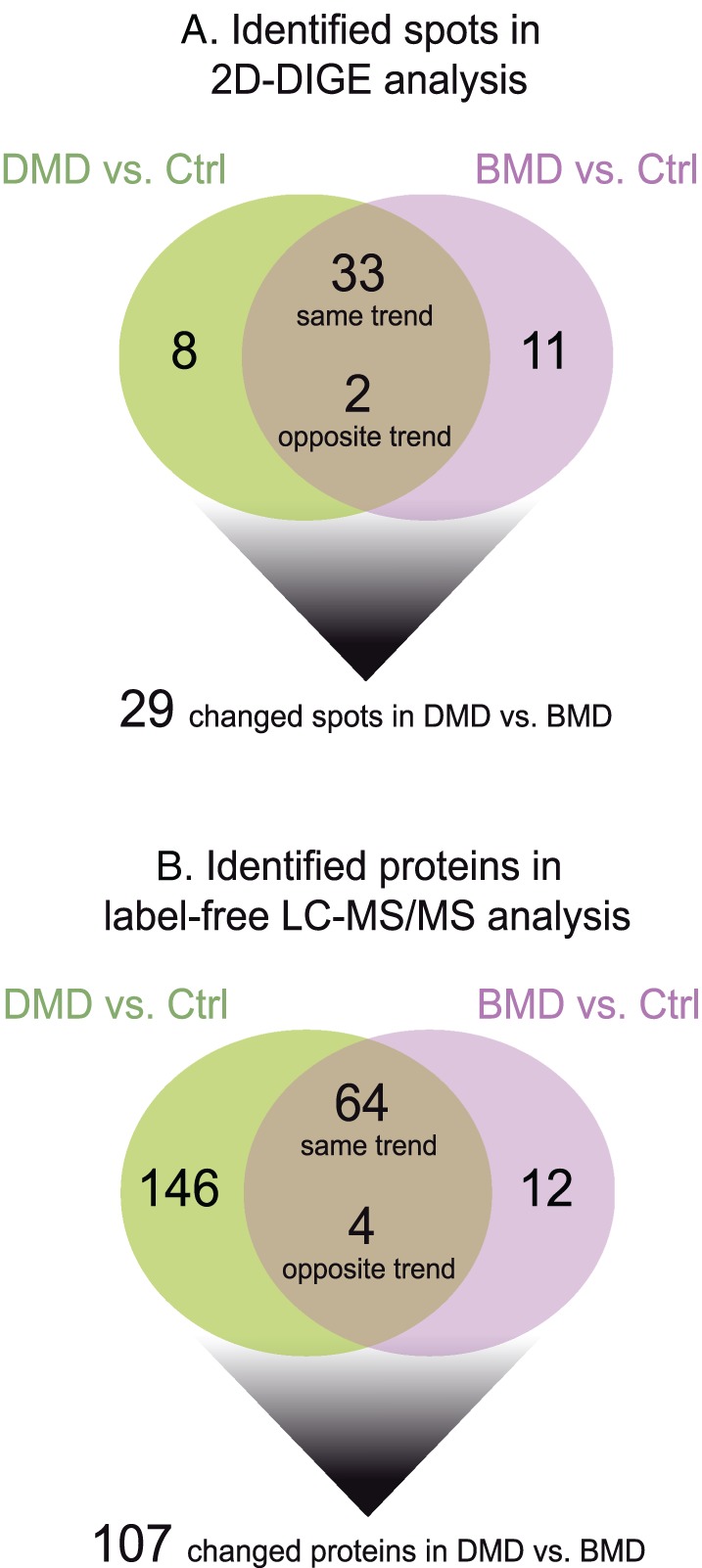
Schematic diagrams resuming findings obtained from (A) 2D‐DIGE and (B) label‐free LC‐MS/MS proteomic analyses. 2D‐DIGE, two‐dimensional difference in gel electrophoresis; BMD, Becker muscular dystrophy; DMD, Duchenne muscular dystrophy; LC‐MS/MS, liquid chromatography with tandem mass spectrometry.

Vastus lateralis muscle extracts were analysed by 2D‐DIGE and LC‐MS/MS to evaluate changes between DMD and BMD patients and between patients and healthy controls. Overall, 2D‐DIGE revealed 115 over 1000 matched spots among all gels as differentially expressed. Among them, 54 spots were identified by MS as differently expressed in DMD and BMD compared with controls (8 changed in DMD, 11 in BMD, 33 changed with the same trend both in DMD vs. ctrl and BMD vs. Ctrl, and 2 with an opposite trend in DMD vs. Ctrl compared with BMD vs. Ctrl) (*Figure* 1A). Of them, 29 spots were changed in DMD compared with BMD patients. Identification UniProtKB accession (AC) numbers are shown in Supporting Information *Table* S3. Label‐free LC‐MS/MS analyses identified 476 proteins, among them 226 were changed in DMD and BMD compared with control (146 changed in DMD, 12 in BMD, whereas 64 changed with the same trend both in DMD and BMD vs. Ctrl, and 4 counter‐regulated in DMD vs. Ctrl compared with BMD vs. Ctrl) (*Figure* 1B). Overall, 107 proteins over 226 resulted significantly changed in DMD vs. BMD patients (Supporting Information, *Table* S4). About the 30% of 2D‐DIGE identified spots were in common with the label‐free LC‐MS/MS identifications and showed overlapping expression trends. MALDI‐ToF MS spectra are shown in Supporting Information *Figure* S5.

### Proteomic analysis of Duchenne muscular dystrophy vs. Becker muscular dystrophy patients: structural/contractile proteins

**Figure 2:**
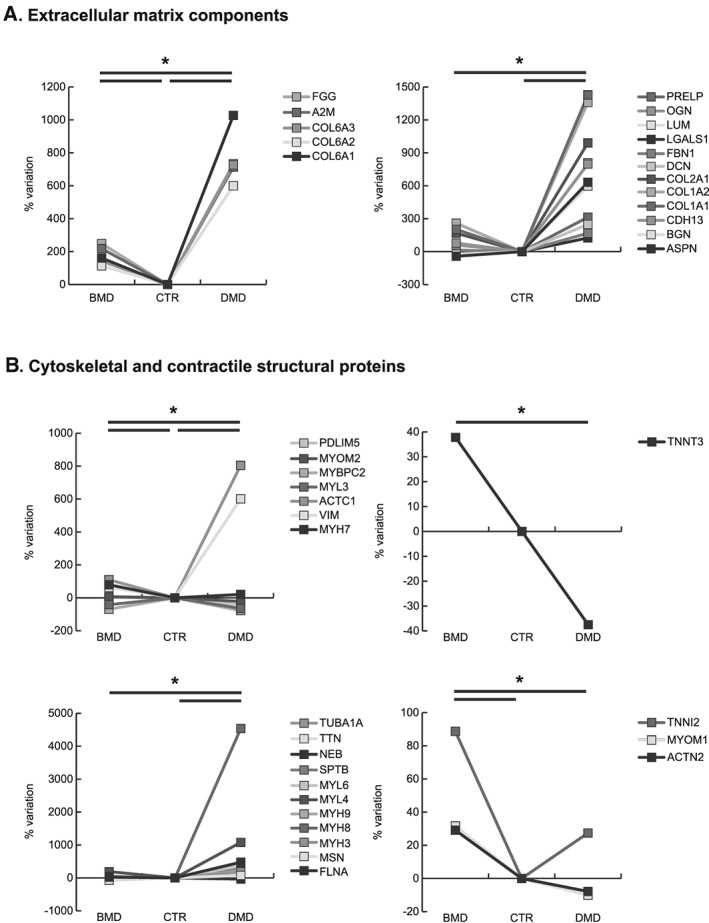
Line charts illustrating structural/contractile proteins variation (%) in BMD and DMD samples compared with controls (CTR). Proteins were divided according to the significativity of the test (ANOVA and Tukey, *n* = 3, *P*‐value < 0.01). ANOVA, analysis of variance; BMD, Becker muscular dystrophy; DMD, Duchenne muscular dystrophy.

Duchenne muscular dystrophy patients were characterized by an increment of extracellular matrix (ECM) proteins such as Asporin (ASPN), Biglycan (BGN), Cadherin‐13 (CDH13), Collagen alpha‐1(I), alpha‐2(I), and alpha‐1(II) chain (COL1A1, COL1A2, and COL2A1), Decorin (DCN), Fibrillin‐1 (FBN1), Galectin‐1 (LGALS1), Lumican (LUM), Mimecan (OGN), and Prolargin (PRELP). Several ECM proteins were up‐regulated in DMD and BMD compared with healthy controls; however, several proteins were more abundant in DMD compared with BMD. In particular, Collagen alpha‐1(VI), alpha‐2(VI), and alpha‐3(VI) chains, Collagen VI (COL6A1, COL6A2, and COL6A3 chains), Alpha‐2‐macroglobulin (A2M), and Fibrinogen gamma chain (FGG) were increased in DMD vs. BMD (*Figure* 2A).

Cytoskeletal/contractile proteins followed a similar trend being Filamin‐A (FLNA), Moesin (MSN), Embryonic myosin‐3 (MYH3), Perinatal myosin‐8 (MYH8), Non‐muscle myosin‐9 (MYH9), Embryonic muscle myosin light chain 4 (MYL4), Non‐muscle myosin light polypeptide 6 (MYL6), Spectrin beta chain (SPTB), Tubulin alpha‐1A chain (TUBA1A), Vimentin (VIM), and Alpha actin (ACTC1) increased in DMD compared either with BMD and controls. At variance, Troponin T fast skeletal muscle (TNNT3), Myosin light chain 3 (MYL3), Myosin‐binding protein C, fast‐type (MYBPC2), Nebulin (NEB), and Titin (TTN) were decreased.

Conversely, BMD patients were characterized by increased levels of Alpha‐actinin‐2 (ACTN2), Myosin‐7 (MYH7), Myomesin‐1 (MYOM1), Myomesin‐2 (MYOM2), PDZ and LIM domain protein 5 (PDLIM5) and Troponin I, fast skeletal muscle (TNNI2) compared with DMD and controls (*Figure* 2B).

### Proteomic analysis of Duchenne muscular dystrophy vs. Becker muscular dystrophy patients: metabolic proteins

**Figure 3:**
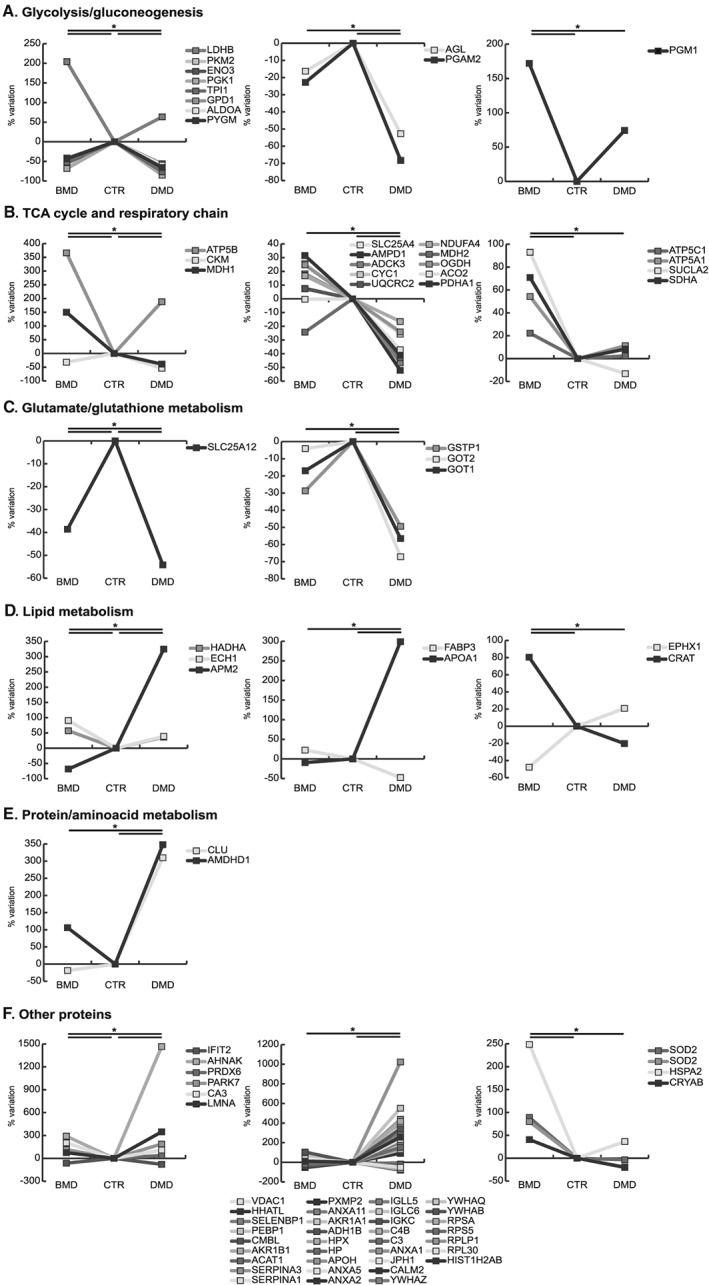
Line charts illustrating metabolic proteins variation (%) in BMD and DMD samples compared with controls (CTR). Proteins were divided according to the significativity of the test (ANOVA and Tukey, *n* = 3, *P*‐value < 0.01). ANOVA, analysis of variance; BMD, Becker muscular dystrophy; DMD, Duchenne muscular dystrophy.

Results indicated a decrement of anaerobic metabolism both in DMD and BMD compared with controls; nevertheless, the decrement of Phosphoglycerate mutase (PYGM), Aldolase A (ALDOA), cytoplasmic Glycerol‐3‐phosphate dehydrogenase [NAD(+)] (GPD1), Triosephosphate isomerase (TPI1), Phosphoglycerate kinase (PGK1), Beta enolase (ENO3), and Pyruvate kinase M1/M2 (PKM2) was more profound in DMD patients. Moreover, Phosphoglycerate mutase 2 (PGAM2) and Glycogen debranching enzyme (AGL) decreased in DMD. Conversely, BMD patients were characterized by increased levels of the glycogen metabolic pathway being protein Phosphoglucomutase (PGM1), and l‐lactate dehydrogenase B chain (LDHB) increased compared with DMD (*Figure* 3A).

The oxidative metabolism appeared severely compromised in DMD as Pyruvate dehydrogenase E1 component subunit alpha, somatic form (PDHA1), Aconitate hydratase (ACO2), 2‐oxoglutarate dehydrogenase (OGDH), and mitochondrial Malate dehydrogenase (MDH2) decreased in DMD compared with BMD and controls. Furthermore, the cytosolic Malate dehydrogenase (MDH1) was decreased in DMD, but increased in BMD. The respiratory chain and energy transfer proteins, such as NADH dehydrogenase [ubiquinone] 1 alpha subcomplex subunit 4 (NDUFA4), Cytochrome b‐c1 complex subunit 2 (UQCRC2), Cytochrome c1 (CYC1), Chaperone activity of bc1 complex‐like (ADCK3), Creatine kinase M‐type (CKM), AMP deaminase 1 (AMPD1), and ADP/ATP translocase 1 (SLC25A4), were decreased in DMD. In contrast, BMD showed increased levels of tricarboxylic acid (TCA) cycle and of respiratory chain proteins like Succinate dehydrogenase A (SDHA), Succinate Co‐A ligase beta (SUCLA2), ATP synthase alpha (ATP5A1), gamma (ATP5C1), and d subunit (ATP5B) compared with DMD and controls (*Figure* 3B).

The Calcium‐binding mitochondrial carrier protein Aralar1 (SLC25A12), involved in glutamate/glutathione metabolism, was decreased in DMD more than in BMD compared with controls. Furthermore, Aspartate aminotransferase 1 (GOT1), mitochondrial Aspartate aminotransferase (GOT2) and Glutathione *S*‐transferase P (GSTP1) were decreased in DMD (*Figure* 3C).

Concerning lipid metabolism, the Adipogenesis regulatory factor (APM2) increased in DMD and decreased in BMD whereas the Apolipoprotein A‐I (APOA1) and the Fatty acid‐binding protein (FABP3) decreased in DMD vs. BMD and controls. At variance in BMD vs. DMD and controls, Carnitine *O*‐acetyltransferase (CRAT) increased, and Epoxide hydrolase 1 (EPHX1) decreased. Proteins involved in fatty acids beta‐oxidation such as Delta(3,5)‐Delta(2,4)‐dienoyl‐CoA isomerase (ECH1) and Trifunctional enzyme subunit alpha (HADHA) increased more in BMD than in DMD and controls (*Figure* 3D).

Proteins involved in protein/amino acid metabolism like the Probable imidazolonepropionase (AMDHD1) and Clusterin (CLU) were increased in DMD vs. BMD and controls (*Figure* 3E).

Proteins regulating muscle cell development like lamin‐A/C (LMNA) and proteins involved in the transcription/translation regulation pathway like Histone H2A type 1‐B/E (HIST1H2AB), the Ribosomal proteins 60S L30 (RPL30), 60S acidic P1 (RPLP1), 40S S5 (RPS5), 40S SA (RPSA), and proteins involved in signal transduction mediated by 14‐3‐3 proteins (beta/alpha, YWHAB; theta, YWHAQ; zeta/delta, YWHAZ) were increased in DMD vs. BMD and controls. Concerning calcium handling and endoplasmic reticulum (ER)/sarcoplasmic reticulum proteins, in DMD, Calmodulin (CALM2) was increased whereas Junctophilin‐1 (JPH1) decreased. Proteins involved in the inflammation/immune response like Annexin A1 (ANXA1), Complement C3 (C3), Complement C4‐B (C4B), Ig kappa chain C region (IGKC), Ig lambda‐6 chain C region (IGLC6), and Immunoglobulin lambda‐like polypeptide 5 (IGLL5) were increased in DMD. The cytosolic stress response proteins such as Annexin A2 (ANXA2) were increased in DMD, whereas Carbonic anhydrase 3 (CA3), Protein DJ‐1 (PARK7), Peroxiredoxin‐6 (PRDX6), Alpha‐crystallin B chain (CRYAB), Heat‐shock related 70 kDa protein 2 (HSPA2), and Manganese‐containing superoxide dismutase (SOD2, 2 isoforms) were increased in BMD only. Annexin A5 (ANXA5) and Beta‐2‐glycoprotein 1 (APOH) were increased in DMD vs. controls. The transport proteins Haptoglobin (HP), Hemopexin (HPX), Albumin (ALB), and Transferrin (TF) were increased in DMD, whereas Myoglobin (MB) decreased (*Figure* 3F).

Other deregulated proteins, like the Neuroblast differentiation‐associated protein AHNAK (AHNAK), Alcohol dehydrogenase 1B (ADH1B), Alcohol dehydrogenase [NADP(+)] (AKR1A1), Annexin A11 (ANXA11), Peroxisomal membrane protein 2 (PXMP2), Alpha‐1‐antitrypsin (SERPINA1), and Alpha‐1‐antichymotrypsin (SERPINA3), were increased in DMD compared with BMD and controls, whereas Interferon‐induced protein with tetratricopeptide repeats 2 (IFIT2), mitochondrial Acetyl‐CoA acetyltransferase (ACAT1), Aldose reductase (AKR1B1), Carboxymethylenebutenolidase homolog (CMBL), Phosphatidylethanolamine‐binding protein 1 (PEBP1), Selenium‐binding protein 1 (SELENBP1), Protein‐cysteine *N*‐palmitoyltransferase HHAT‐like protein (HHATL), and Voltage‐dependent anion‐selective channel protein 1 (VDAC1) decreased (*Figure* 3F).

### Pathways analysis by immunoblotting

#### Neuronal nitric oxide synthase and detyrosinated alpha‐tubulin expression in Becker muscular dystrophy and Duchenne muscular dystrophy vs. control

**Figure 4:**
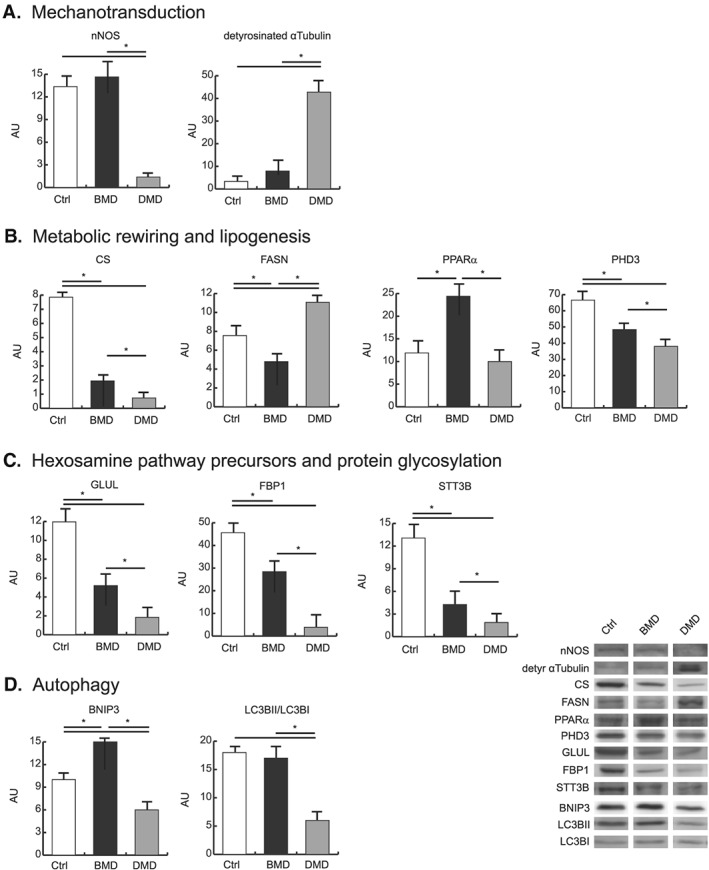
Representative histograms and immunoblot images of nNOS, detyrosinated alpha‐tubulin, CS, FASN, PPARα, PHD3, GLUL, FBP1, STT3B, BNIP3, and LC3BII/LC3BI (*n* = 3; mean ± SD; ANOVA and Tukey's test, *P* < 0.05) in BMD (black bars) and DMD (gray bars) patients and healthy controls (white bars). ANOVA, analysis of variance; BMD, Becker muscular dystrophy; CS, citrate synthase; DMD, Duchenne muscular dystrophy; FASN, fatty acid synthase; nNOS, neuronal nitric oxide synthase; PPARα, peroxisome proliferator‐activated receptor; SD, standard deviation.

Dysregulation of extracellular, cytoskeletal, and metabolic proteins is associated with mechanotransduction signalling that, ultimately, can lead to altered mechanical properties of muscle fibres and increased sarcolemmal stiffness. To support the present proteomic results, levels of detyrosinated alpha‐tubulin and nNOS, involved in mechanosensing, were assessed following some lead from present and previous proteomic results^58^, ^59^ (*Figure* 4A).

In BMD, detyrosinated alpha‐tubulin was unchanged, whereas it was increased in DMD. nNOS protein level was unchanged in BMD, whereas it was greatly decreased in DMD.

#### Metabolic rewiring through alpha‐ketoglutarate and lipogenesis

Although the dysregulated glycolytic and TCA cycle enzymes operate at a fraction of their maximal enzymatic rate, their decrease could lead to a restriction of the entire pathway. This observation opens the question of the use of an alternative energy source to sustain muscle function.

In skeletal muscle, in absence of glucose, glutamine can act as a nitrogen donor for the synthesis of proteins and nucleosides^60^ and be converted to alpha‐ketoglutarate (α‐KG), thereby supporting anaplerotically the TCA cycle, or reductively transformed by cytosolic isocitrate dehydrogenase (IDH1) to citrate.^61^, ^62^, ^63^, ^64^ To determine the fate of α‐KG, a set of selected molecules was investigated by immunoblotting (*Figure* 4B). In particular, our attention was focused on the reductive/lipogenic pathway (citrate synthase, CS; fatty acid synthase, FASN) and related regulators (peroxisome proliferator‐activated receptor, PPARα). CS was more decreased in DMD than in BMD, compared with controls. Conversely, FASN was increased in DMD whereas decreased in BMD vs. controls. PPARα was increased in BMD vs. DMD and controls. These results, combined with proteomic data, suggest that DMD patients are characterized by a metabolic rewiring at the level of α‐KG, which leads to a shortage of glutamate‐derived molecules with protective functions (such as glutamine, glutathione, and polyamines), and this could trigger lipogenesis causing lipotoxicity over time.

From proteomic results, we observed increased levels of enzymes involved in fatty acid beta‐oxidation, more pronounced in BMD than in DMD patients (*Figure* 3D). Conversely, in DMD patients, this metabolic adaptation was absent with increased lipogenesis and lipid oxidation. To this point, we assessed the levels of Prolyl hydroxylase domain‐containing protein 3 (PHD3), a selective repressor of beta‐oxidation.^65^ PHD3 levels were more decreased in DMD than in BMD compared with controls (*Figure* 4B), suggesting that PHD3 decrement in DMD supports the increase of fatty acids oxidation and the maintenance of lipid synthesis.

#### Hexosamine pathway and protein glycosylation

The second question opened by proteomic results is the link between glucose depletion and ER stress. Because glucose is essential for protein glycosylation, if intracellular glucose levels decrease, the carbohydrate chain that is frequently used to glycosylate proteins cannot be assembled, leading to improper protein glycosylation, protein misfolding, activation of the unfolded protein response, and increased ER stress.^66^ The insufficient supply of substrates (glucose and glucosamine), which is supported by the decrement of glutamine synthetase (GLUL) and the gluconeogenetic enzyme fructose‐1,6‐bisphosphatase (FBP1) in both BMD and DMD (*Figure* 4C), can lead to impaired protein glycosylation contributing to ER stress and the activation of unfolded protein response. Overall, these concepts lead to analyse pathways controlling glycosylation processes.

The dolichyl‐diphosphooligosaccharide/protein glycosyltransferase subunit STT3B of the *N*‐oligosaccharyl transferase complex decreased more in DMD than in BMD compared with controls (*Figure* 4C), indicating an impairment of *N*‐linked protein glycosylation in the ER in both disorders.

#### Autophagy

Autophagy is emerging as an important process that limits muscle damage. Cytoskeletal remodelling is associated with the activation of the autophagic process to clear damaged proteins. Immunoblottings indicated that the energy costly process of autophagy was decreased in DMD whereas it was maintained in BMD (*Figure* 4D), as suggested by the expression of the autophagy‐inducing protein BCL2/adenovirus E1B 19 kDa protein‐interacting protein 3 (BNIP3), which was decreased in DMD and increased in BMD.

Concerning the microtubule‐associated proteins 1A/1B light chain 3B (LC3B), a protein involved in autophagosome assembly, the ratio of the lipidated (activated) form (LC3BII) over the delipidated one (LC3BI) was assessed (*Figure* 4D). LC3B‐II/LC3B‐I decreased in DMD compared with BMD and controls.

### Validation of proteomics results

The protein identification was validated performing a random analysis via immunoblotting of seven identified proteins (Supporting Information, *Figure* S6).

### Verification of the dystrophin‐associated protein complex status

**Figure 5:**
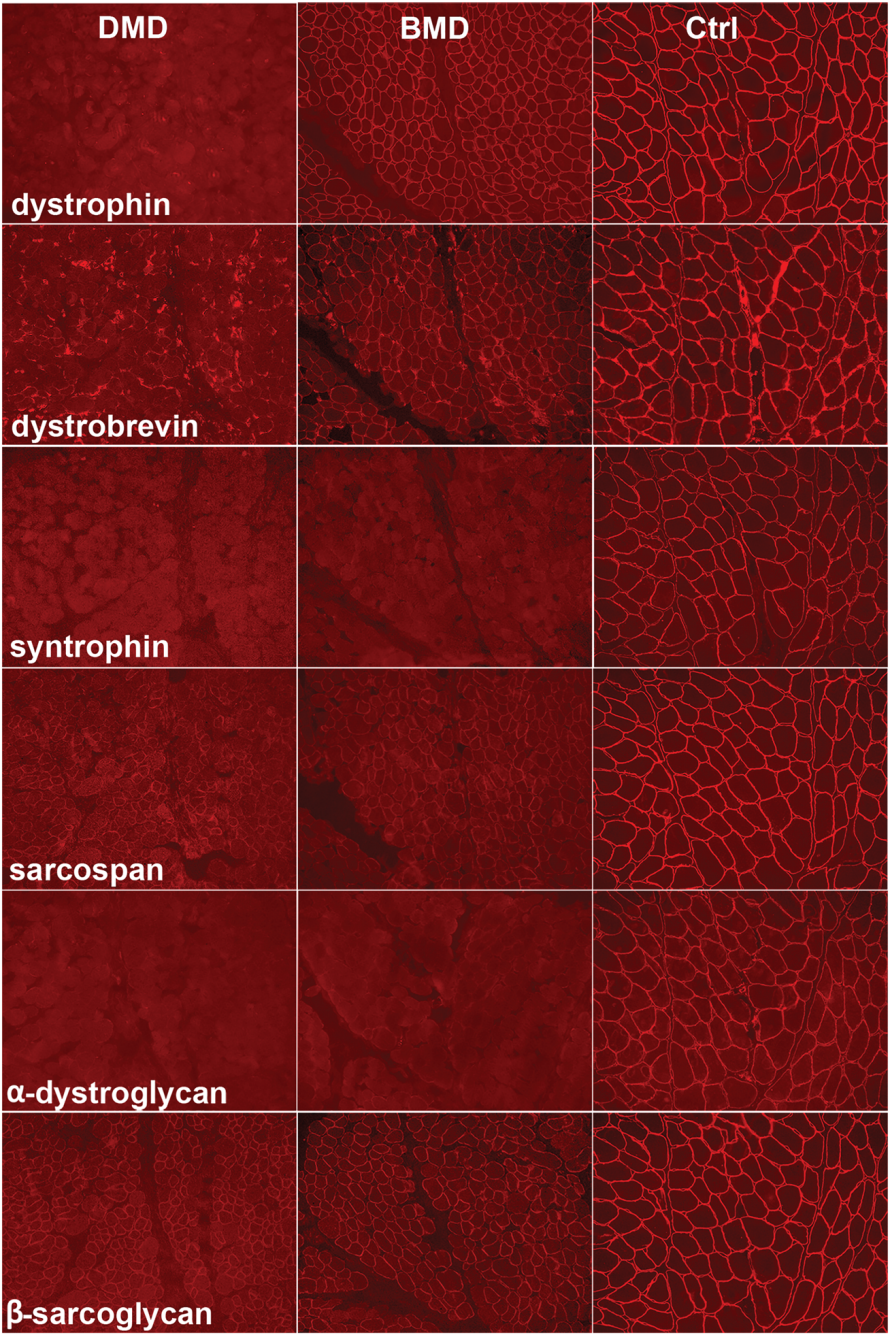
Immunostaining of dystrophin and dystrophin‐associated proteins (alpha‐dystroglycan, beta‐sarcoglycan, alpha‐syntrophin, alpha‐dystrobrevin, and sarcospan), in consecutive sections of muscle from a DMD patient (left column of the panel), a BMD patient (central column), and a control (right column). BMD, Becker muscular dystrophy; DMD, Duchenne muscular dystrophy.

To compare primary vs. secondary effects of dystrophin mutations, immunofluorescence microscopy of dystrophin, alpha‐dystroglycan, beta‐sarcoglycan, alpha‐syntrophin, alpha‐dystrobrevin, and sarcospan is shown in *Figure* 5. Immunostaining results indicated absence of signals in DMD and reduced levels in BMD.

## Discussion

The lack of dystrophin destabilizes the sarcolemma, making muscle fibres susceptible to injury during contraction,^67^ and impacts overall on muscle system by disrupting dystrophin‐associated protein complex, thus affecting calcium homeostasis and, consequently, the contraction relaxation processes. Furthermore, it disrupts muscle metabolism as enzymes are directly located within fibres and signals are transferred to intermyofibrillar mitochondria contributing to their functional decline.^34^, ^35^ Results from the present study provide an overview of the players strictly dependent on dystrophin that contribute to the loss of muscle function. Our study, through the analysis of muscle biopsies of DMD and BMD patients compared with controls, was designed to provide a picture of dysregulated proteins and to identify which, beside dystrophin, are the critical molecules involved in the disruption of the well‐tuned molecular organization at the basis of muscle function.

**Figure 6:**
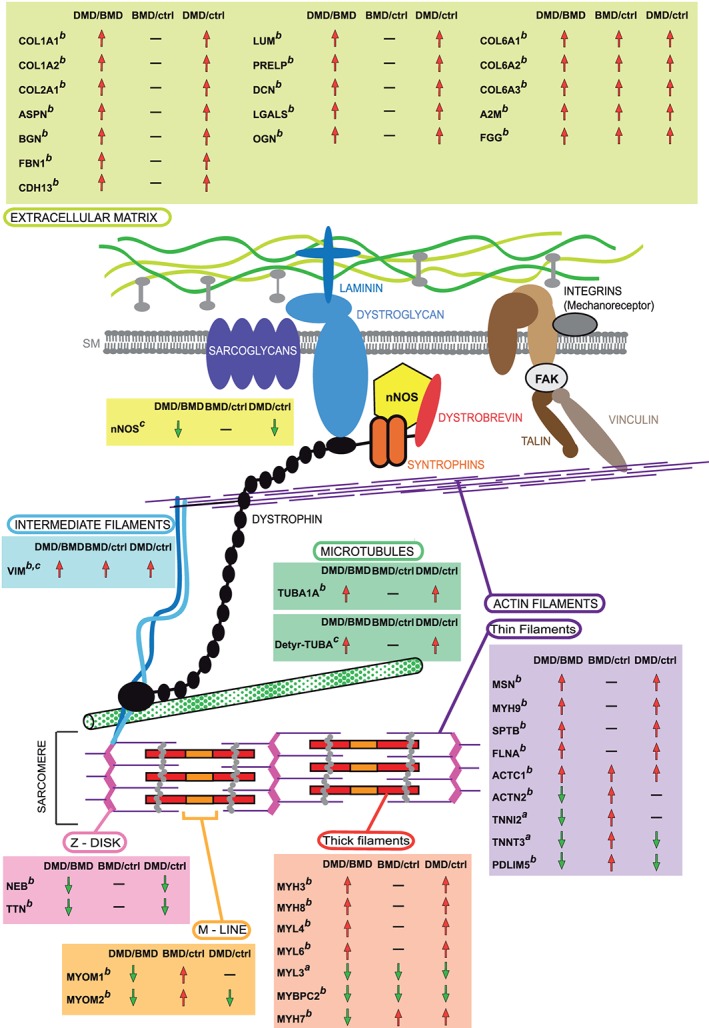
Graphical representation of the extracellular, cytoskeletal, and sarcomeric structures in which deregulated proteins in DMD vs. BMD are indicated. Rectangles contain gene names of dysregulated proteins observed. Superscripts indicate results obtained by (a) 2D‐DIGE, (b) label‐free LC‐MS/MS, and (c) immunoblotting. Arrows indicate the protein trend between DMD and BMD patients and between patients and healthy controls. 2D‐DIGE, two‐dimensional difference in gel electrophoresis; BMD, Becker muscular dystrophy; DMD, Duchenne muscular dystrophy; LC‐MS/MS, liquid chromatography with tandem mass spectrometry.

Our results indicate that BMD patient muscle is characterized by minor changes in the ECM proteome and is able to maintain the mechanotransduction signalling with reduced changes in cytoskeletal and contractile proteins (*Figure* 6). This partial homeostasis is supported by both, detyrosinated tubulin and nNOS levels, which are unchanged in BMD vs. controls. These two molecules have been selected to demonstrate the contribution of ECM/contractile dysregulation to BMD vs. DMD phenotype. In fact, tubulin detyrosination is known to regulate mechanotransduction by modifying the mechanical properties of the cytoskeleton, with consequent increase in cytoskeletal stiffness and initiation of mechanosignalling that leads to the activation of NADPH oxidase 2 and subsequent ROS production. The latter promotes the influx of Ca2+ through mechano‐sensitive channels.^30^, ^68^, ^69^, ^70^

Neuronal nitric oxide synthase is known to interact with dystrophin, and it is localized near the sarcolemma, where it controls vasoregulation and oxygen delivery in working muscles. Dystrophin deficiency misplaces nNOS from the sarcolemma to the cytosol where the residual amount of protein is also greatly reduced^25^, ^71^, ^72^, ^73^, ^74^ contributing to impaired contraction, failed muscle regeneration, and misregulated inflammatory response.^75^

**Figure 7:**
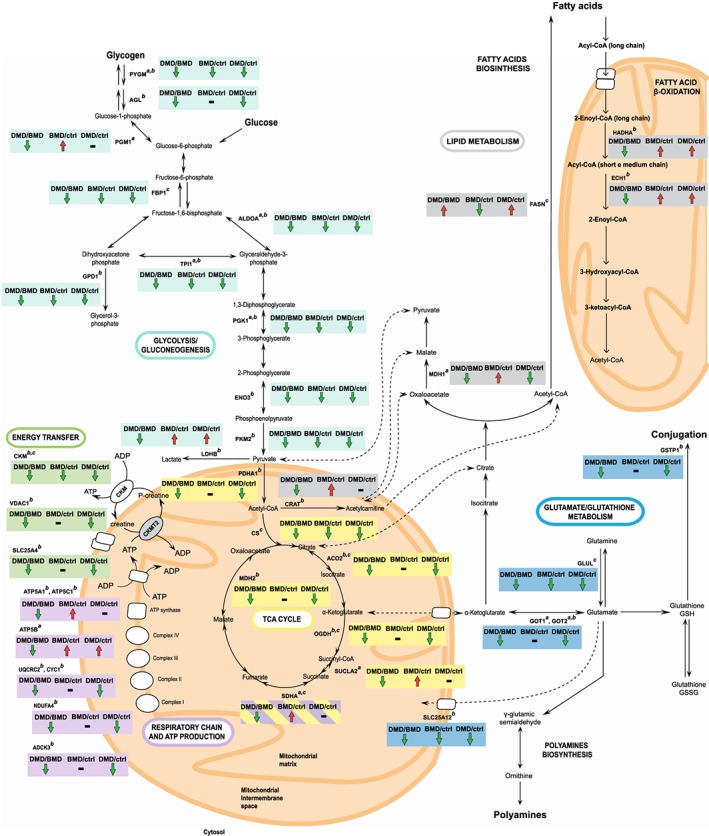
Schematic representation of metabolic enzymes dysregulated in DMD vs. BMD patients. Arrows indicate the protein trend between DMD and BMD patients and between patients and healthy controls. Superscripts indicate results obtained by (a) 2D‐DIGE, (b) label‐free LC‐MS/MS, and (c) immunoblotting. 2D‐DIGE, two‐dimensional difference in gel electrophoresis; BMD, Becker muscular dystrophy; DMD, Duchenne muscular dystrophy; LC‐MS/MS, liquid chromatography with tandem mass spectrometry.

Another characteristic specific of DMD is the change in muscle metabolism: the entire glycolytic/glycogen pathway and respiratory chain complex I activity are severely compromised with a signal for fatty acids accumulation (provided by a decrement of ACO2 and increment of APM2 and FASN) and for fatty acid beta‐oxidation (supported by PDH3 levels, dramatically decreased in DMD^65^). Moreover, the mitochondrial‐cytosolic energy exchange through ADP/ATP cycling and creatine/phosphocreatine shuttling resulted impaired in DMD (decreased SLC25A4, CKM, and VDAC1), contributing to exacerbate oxidative stress.^76^ Conversely, in BMD patients, lipids utilization was enhanced (increased signalling of PPARα), and TCA cycle and respiratory chain were maintained with increments of complex II and ATP synthase subunits that can contribute to provide energy (*Figure* 7). The metabolic adaptation is supported by increment of HADHA, which catalyses three steps of long chain fatty acid beta‐oxidation in mitochondria and allows BMD patients to increase their mitochondrial capacity of beta‐oxidation improving the utilization of long chain fatty acid as an energy source and ketogenesis.^77^

Furthermore, increased LDHB expression, fuelled by increased cytosolic MDH1 levels, modulates the mitochondrial activity and confers resistance to muscle damage by maintaining lower mitochondrial membrane potential, decreased ROS production, and decreased oxygen consumption keeping ATP at reasonable levels. In fact, it has been described that in cells LDHB competes with the mitochondrial NADH/NAD+ shuttle systems to regenerate NAD+.^78^ Therefore, the overexpression of LDHB likely limits the availability of both pyruvate and NADH in the mitochondria, thereby decreasing respiration and respiratory chain activity and ROS production.^79^ Overexpression of these key ‘Warburg effect’ enzymes decreases mitochondrial respiration while maintaining ATP production, thus contributing to the protective role of these proteins. We propose fatty acids as the main fuel source to meet the energy demands in BMD muscles, while lactate production could modulate respiratory chain activity to limit ROS production. This phenotypical characteristic of BMD patients allows them to preserve their muscle function.

Conversely, in DMD, the expression of MDH1 is decreased, and LDHB expression is lower than in BMD, leaving the muscle unable to control ROS production, hence exacerbating the pathophysiological processes associated with DMD. Muscle tissue cannot maintain high levels of cellular ATP through increased glycolytic pathway flux, being the majority of glycolytic enzymes decreased. Further studies will be required to precisely address this issue including the study of post‐translational modifications that could lead to an accumulation of not functional intermediates; studies are in progress in our lab in this direction.

Another characteristic feature of DMD muscles is the decrement of mitochondrial aconitase (ACO2) and the increment of FASN. The former is central to carbohydrate and energy metabolism and is responsible for the interconversion of citrate to isocitrate as part of the citric acid cycle, the latter regulates de novo lipid biosynthesis. We hypothesize that the ROS increment induced by decrement of complex I and high energy phosphate shuttling, not supported by the increment of complex II, causes the decrement of ACO2^80^ with consequent restriction of the metabolic flux through the TCA cycle and accumulation of metabolic precursors for fatty acid synthesis. At variance, in BMD, the greater induction of LDHB makes them able to keep ROS production under control, and the increment of HADHA allows the utilization of fatty acids for FADH2 generation making available this cofactor for complex II, providing a metabolic rewiring for muscle function maintenance.

We are aware of the limitation of the present study due to the restricted number of available samples that hampered verification needed to confirm ACO2, FASN, HADHA, LDHB as central node of metabolic rewiring that characterizes BMD, in conjunction with ECM matrix protein and detyrosinated tubulin that characterizes DMD. Work is in progress to recruit an independent set of samples to verify our results.

In conclusion, this study indicates the mechanosensing and metabolic disruption as central nodes of DMD/BMD phenotypes and identifies dysregulation in the ECM proteome composition in DMD. The latter condition is characterized by a metabolic restriction at the level of α‐KG that leads to a shortage of glutamate‐derived molecules with protective functions triggering lipogenesis and lipotoxicity over time. In BMD, the metabolic rewiring leads to energy preservation supporting autophagy and cell renewal, thus retaining muscle function. From the present study, it appears that to support muscle regeneration will require intervention at metabolic level decreasing ROS production, favouring LDHB and HADHA increment, thus lowering lipogenesis by controlling PHD3 expression.

## Author disclosure statement

No competing financial interests exist.

## Supporting information

Table S1 Characteristics of patients involved in the proteomic studies

Figure S2 Western blot full images

Table S3. Changed spots in 2D‐DIGE analysis

Table S4. Changed proteins in label‐free LC‐MS/MS analysis

Figure S5. List of annotated spectra for MALDI‐ToF identified proteins

Figure S6. Protein validation by immunoblotting. Representative histograms and immunoblot images of mitochondrial aconitase (ACO2), creatine kinase Mtype (CKM), L‐lactate dehydrogenase A chain (LDHA), succinate dehydrogenase flavoprotein subunit (SDHA), alpha‐crystallin B chain (CRYAB) and vimentin (VIM) (*n* = 3; mean ± S.D.; ANOVA and Tukey's test, *P* < 0.05) in BMD (black bars) and DMD (gray bars) patients and healthy controls (white bars).
