# Supplementary material for: Comparative proteomic analyses of Duchenne muscular dystrophy and Becker muscular dystrophy muscles: changes contributing to preserve muscle function in Becker muscular dystrophy patients
Source: J Cachexia Sarcopenia Muscle. 2020 Jan 28;11(2):547–63. doi: 10.1002/jcsm.12527 (PMC7113522; doi:10.1002/jcsm.12527)
Supplement: Supplementary file 1 — Table S1 Characteristics of patients involved in the proteomic studies [file JCSM-11-547-s001.pdf]

Table S1 Characteristics of patients involved in the proteomic studies

Diagnosis	ID	Age	Genotyping (if present)	Ambulant	Treatment with corticosteroids	B blockers	ACE inhibitors
**DMD**	1	1	del 3-12	yes	no	no	no
**DMD**	2	3		yes	no	no	no
**DMD**	3	6	del 50	yes	no	no	no
**DMD**	4	5		yes	no	no	no
**DMD**	5	7	del 45	yes	no	no	no
**DMD**	6	8	del 3-6	yes	no	no	no
**DMD**	7	3	del 45	yes	no	no	no
**DMD**	8	5		yes	no	no	no
**DMD**	9	1	del 44	yes	no	no	no
**DMD**	10	1		yes	no	no	no
**DMD**	11	2	del 45-52	yes	no	no	no
**DMD**	12	9 months	del 5-9	yes	no	no	no
**DMD**	13	2	del 22-29	yes	no	no	no
**DMD**	14	3	c.5209C>T	yes	no	no	no
**DMD**	15	6	del 44	yes	no	no	no
**BMD**	16	11		yes	no	no	no
**BMD**	17	3	dupl.10-34 e 45-48	yes	no	no	no
**BMD**	18	4	del 3-8	yes	no	no	no
**BMD**	19	6	del 48	yes	no	no	no
**BMD**	20	6		yes	no	no	no
**BMD**	21	11	del 16-29	yes	no	no	no
**BMD**	22	7	del 48-51	yes	no	no	no
**BMD**	23	2		yes	no	no	no
**BMD**	24	3		yes	no	no	no
**BMD**	25	4		yes	no	no	no
**BMD**	26	3	MLPA negative	yes	no	no	no
**BMD**	27	3 months	del 3-4	yes	no	no	no
**BMD**	28	3	del 48-51	yes	no	no	no
**BMD**	29	5	del 48	yes	no	no	no
**BMD**	30	9	del 48	yes	no	no	no
**CTRL**	31	5					
**CTRL**	32	3					
**CTRL**	33	9					
**CTRL**	34	4					
**CTRL**	35	6					
**CTRL**	36	10					
**CTRL**	37	6					
**CTRL**	38	7					
**CTRL**	39	5					
**CTRL**	40	5					
**CTRL**	41	11					
**CTRL**	42	13					
**CTRL**	43	4					
**CTRL**	44	6					
**CTRL**	45	6					
