# Supplementary material for: Comparative proteomic analyses of Duchenne muscular dystrophy and Becker muscular dystrophy muscles: changes contributing to preserve muscle function in Becker muscular dystrophy patients
Source: J Cachexia Sarcopenia Muscle. 2020 Jan 28;11(2):547–63. doi: 10.1002/jcsm.12527 (PMC7113522; doi:10.1002/jcsm.12527)

Figure S2 Western blot full images

BNIP3 (30 KDa)

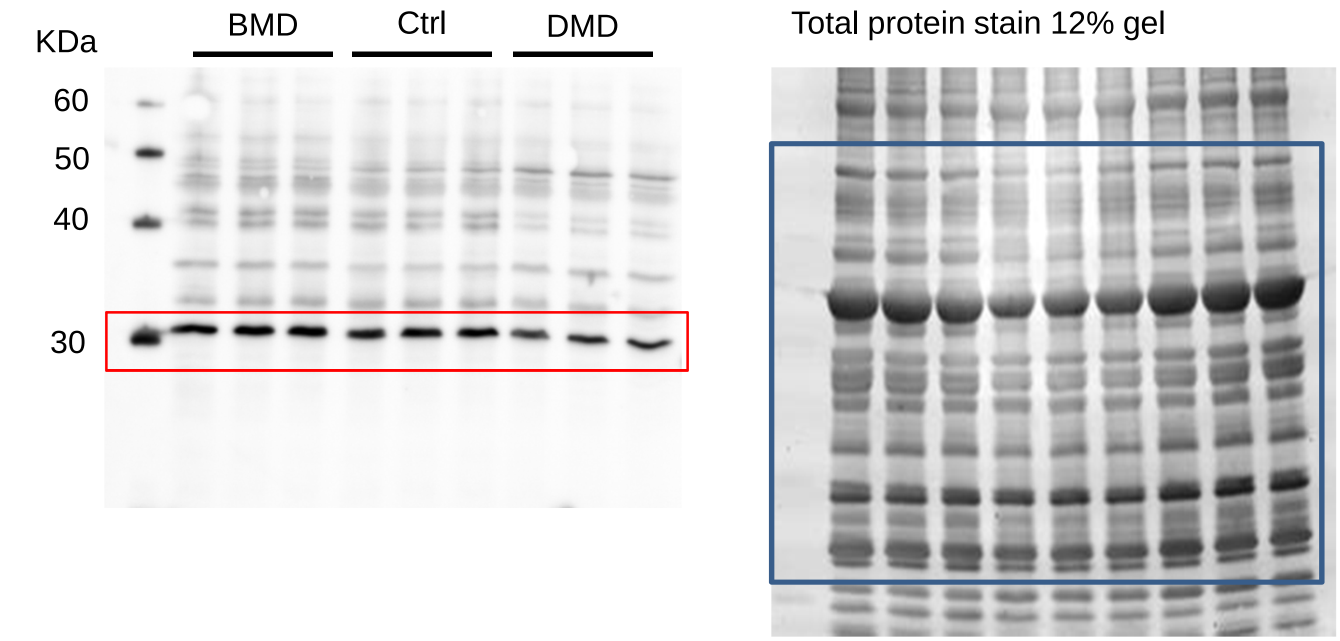

LC3B (14 and 16 KDa)

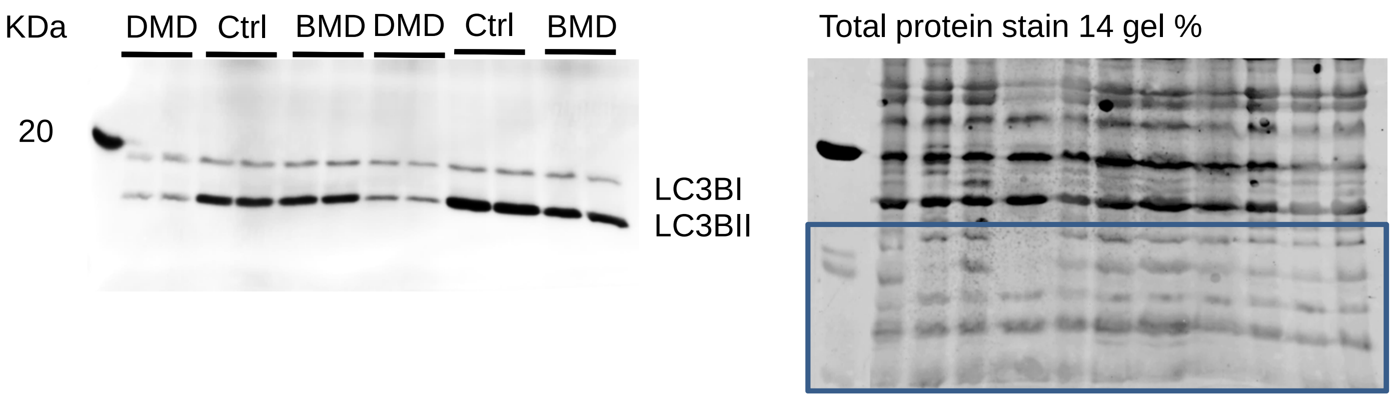

PHD3 (27 KDa)

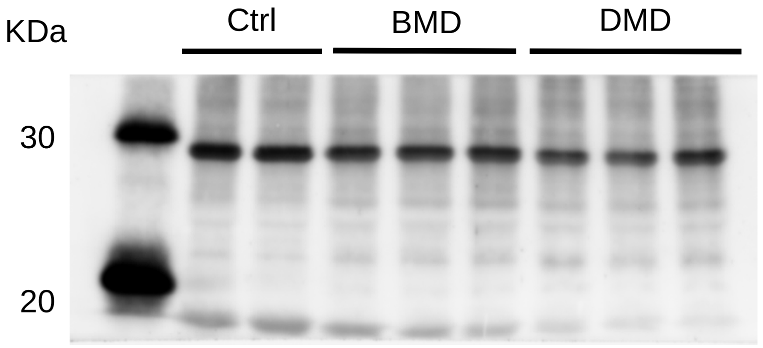

Total protein stain 10% gel

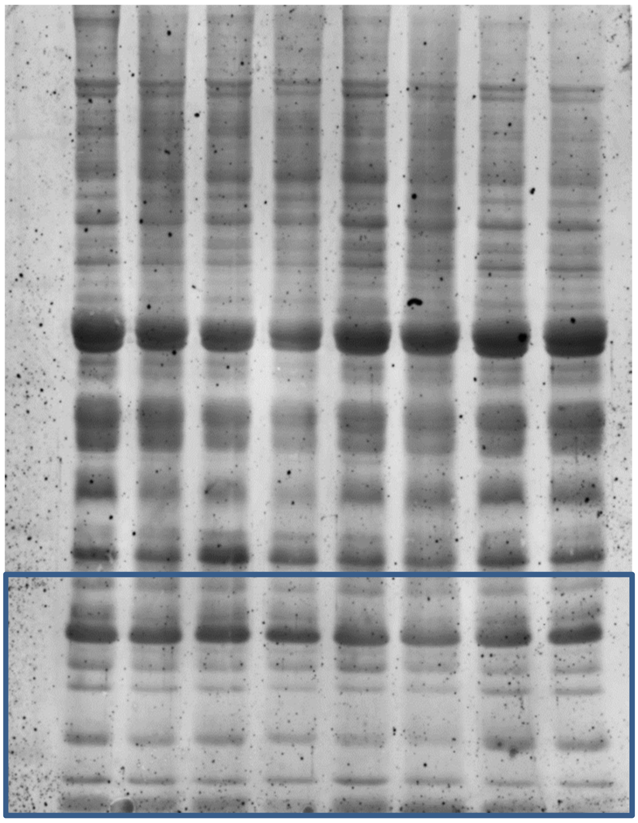

nNOS (155 KDa)

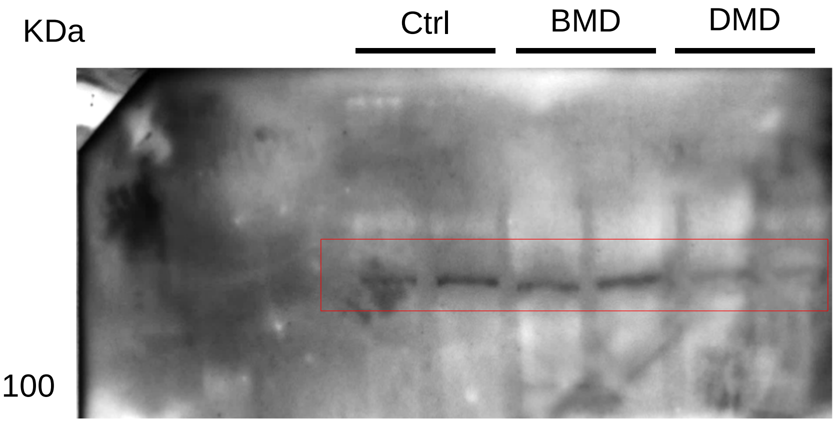

Total protein stain 10% gel

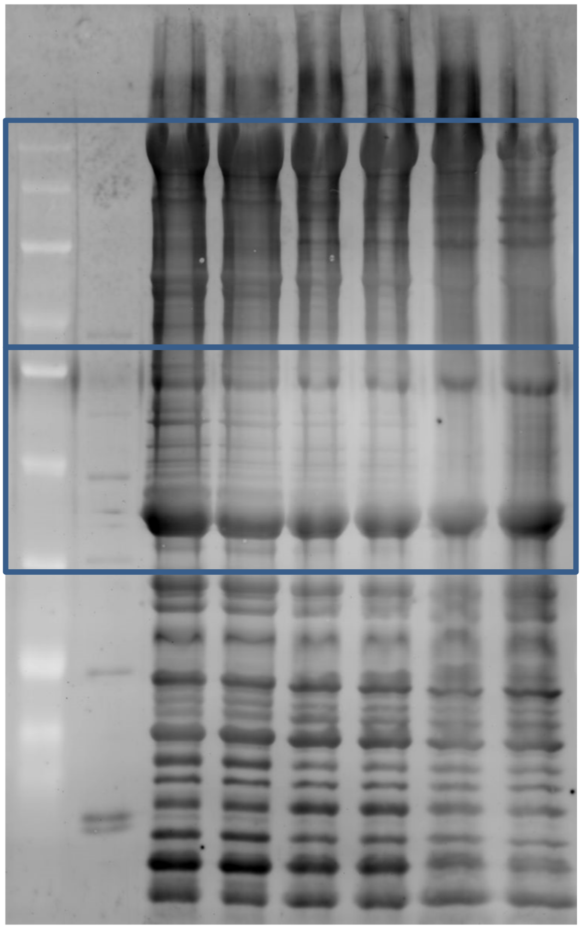

de-Tyr alpha-tubulin (51 KDa)

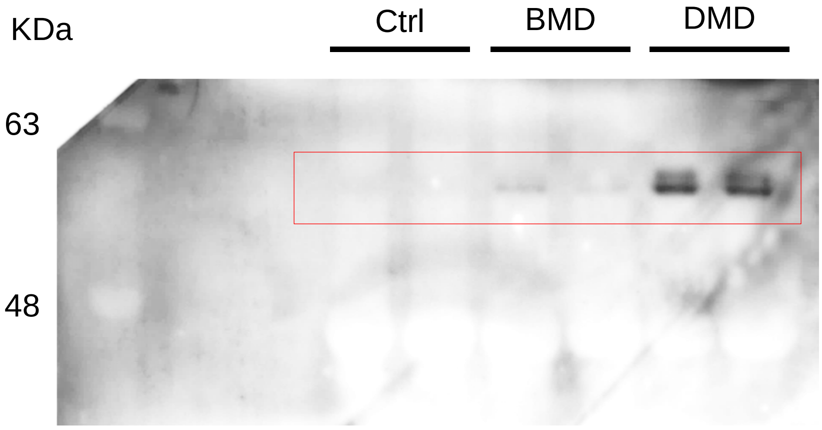

FASN (270 KDa)

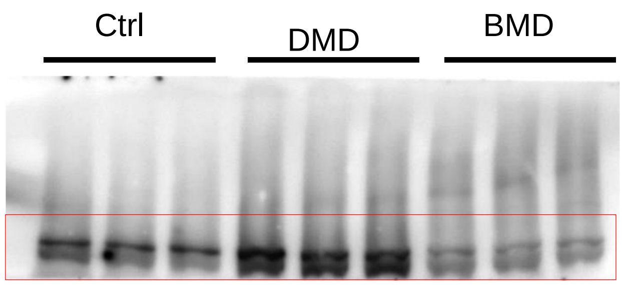

Total protein stain gel 6%

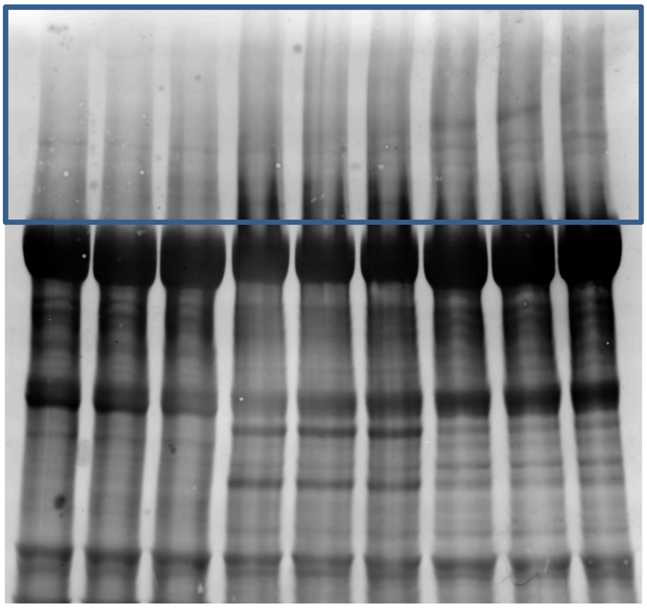

PPARα (55 KDa)

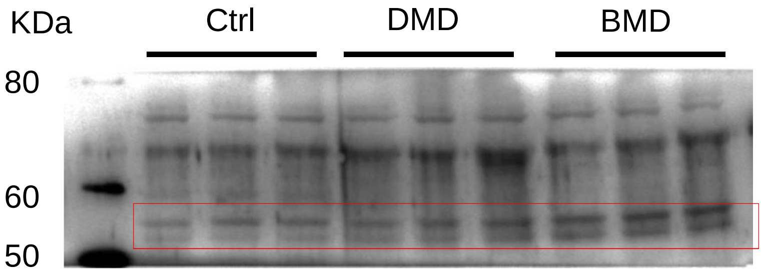

Total protein stain gel 8-14%

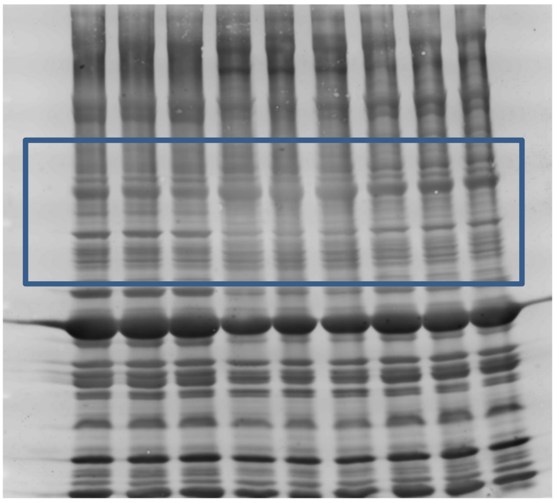

CS (52 KDa)

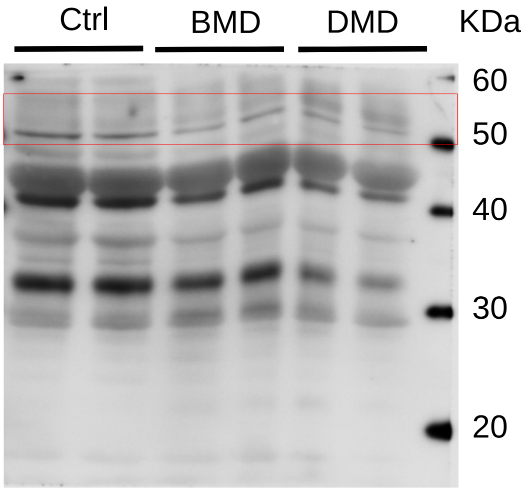

Total protein stain gel 12%

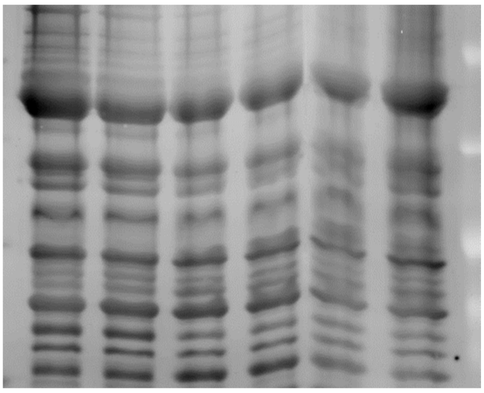

STT3B (94 KDa)

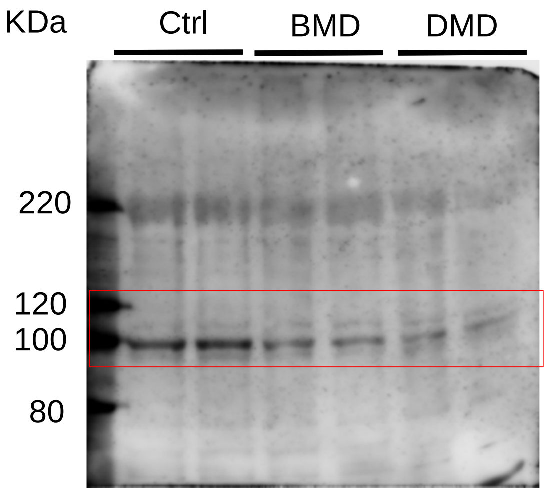

Total protein stain gel 12%

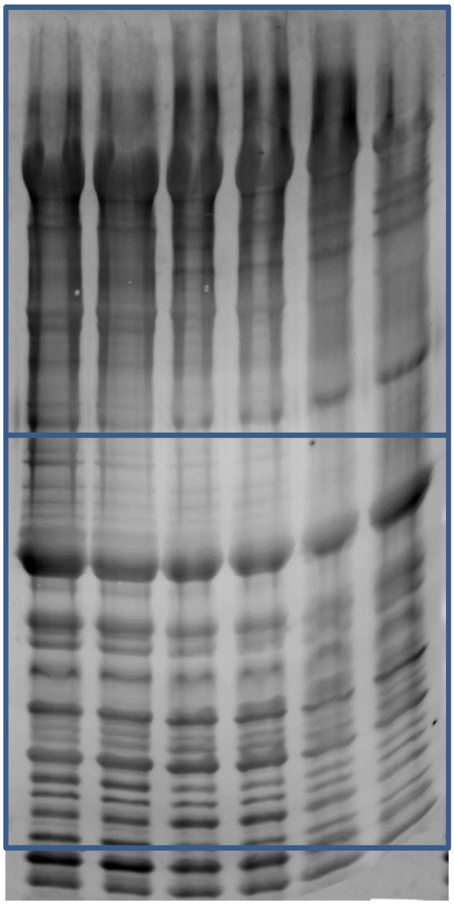

GLUL (43 KDa)

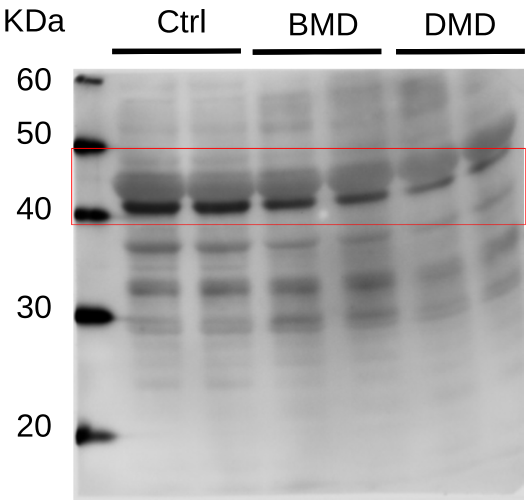

FBP1 (36 KDa)

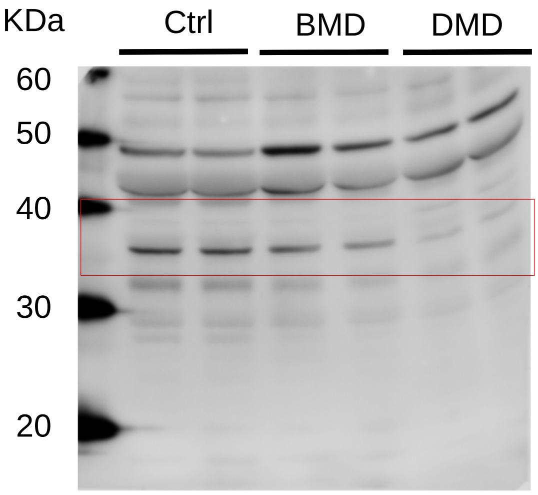

Supplement: Supplementary file 2 — Figure S2 Western blot full images [file JCSM-11-547-s002.pdf]
