# Supplementary material for: Comparative proteomic analyses of Duchenne muscular dystrophy and Becker muscular dystrophy muscles: changes contributing to preserve muscle function in Becker muscular dystrophy patients
Source: J Cachexia Sarcopenia Muscle. 2020 Jan 28;11(2):547–63. doi: 10.1002/jcsm.12527 (PMC7113522; doi:10.1002/jcsm.12527)
Supplement: Supplementary file 3 — Table S3. Changed spots in 2D‐DIGE analysis [file JCSM-11-547-s003.pdf]

Table S3. Changed spots in 2D-DIGE analysis.

a. Spot significantly changed (ANOVA + Tukey, p<0.01) in DMD vs. BMD

Gene symbol	UniprotKB AC n°	Description	Molecular function	Av. volumes			Av.ratio			% variation			ANOVA	Tukey HSD			Kruskal-Wallis	MW [kDa]	pI	Searched peptides	Matched peptides	Mascot Score	sequence coverage [%]
				CTRL	BMD	DMD	DMD/ctrl	BMD/ctrl	DMD/BMD	DMD/ctrl	BMD/ctrl	DMD/BMD		CTRL-DMD	CTRL-BMD	BMD-DMD							
MYL3	P08590	slow skeletal ventricular myosin alkali light chain 3	Structural and contractile proteins (thick filaments)	189,675	110,602	67,000	0,353	0,583	0,606	-64,7	-41,7	-39,4	0,000	0,000	0,000	0,000	0,000	22,1	4,9	23	12	194	58,5
TNNT3	P45378	troponin T, fast skeletal muscle	Structural and contractile proteins (thin filaments)	32,501	44,786	20,301	0,625	1,378	0,453	-37,5	37,8	-54,7	0,000	0,005	0,001	0,000	0,000	28,4	9,8	22	9	125	38,1
TNNI2	P48788	troponin I, skeletal, fast	Structural and contractile proteins (thin filaments)	19,299	36,411	24,590	1,274	1,887	0,675	27,4	88,7	-32,5	0,000	0,301	0,000	0,004	0,001	21,6	10	15	9	101	51,1
PYGM	P11217	glycogen phosphorylase	Glycogen/glucose metabolism	0,860	0,546	0,263	0,306	0,635	0,482	-69,4	-36,5	-51,8	0,000	0,000	0,001	0,001	0,000	97,5	6,5	43	34	382	47,5
ALDOA	P04075	aldolase A	Glycogen/glucose metabolism (Glycolysis)	95,741	55,909	35,929	0,375	0,584	0,643	-62,5	-41,6	-35,7	0,000	0,000	0,000	0,003	0,000	39,9	9,2	43	22	279	65,4
ALDOA	P04075	aldolase A	Glycogen/glucose metabolism (Glycolysis)	75,528	58,162	31,487	0,417	0,770	0,541	-58,3	-23,0	-45,9	0,000	0,000	0,008	0,000	0,000	39,9	9,2	34	21	288	65,4
PGK1	P00558	phosphoglycerate kinase 1	Glycogen/glucose metabolism (Glycolysis)	14,059	7,834	2,895	0,206	0,557	0,370	-79,4	-44,3	-63,0	0,000	0,000	0,000	0,000	0,000	45	9,2	26	15	169	44,4
TPI1	P60174	triosephosphate isomerase	Glycogen/glucose metabolism (Glycolysis)	25,219	13,649	9,169	0,364	0,541	0,672	-63,6	-45,9	-32,8	0,000	0,000	0,000	0,008	0,000	26,9	6,5	19	14	247	57,8
TPI1	P60174	triosephosphate isomerase	Glycogen/glucose metabolism (Glycolysis)	11,955	7,133	2,794	0,234	0,597	0,392	-76,6	-40,3	-60,8	0,000	0,000	0,000	0,000	0,000	26,8	6,5	38	12	145	55,6
PGM1	P36871	phosphoglucomutase 1	Glycogen/glucose metabolism	0,640	1,740	1,114	1,742	2,720	0,640	74,2	172,0	-36,0	0,000	0,013	0,000	0,001	0,000	61,7	6,3	22	17	217	35,9
PGAM2	P15259	phosphoglycerate mutase 2	Glycogen/glucose metabolism (Glycolysis)	14,868	11,483	4,715	0,317	0,772	0,411	-68,3	-22,8	-58,9	0,000	0,000	0,072	0,000	0,000	28,9	9,5	25	18	241	59,3
MDH1	P40925	cytosolic malate dehydrogenase	TCA cycle	1,414	3,534	1,938	1,370	2,500	0,548	37,0	150,0	-45,2	0,000	0,001	0,000	0,000	0,000	31,9	8,8	24	11	126	46,4
SDHA	P31040	succinate dehydrogenase [ubiquinone] flavoprotein subunit, mitochondrial prec	TCA cycle	0,656	1,122	0,711	1,083	1,709	0,634	8,3	70,9	-36,6	0,000	0,839	0,000	0,000	0,001	68,3	9,4	26	19	273	46,3
SUCLA2	Q9P2R7	succinate-CoA ligase, ADP-forming, beta subunit	TCA cycle	0,987	1,905	0,857	0,868	1,929	0,450	-13,2	92,9	-55,0	0,000	0,735	0,000	0,000	0,000	48,4	6,8	22	10	100	18,8
ATP5B	Q0QEN7	mitochondrial ATP synthase, H+ transporting F1 complex beta subunit	Respiratory chain/ATP synthesis	0,617	2,876	1,779	2,885	4,664	0,619	188,5	366,4	-38,1	0,000	0,000	0,000	0,000	0,000	48,1	4,8	30	21	238	49,4
GOT1	P17174	aspartate aminotransferase 1	aminoacid metabolism	2,570	2,134	1,119	0,435	0,830	0,524	-56,5	-17,0	-47,6	0,000	0,000	0,295	0,004	0,002	46,4	6,6	22	13	190	34,1
GOT2	P00505	aspartate aminotransferase 2 precursor	aminoacid metabolism	1,735	1,313	0,455	0,263	0,757	0,347	-73,7	-24,3	-65,3	0,000	0,000	0,097	0,000	0,000	47,9	9,1	30	14	155	48
CA3	P07451	carbonic anhydrase 3	response to stress	20,663	62,706	43,361	2,098	3,035	0,691	109,8	203,5	-30,9	0,000	0,000	0,000	0,000	0,000	29,7	7,1	33	9	135	45,6
PRDX6	P30041	peroxiredoxin 6	response to stress	2,280	4,135	3,162	1,387	1,814	0,765	38,7	81,4	-23,5	0,000	0,006	0,000	0,001	0,000	25,1	6	22	12	187	49,1
CRYAB	P02511	alpha-crystallin B chain	response to stress	0,885	1,245	0,707	0,799	1,407	0,568	-20,1	40,7	-43,2	0,000	0,121	0,001	0,000	0,001	20,1	6,9	33	12	165	50,3
HSPA2	P54652	heat shock-relate d 70 kDa protein 2	response to stress	1,770	6,169	2,412	1,363	3,486	0,391	36,3	248,6	-60,9	0,000	0,546	0,000	0,000	0,000	70,3	5,4	33	15	151	25,8
SOD2	P04179	manganese-containing superoxide dismutase	response to stress	1,540	2,770	1,248	0,810	1,799	0,450	-19,0	79,9	-55,0	0,000	0,388	0,000	0,000	0,000	19,8	8,9	12	5	75,5	31,3
SOD2	P04179	manganese-containing superoxide dismutase	response to stress	5,280	9,976	5,094	0,965	1,889	0,511	-3,5	88,9	-48,9	0,000	0,978	0,000	0,000	0,000	22,3	7	14	5	75,4	27,8
SERPINA1	P01009	alpha-1-antitrypsin	response to stress	2,511	3,890	6,537	2,603	1,549	1,680	160,3	54,9	68,0	0,000	0,000	0,172	0,003	0,001	44,3	5,3	21	8	93,1	23,1
MB	P02144	myoglobin	transport	435,475	336,919	124,976	0,287	0,774	0,371	-71,3	-22,6	-62,9	0,000	0,000	0,001	0,000	0,000	17,2	7,9	13	8	128	55,8
ALB	P02768	serum albumin precursor	transport	28,914	57,537	120,990	4,184	1,990	2,103	318,4	99,0	110,3	0,000	0,000	0,003	0,000	0,000	71,3	6,1	34	21	223	32
TF	P02787	transferrin	transport	4,897	8,005	16,499	3,369	1,635	2,061	236,9	63,5	106,1	0,000	0,000	0,007	0,000	0,000	79,3	6,8	33	28	378	39,4
TF	P02787	transferrin	transport	1,807	6,729	11,745	6,501	3,725	1,746	550,1	272,5	74,6	0,000	0,000	0,000	0,000	0,000	74,3	6,7	27	19	237	31,8
TF	P02787	transferrin	transport	0,670	2,753	4,175	6,235	4,112	1,516	523,5	311,2	51,6	0,000	0,000	0,000	0,002	0,000	79,3	7	27	19	239	26,6

b. Other spots significantly changed vs. CTR, but not in DMD vs. BMD

Gene symbol	UniprotKB AC n°	Description	Molecular function	Av. volumes			Av.ratio			% variation			ANOVA	Tukey HSD			Kruskal-Wallis	MW [kDa]	pI	Searched peptides	Matched peptides	Mascot Score	sequence coverage [%]
				CTRL	BMD	DMD	DMD/ctrl	BMD/ctrl	DMD/BMD	DMD/ctrl	BMD/ctrl	DMD/BMD		CTRL-DMD	CTRL-BMD	BMD-DMD							
GSN	P06396	gelsolin isoform b	Structural and contractile proteins	0,178	0,616	0,535	3,014	3,466	0,870	201,4	246,6	-13,0	0,000	0,002	0,000	0,592	0,002	86	5,9	18	12	126	10,5
DES	P17661	desmin	Structural and contractile proteins (intermediate filament)	2,454	4,836	5,238	2,134	1,971	1,083	113,4	97,1	8,3	0,000	0,000	0,000	0,692	0,001	53,6	5,1	40	28	312	49,4
DES	P17661	desmin	Structural and contractile proteins (intermediate filament)	0,727	2,379	2,609	3,590	3,273	1,097	259,0	227,3	9,7	0,000	0,000	0,000	0,719	0,001	53,6	5,1	50	35	386	58,3
MYH2	Q9UKX2	myosin-2	Structural and contractile proteins (thick filaments)	0,661	1,831	2,028	3,068	2,769	1,108	206,8	176,9	10,8	0,000	0,000	0,002	0,755	0,001	80,1	6,1	21	11	109	18,1
ACTA1	P68133	alpha 1 actin precursor	Structural and contractile proteins (thin filaments)	23,402	63,976	45,015	1,924	2,734	0,704	92,4	173,4	-29,6	0,000	0,008	0,000	0,013	0,000	42,4	5,1	36	19	193	46,4
ACTA1	P68133	alpha 1 actin precursor	Structural and contractile proteins (thin filaments)	0,968	3,528	3,937	4,068	3,646	1,116	306,8	264,6	11,6	0,000	0,000	0,000	0,083	0,000	42,4	5,1	18	14	199	35,5
ACTA1	P68133	alpha 1 actin precursor	Structural and contractile proteins (thin filaments)	0,440	2,645	2,974	6,764	6,017	1,124	576,4	501,7	12,4	0,000	0,000	0,000	0,178	0,000	42,4	5,1	39	19	173	45,6
ACTA1	P68133	alpha 1 actin precursor	Structural and contractile proteins (thin filaments)	0,543	1,441	1,701	3,130	2,651	1,181	213,0	165,1	18,1	0,000	0,000	0,000	0,290	0,001	42,4	5,1	41	16	161	45,6
ACTA1	P68133	alpha 1 actin precursor	Structural and contractile proteins (thin filaments)	0,831	2,754	2,653	3,191	3,313	0,963	219,1	231,3	-3,7	0,000	0,000	0,000	0,827	0,001	42,4	5,1	29	14	143	37,7
FHL1	Q13642	four and a half LIM domains 1 variant	Structural and contractile proteins (muscle development or hypertrophy)	5,111	6,974	5,859	1,146	1,364	0,840	14,6	36,4	-16,0	0,007	0,366	0,006	0,092	0,004	35,5	10,1	30	15	207	47,6
LDB3	O75112	LIM domain binding 3 isoform 4	Structural and contractile proteins (Z-disk)	5,988	10,452	7,798	1,302	1,745	0,746	30,2	74,5	-25,4	0,002	0,229	0,001	0,044	0,008	31	9,6	21	11	167	35
MYLPF	Q96A32	myosin regulatory light chain 2, skeletal muscle isoform	Structural and contractile proteins (thick filaments)	47,656	60,915	81,564	1,712	1,278	1,339	71,2	27,8	33,9	0,000	0,000	0,183	0,016	0,003	19,2	4,7	11	9	138	44,1
ENO3	P13929	enolase 3 (beta, muscle)	Glycogen/glucose metabolism (Glycolysis)	111,828	51,172	34,233	0,306	0,458	0,669	-69,4	-54,2	-33,1	0,000	0,000	0,000	0,063	0,000	47,2	8,6	21	11	158	33,6
PKM2	P14618	pyruvate kinase 3 isoform 2	Glycogen/glucose metabolism (Glycolysis)	28,663	15,839	9,945	0,347	0,553	0,628	-65,3	-44,7	-37,2	0,000	0,000	0,000	0,056	0,000	58,5	8,7	44	21	197	36,9
GAPDH	P04406	glyceraldehyde-3-phosphate dehydrogenase	Glycogen/glucose metabolism (Glycolysis)	83,541	59,136	48,984	0,586	0,708	0,828	-41,4	-29,2	-17,2	0,001	0,000	0,011	0,342	0,003	36,2	9,1	27	12	182	44,2
IDH2	P48735	isocitrate dehydrogenase 2 (NADP+), mitochondrial precursor	TCA cycle	4,150	0,529	0,456	0,110	0,127	0,863	-89,0	-87,3	-13,7	0,000	0,000	0,001	0,995	0,004	51,3	9,6	51	24	219	50,2
CKMT2	P17540	creatine kinase S-type, mitochondrial precursor	Energy transfer	42,594	15,144	8,580	0,201	0,356	0,567	-79,9	-64,4	-43,3	0,000	0,000	0,000	0,077	0,000	48	9,3	32	17	198	49,2
UQCRC1	P31930	ubiquinol-cytochrome c reductase core protein I	Respiratory chain/ATP synthesis	3,954	2,723	1,701	0,430	0,689	0,625	-57,0	-31,1	-37,5	0,000	0,000	0,037	0,069	0,000	53,3	5,9	37	12	130	26,7
TUFM	P49411	elongation factor Tu, mitochondrial precursor (EF-Tu)	protein synthesis	4,173	1,673	1,105	0,265	0,401	0,661	-73,5	-59,9	-33,9	0,000	0,000	0,000	0,100	0,000	49,9	8,7	21	10	111	22,6
EIF2S1	Q05639	eukaryotic translation elongation factor 1 alpha 2	protein synthesis	1,186	9,327	10,015	8,443	7,864	1,074	744,3	686,4	7,4	0,000	0,000	0,000	0,869	0,001	50,8	9,7	15	8	99,4	22,7
HSP90AB1	P08238	90kDa heat shock protein	response to stress	1,844	4,428	3,581	1,942	2,401	0,809	94,2	140,1	-19,1	0,000	0,004	0,000	0,161	0,001	82,6	4,8	19	13	157	24,5
PARK7	Q99497	DJ-1 protein	response to stress	2,965	6,379	8,551	2,884	2,151	1,340	188,4	115,1	34,0	0,000	0,000	0,001	0,029	0,000	20	6,4	14	9	86,5	25,4
TRIM72	Q6ZMU5	tripartite motif-containing 72	response to stress (plasma membrane repair)	1,261	3,442	2,865	2,271	2,728	0,832	127,1	172,8	-16,8	0,000	0,001	0,000	0,278	0,002	53,6	6	18	9	139	16,4
HSPA5	P11021	endoplasmic reticulum chaperone BiP (GRP78)	response to stress	1,224	2,594	1,757	1,436	2,120	0,677	43,6	112,0	-32,3	0,002	0,305	0,002	0,049	0,005	72,2	4,9	18	10	118	20,4
HSPB1	P04792	heat shock protein 27	response to stress	1,044	1,453	1,638	1,568	1,392	1,127	56,8	39,2	12,7	0,002	0,002	0,031	0,400	0,005	22,4	9,1	23	9	133	44,7

Legend:

Significant Increment in protein abundance (Tukey HSD, p<0.01)
Significant Decrement in protein abundance (Tukey HSD, p<0.01)

Tendential Increment in protein abundance (Tukey HSD, p>0.01)
Tendential Decrement in protein abundance (Tukey HSD, p>0.01)
