# Supplementary material for: Comparative proteomic analyses of Duchenne muscular dystrophy and Becker muscular dystrophy muscles: changes contributing to preserve muscle function in Becker muscular dystrophy patients
Source: J Cachexia Sarcopenia Muscle. 2020 Jan 28;11(2):547–63. doi: 10.1002/jcsm.12527 (PMC7113522; doi:10.1002/jcsm.12527)
Supplement: Supplementary file 4 — Table S4. Changed proteins in label‐free LC‐MS/MS analysis [file JCSM-11-547-s004.pdf]

Table S4. Changed proteins in label-free LC-MS/MS analysis

a. Proteins significantly changed (ANOVA + Tukey, p<0.01) in DMD vs. BMD

Gene names	Protein IDs	Description	Molecular function	Log2 Av. Volumes (CTR, BMD, DMD); Av. Volumes (CTR, BMD, DMD); Av. Ratio (DMD/CTR, BMD/CTR, DMD/BMD); % variation (DMD/CTR, BMD/CTR, DMD/BMD); N: ANOVA p value; N: ANOVA q-value; C: ANOVA Significant; T: Significant pairs; C: Only identified by site; C: Reverse; C: Contaminant; N: Peptides; N: Razor + unique peptides; N: Unique peptides; N: Sequence coverage [%]; N: Unique + razor sequence coverage [%]; N: Unique sequence coverage [%]; N: Mol. weight [kDa]; N: PEP; N: Intensity
A2M	P01023;F8W7L3;H0YFH1	Alpha-2-macroglobulin	Extracellular matrix	[illegible]
COL6A1	P12109;A0A087X0S5	Collagen alpha-1(VI) chain	Extracellular matrix	[illegible]
COL6A2	P12110;C9JH44;H7C0M5	Collagen alpha-2(VI) chain	Extracellular matrix	[illegible]
COL6A3	P12111;E7ENL6;C9JNG9;I3L392	Collagen alpha-3(VI) chain	Extracellular matrix	[illegible]
FGG	C9JC84;P02679;C9JEU5;C9JU00;C9JPQ9	Fibrinogen gamma chain	Extracellular matrix	[illegible]
ASPN	Q9BXN1;Q5TBF2	Asporin	Extracellular matrix	[illegible]
BGN	P21810;C9JKG1	Biglycan	Extracellular matrix	[illegible]
CDH13	P55290	Cadherin-13	Extracellular matrix	[illegible]
COL1A1	P02452;I3L3H7	Collagen alpha-1(I) chain	Extracellular matrix	[illegible]
COL2A1	P02458	Collagen alpha-1(II) chain;Collagen alpha-1(II) chain;Chondrocalcin	Extracellular matrix	[illegible]
COL1A2	P08123;A0A087WTA8	Collagen alpha-2(I) chain	Extracellular matrix	[illegible]
DCN	P07585;F8VWU0;F8VUF6;F8VXZ8;F8VX58;F8VNV6;F8VSI3;F8VU58;F8VNW0	Decorin	Extracellular matrix	[illegible]
FBN1	P35555;H0YND0;P35556;A0A087WYV8;E9PHW4;F6U495	Fibrillin-1	Extracellular matrix	[illegible]
LGALS1	P09382;F8WEI7	Galectin-1	Extracellular matrix	[illegible]
LUM	P51884;CON__Q05443	Lumican	Extracellular matrix	[illegible]
OGN	P20774;Q5TBF5	Mimecan	Extracellular matrix	[illegible]
PRELP	P51888	Prolargin	Extracellular matrix	[illegible]
ACTC1	P68032	Actin, alpha cardiac muscle 1	Structural/contractile	[illegible]
MYBPC2	Q14324	Myosin-binding protein C, fast-type	Structural/contractile	[illegible]
MYH7	P12883;Q9Y2K3;Q494R4	Myosin-7	Structural/contractile	[illegible]
MYOM2	P54296;A0A0J9YVV7	Myomesin-2	Structural/contractile	[illegible]
PDLIM5	Q96HC4;H0Y8Y3;A0A0D9SFR4;H0Y929;D6RGG6;A0A0D9SFW3;D6RAA1	PDZ and LIM domain protein 5	Structural/contractile	[illegible]
VIM	P08670;B0YJC4;B0YJC5;A0A1B0GUA0;A0A1B0GTT5;Q5JVS8;A0A1B0GVG8;P41219;H7C5W5;F8W835	Vimentin	Structural/contractile	[illegible]
ACTN2	P35609;F6THM6	Alpha-actinin-2	Structural/contractile	[illegible]
MYOM1	P52179	Myomesin-1	Structural/contractile	[illegible]
FLNA	P21333;Q60FE5;Q5HY54;A0A087WWY3;H0Y5C6;H0Y5F3	Filamin-A	Structural/contractile	[illegible]
MSN	P26038	Moesin	Structural/contractile	[illegible]
MYL4	P12829;A0A1C7CYY4;I3L1K6;I3L532;I3L3U1;I3L4B1	Myosin light chain 4	Structural/contractile	[illegible]
MYL6	B7Z6Z4;G3V1V0;G8JLA2;J3KND3;P60660;F8W1R7;F8VPF3;G3V1Y7;F8W180;F8VZU9;H0YI43	Myosin light polypeptide 6	Structural/contractile	[illegible]
MYH3	P11055;A0A087X0T3;A7E2Y1	Myosin-3	Structural/contractile	[illegible]
MYH8	P13535	Myosin-8	Structural/contractile	[illegible]
MYH9	P35579	Myosin-9	Structural/contractile	[illegible]
NEB	A0A087X1N7;P20929;H0Y786;H7BZD5;H0Y3P5;H7C2Z5;H7C2B3;H7C2D0	Nebulin	Structural/contractile	[illegible]
SPTB	P11277;H0YJE6	Spectrin beta chain, erythrocyte	Structural/contractile	[illegible]
TTN	A0A0A0MTS7;Q8WZ42;A0A0A0MRA3;H7C1P9;A0A0C4DG59;H7C0U7;A0A0U1RRH3;A0A1B0GXE3;H0Y4J7;REV__H7C1P9	Titin	Structural/contractile	[illegible]
TUBA1A	Q71U36	Tubulin alpha-1A chain	Structural/contractile	[illegible]
ENO3	P13929;E5RGZ4;K7EKN2;K7EPM1;E5RG95;E5RI09;P09104;F5H0C8;F5H1C3;U3KQQ1;E5RJH5;K7EP84	Beta-enolase;Enolase	Glycolysis/gluconeogenesis	[illegible]
ALDOA	P04075;J3KPS3;H3BQN4;H3BPS8;H3BR04;H3BUH7;H3BMQ8;H3BU78;H3BR68;P05062;A0A087WXX2	Fructose-bisphosphate aldolase A;Fructose-bisphosphate aldolase	Glycolysis/gluconeogenesis	[illegible]
GPD1	P21695	Glycerol-3-phosphate dehydrogenase [NAD(+)], cytoplasmic	Glycolysis/gluconeogenesis	[illegible]
PYGM	P11217;P06737;E9PK47	Glycogen phosphorylase, muscle form	Glycolysis/gluconeogenesis	[illegible]
LDHB	P07195;A8MW50;C9J7H8;F5H793	L-lactate dehydrogenase B chain;L-lactate dehydrogenase	Glycolysis/gluconeogenesis	[illegible]
PGK1	P00558;P07205	Phosphoglycerate kinase 1	Glycolysis/gluconeogenesis	[illegible]
PKM2	P14618;B4DNK4;H3BQ34;H3BTJ2;H3BUW1;H3BT25;H3BU13;H3BQZ3;H3BN34	Pyruvate kinase isozymes M1/M2;Pyruvate kinase	Glycolysis/gluconeogenesis	[illegible]
TPI1	P60174;U3KPZ0;U3KQF3;U3KPS5	Triosephosphate isomerase	Glycolysis/gluconeogenesis	[illegible]
AGL	P35573	Glycogen debranching enzyme;4-alpha-glucanotransferase;Amylo-alpha-1,6-gluc	Glycolysis/gluconeogenesis	[illegible]
CKM	P06732	Creatine kinase M-type	TCA/Respiratory chain	[illegible]
ATP5A1	P25705;K7EK77;K7ERX7;K7EJP1;K7EQH4;K7ENJ4;K7ESA0	ATP synthase subunit alpha, mitochondrial	TCA/Respiratory chain	[illegible]
ATP5C1	P36542	ATP synthase subunit gamma, mitochondrial	TCA/Respiratory chain	[illegible]
OGDH	E9PCR7;Q02218;E9PDF2;A0A0D9SFS3;E9PFG7;C9J4G7;Q9ULD0	2-oxoglutarate dehydrogenase, mitochondrial	TCA/Respiratory chain	[illegible]
ACO2	A2A274;Q99798	Aconitate hydratase, mitochondrial	TCA/Respiratory chain	[illegible]
SLC25A4	P12235;V9GYG0;Q9H0C2	ADP/ATP translocase 1	TCA/Respiratory chain	[illegible]
AMPD1	P23109;Q01432;E9PKC5;A0A1B0GTL6	AMP deaminase 1	TCA/Respiratory chain	[illegible]
ADCK3	Q8NI60	Chaperone activity of bc1 complex-like, mitochondrial	TCA/Respiratory chain	[illegible]
UQCRC2	P22695;H3BRG4;H3BSJ9;H3BP04;H3BUI9;H3BUE4;A0A087WVZ4	Cytochrome b-c1 complex subunit 2, mitochondrial	TCA/Respiratory chain	[illegible]
CYC1	P08574	Cytochrome c1, heme protein, mitochondrial	TCA/Respiratory chain	[illegible]
MDH2	P40926;G3XAL0	Malate dehydrogenase, mitochondrial;Malate dehydrogenase	TCA/Respiratory chain	[illegible]
NDUFA4	O00483	NADH dehydrogenase [ubiquinone] 1 alpha subcomplex subunit 4	TCA/Respiratory chain	[illegible]
PDHA1	P08559;Q5JPU1;Q5JPT9;Q5JPU0;Q5JPU2;Q5JPU3	Pyruvate dehydrogenase E1 component subunit alpha, somatic form, mitochondri	TCA/Respiratory chain	[illegible]
SLC25A12	O75746;Q9UJS0;B4DGK6;H0YFB2;F8W9J0	Calcium-binding mitochondrial carrier protein Aralar1	Glutamate/glutathione metabolism	[illegible]
GOT2	P00505	Aspartate aminotransferase, mitochondrial	Glutamate/glutathione metabolism	[illegible]
GSTP1	P09211;A8MX94;A0A087X2E9;A0A087X243	Glutathione S-transferase P	Glutamate/glutathione metabolism	[illegible]
APM2	Q15847	Adipose most abundant gene transcript 2 protein	Lipid metabolism	[illegible]
ECH1	Q13011;M0R248	Delta(3,5)-Delta(2,4)-dienoyl-CoA isomerase, mitochondrial	Lipid metabolism	[illegible]
HADHA	P40939;H0YFD6	Trifunctional enzyme subunit alpha, mitochondrial;Long-chain enoyl-CoA hydratas	Lipid metabolism	[illegible]
CRAT	P43155;H0Y4Z7;A6PVN3;B7ZBP5	Carnitine O-acetyltransferase	Lipid metabolism	[illegible]
EPHX1	P07099	Epoxide hydrolase 1	Lipid metabolism	[illegible]
APOA1	P02647;F8W696;CON__P15497	Apolipoprotein A-I;Truncated apolipoprotein A-I	Lipid metabolism	[illegible]
FABP3	P05413;S4R371;S4R3A2	Fatty acid-binding protein, heart	Lipid metabolism	[illegible]
CLU	P10909;H0YC35;H0YLK8;E7ERK6;H0YAS8	Clusterin;Clusterin beta chain;Clusterin alpha chain;Clusterin	Protein/aminoacids metabolism	[illegible]
AMDHD1	Q96NU7	Probable imidazolonepropionase	Protein/aminoacids metabolism	[illegible]
LMNA	P02545;Q3BDU5;Q5TCI8;A0A0C4DGC5;H0YAB0	Prelamin-A/C;Lamin-A/C	Muscle cell development	[illegible]
HIST1H2AB	A0A0U1RRH7	Histone H2A type 1-B/E	Transcription/translation regulation	[illegible]
RPS5	M0R0R2;P46782;M0R0F0;M0QZN2	40S ribosomal protein S5;40S ribosomal protein S5, N-terminally processed	Ribosomes	[illegible]
RPSA	A0A0C4DG17;P08865;C9J9K3;F8WD59	40S ribosomal protein SA	Ribosomes	[illegible]
RPLP1	P05386	60S acidic ribosomal protein P1	Ribosomes	[illegible]
RPL30	P62888;E5RI99;A0A0C4DH44;E5RJH3;A0A0B4J213	60S ribosomal protein L30	Ribosomes	[illegible]
YWHAB	P31946	14-3-3 protein beta/alpha;14-3-3 protein beta/alpha, N-terminally processed	Signal transduction	[illegible]
YWHAQ	P27348;E9PG15	14-3-3 protein theta	Signal transduction	[illegible]
YWHAZ	E7EX29;P63104;B0AZS6;E7ESK7;H0YB80;B7Z2E6;E7EVZ2;E9PD24;E5RIR4;E5RGE1	14-3-3 protein zeta/delta	Signal transduction	[illegible]
CALM2	H0Y7A7	Calmodulin	ER/sarcoplasmic reticulum, Calcium handling	[illegible]
JPH1	Q9HDC5	Junctophilin-1	ER/sarcoplasmic reticulum, Calcium handling	[illegible]
ANXA1	P04083;Q5T3N1;Q5T3N0	Annexin A1;Annexin	Inflammation/ Immune response	[illegible]
C3	P01024;M0QYC8;M0QXZ3	Complement C3;Complement C3 beta chain;Complement C3 alpha chain;C3a ana	Inflammation/ Immune response	[illegible]
C4B	P0C0L5;A0A0G2JL54;A0A140TA29;F5GXS0	Complement C4-B;Complement C4 beta chain;Complement C4-B alpha chain;C4a	Inflammation/ Immune response	[illegible]
IGKC	A0A075B6H6;P01834	Ig kappa chain C region	Inflammation/ Immune response	[illegible]
IGLC6	A0A075B6K9	Ig lambda-6 chain C region	Inflammation/ Immune response	[illegible]
IGLL5	A0A0B4J231	Immunoglobulin lambda-like polypeptide 5	Inflammation/ Immune response	[illegible]
ANXA2	P07355	Annexin A2;Annexin	Stress response	[illegible]
ANXA5	P08758;D6RBL5;D6RBE9;E9PHT9	Annexin A5;Annexin	Blood coagulation	[illegible]
APOH	P02749;J3QRN2;J3QLI0	Beta-2-glycoprotein 1	Blood coagulation	[illegible]
HP	P00738;H0Y300;J3QR68;J3QLC9;A0A0C4DGL8;A0A087WU08;H3BS21;P00739;J3KRH2;A0A0A0MRD9;J3KTC3;H3BMJ7;J3QQI8;J3KSV1	Haptoglobin;Haptoglobin alpha chain;Haptoglobin beta chain	Transport	[illegible]
HPX	P02790;Q9BS19	Hemopexin	Transport	[illegible]
IFIT2	A0A087X279;P09913	Interferon-induced protein with tetratricopeptide repeats 2	Other proteins	[illegible]
AHNAK	Q09666	Neuroblast differentiation-associated protein AHNAK	Other proteins	[illegible]
ACAT1	P24752;E9PRQ6;H0YEL7;E9PKF3	Acetyl-CoA acetyltransferase, mitochondrial	Other proteins	[illegible]
AKR1A1	P14550	Alcohol dehydrogenase [NADP(+)]	Other proteins	[illegible]
ADH1B	P00325	Alcohol dehydrogenase 1B	Other proteins	[illegible]
AKR1B1	P15121;E9PCX2;E9PEF9;C9JRZ8	Aldose reductase	Other proteins	[illegible]
SERPINA3	P01011;G3V3A0	Alpha-1-antichymotrypsin;Alpha-1-antichymotrypsin His-Pro-less	Other proteins	[illegible]
SERPINA1	P01009	Alpha-1-antitrypsin;Short peptide from AAT	Other proteins	[illegible]
ANXA11	P50995	Annexin A11	Other proteins	[illegible]
CMBL	Q96DG6	Carboxymethylenebutenolidase homolog	Other proteins	[illegible]
PXMP2	F5GZF7	Peroxisomal membrane protein 2 (Fragment)	Other proteins	[illegible]
PEBP1	P30086	Phosphatidylethanolamine-binding protein 1;Hippocampal cholinergic neurostimula	Other proteins	[illegible]
HHATL	Q9HCP6;C9JJ25;C9JHL0	Protein-cysteine N-palmitoyltransferase HHAT-like protein	Other proteins	[illegible]
SELENBP1	Q13228;A6PVX1;F8WCR4;F2Z2W8;H0Y532;C9JVL0	Selenium-binding protein 1	Other proteins	[illegible]
VDAC1	P21796;C9JI87	Voltage-dependent anion-selective channel protein 1	Other proteins	[illegible]

b. Other proteins significantly changed vs. CTR, but not in DMD vs. BMD

Gene names	Protein IDs	Description	Molecular function	Log2 Av. Volumes (CTR, BMD, DMD); Av. Volumes (CTR, BMD, DMD); Av. Ratio (DMD/CTR, BMD/CTR, DMD/BMD); % variation (DMD/CTR, BMD/CTR, DMD/BMD); N: ANOVA p value; N: ANOVA q-value; C: ANOVA Significant; T: Significant pairs; C: Only identified by site; C: Reverse; C: Contaminant; N: Peptides; N: Razor + unique peptides; N: Unique peptides; N: Sequence coverage [%]; N: Unique + razor sequence coverage [%]; N: Unique sequence coverage [%]; N: Mol. weight [kDa]; N: PEP; N: Intensity
FGB	P02675;D6REL8;CON__P02676	Fibrinogen beta chain;Fibrinopeptide B;Fibrinogen beta chain	Extracellular matrix	[illegible]
HSPG2	P98160;A0A0U1RQT3	Basement membrane-specific heparan sulfate proteoglycan core protein;Endorep	Extracellular matrix	[illegible]
CAPNS1	A0A0C4DGQ5;K7ELJ7;P04632;K7EM73;A0A075B7C0;K7EIV0;K7EKD8;U3KQE2;K7EMQ1;K7ES78	Calpain small subunit 1	Extracellular matrix	[illegible]
LAMC1	P11047	Laminin subunit gamma-1	Extracellular matrix	[illegible]
ACTN3	Q08043;A0A087WSZ2;K7EP19;D6RH00	Alpha-actinin-3	Structural/contractile	[illegible]
FLNC	Q14315;O75369;E7EN95;F8WE98	Filamin-C	Structural/contractile	[illegible]
MYH1	P12882	Myosin-1	Structural/contractile	[illegible]
MYH4	Q9Y623	Myosin-4	Structural/contractile	[illegible]
MYOM3	Q5VTT5	Myomesin-3	Structural/contractile	[illegible]
MYOT	A0A0C4DFM5;Q9UBF9;B4DT68	Myotilin	Structural/contractile	[illegible]
TNNC1	P63316;C9JDI3	Troponin C, slow skeletal and cardiac muscles	Structural/contractile	[illegible]
TNNC2	P02585;C9J7T9	Troponin C, skeletal muscle	Structural/contractile	[illegible]
TNNT3	P45378;F8WA37;H9KVA2;C9JCA5	Troponin T, fast skeletal muscle	Structural/contractile	[illegible]
TPM1	P09493;H0YL52;Q6ZN40;H0YKP3;H0YK48;H0YNC7;H0YKX5;B7Z596;H7BYY1;F5H7S3;A0A087WTJ7;H0YL42;H0YKZ6;H0YK20;K7ELP0;H0YLS7	Tropomyosin alpha-1 chain	Structural/contractile	[illegible]
MYL1	P05976	Myosin light chain 1/3, skeletal muscle isoform	Structural/contractile	[illegible]
FHOD1	Q9Y613	FH1/FH2 domain-containing protein 1	Structural/contractile	[illegible]
MYPN	Q86TC9;A0A087WX60	Myopalladin	Structural/contractile	[illegible]
MYL2	P10916;G3V1V8;Q9BUA6	Myosin regulatory light chain 2, ventricular/cardiac muscle isoform	Structural/contractile	[illegible]
MYOZ1	Q9NP98	Myozenin-1	Structural/contractile	[illegible]
MYOZ2	Q9NPC6	Myozenin-2	Structural/contractile	[illegible]
OBSCN	A6NGQ3;Q5VST9;A0A0A0MRI8;H3BQA7;A0A1B0GTS2	Obscurin	Structural/contractile	[illegible]
PDLIM7	Q9NR12;D6RH06;H7BYK4;D6RF83;D6RAN1	PDZ and LIM domain protein 7	Structural/contractile	[illegible]
PLEC	Q15149;E9PMV1;H0YDN1;E9PKG0;E9PIA2;P58107;A0A087X1U6;A0A075B730;E9PQ28	Plectin	Structural/contractile	[illegible]
TUBB	P07437	Tubulin beta chain	Structural/contractile	[illegible]
TUBB4B	P68371	Tubulin beta-4B chain	Structural/contractile	[illegible]
TUBB8	Q3ZCM7	Tubulin beta-8 chain	Structural/contractile	[illegible]
MYO1A	Q9UBC5;G3V342	Unconventional myosin-Ia	Structural/contractile	[illegible]
WDR1	O75083;D6RD66	WD repeat-containing protein 1	Structural/contractile	[illegible]
FBP2	O00757;P09467	Fructose-1,6-bisphosphatase isozyme 2	Glycolysis/gluconeogenesis	[illegible]
GYS1	P13807;M0QYU1	Glycogen [starch] synthase, muscle	Glycolysis/gluconeogenesis	[illegible]
LDHA	P00338;F5GXY2;F5GYU2;F5GXH2;F5H6W8;F5H5J4;A0A087WUM2;F5GZQ4;P07864;F5H245;G3XAP5;F5H155;F5H5G7;F5GXU1;F5GWW2;F5GXC7;F5H8H6	L-lactate dehydrogenase A chain	Glycolysis/gluconeogenesis	[illegible]
PFKM	P08237;F8VP00;F8VZQ1;F8VNX2;F8VX13;F8VSL1;F8VZI0;F8VTQ3;F8VYK8;F8VUB8;F8VW30;F8VSF7;F8W1J8;F8VVE3;H0YHB8	6-phosphofructokinase, muscle type	Glycolysis/gluconeogenesis	[illegible]
ENO1	P06733;K7EM90	Alpha-enolase	Glycolysis/gluconeogenesis/glycogen metabolism	[illegible]
ALDOC	P09972;A8MVZ9;J3QKP5;J3KSV6;C9J8F3;K7EKH5	Fructose-bisphosphate aldolase C;Fructose-bisphosphate aldolase	Glycolysis/gluconeogenesis/glycogen metabolism	[illegible]
GPD1L	Q8N335;C9JM46;C9JFA7;C9K0P5	Glycerol-3-phosphate dehydrogenase 1-like protein	Glycolysis/gluconeogenesis/glycogen metabolism	[illegible]
GYG1	P46976;C9JQ42;G5E9W8	Glycogenin-1	Glycolysis/gluconeogenesis/glycogen metabolism	[illegible]
PGAM2	P15259;Q8N0Y7	Phosphoglycerate mutase 2	Glycolysis/gluconeogenesis/glycogen metabolism	[illegible]
IDH3A	P50213;H0YL72;H0YMU3;H0YLI6;H0YKD0	Isocitrate dehydrogenase [NAD] subunit alpha, mitochondrial	TCA/Respiratory chain	[illegible]
UQCR10	Q9UDW1	Cytochrome b-c1 complex subunit 9	TCA/Respiratory chain	[illegible]
ETFB	P38117;M0QY67	Electron transfer flavoprotein subunit beta	TCA/Respiratory chain	[illegible]
NDUFS4	O43181;H0Y9M8	NADH dehydrogenase [ubiquinone] iron-sulfur protein 4, mitochondrial	TCA/Respiratory chain	[illegible]
COX5A	H3BNX8;P20674;H3BV69;H3BRM5	Cytochrome c oxidase subunit 5A, mitochondrial	TCA/Respiratory chain	[illegible]
COX6B1	P14854	Cytochrome c oxidase subunit 6B1	TCA/Respiratory chain	[illegible]
SLC25A11	Q02978;I3L1P8	Mitochondrial 2-oxoglutarate/malate carrier protein	TCA/Respiratory chain	[illegible]
AK1	Q5T9B7;P00568;H0Y4J6;H0YID2;Q9Y6K8	Adenylate kinase isoenzyme 1	Energy transfer	[illegible]
CKB	P12277;G3V4N7;H0YJG0;G3V461	Creatine kinase B-type	Energy transfer	[illegible]
CKMT2	P17540;P12532;D6R998;D6RHV3;F8WCN3;C9JSQ1;C9JT96	Creatine kinase S-type, mitochondrial	Energy transfer	[illegible]
CAP2	P40123;E9PDI2;A0A087WZ15;A0A087X0J3;B7Z385	Adenylyl cyclase-associated protein 2;Adenylyl cyclase-associated protein	Energy transfer	[illegible]
GSTM2	F6XZQ7;P28161;E9PHN6;E9PHN7;E9PLF1;E7EWW9;A0A0A0MR85;P09488;Q03013;B9ZVX7;E9PGV1;H3BQT3	Glutathione S-transferase Mu 2	Glutamate/glutathione metabolism	[illegible]
ARL6IP5	O75915;F8WF33;C9JQU6;F8WF90	PRA1 family protein 3	Glutamate/glutathione metabolism	[illegible]
GLRX	P35754	Glutaredoxin-1	Glutamate/glutathione metabolism	[illegible]
GPT	P24298	Alanine aminotransferase 1	Glutamate/glutathione metabolism	[illegible]
GSTO1	P78417;Q5TA02;Q5TA01	Glutathione S-transferase omega-1	Glutamate/glutathione metabolism	[illegible]
HAGH	Q16775;H3BPQ4;H3BPK3;H3BQW8	Hydroxyacylglutathione hydrolase, mitochondrial	Glutamate/glutathione metabolism	[illegible]
ACAA2	P42765;A0A0B4J2A4;K7EME0;K7EJ68;K7EJB1;K7ER88	3-ketoacyl-CoA thiolase, mitochondrial	Lipid metabolism	[illegible]
CYB5R1	Q9UHQ9;H7C0R7	NADH-cytochrome b5 reductase 1	Lipid metabolism	[illegible]
PLIN4	A0A0J9YXN7;Q96Q06	Perilipin-4	Lipid metabolism	[illegible]
CYB5R3	P00387;B1AHF3	NADH-cytochrome b5 reductase 3;NADH-cytochrome b5 reductase 3 membrane-	Lipid metabolism	[illegible]
ECHS1	P30084	Enoyl-CoA hydratase, mitochondrial	Lipid metabolism	[illegible]
CAND2	O75155	Cullin-associated NEDD8-dissociated protein 2	Protein/aminoacids metabolism	[illegible]
PREP	P48147	Prolyl endopeptidase	Protein/aminoacids metabolism	[illegible]
PSMB2	P49721;A0A087WVV1	Proteasome subunit beta type-2	Protein/aminoacids metabolism	[illegible]
CSRP3	P50461	Cysteine and glycine-rich protein 3	Muscle cell development	[illegible]
KBTBD10	O60662;E9PBE3	Kelch repeat and BTB domain-containing protein 10	Muscle cell development	[illegible]
CSDA	P16989;A0A0D9SEI8;H0Y449;Q9Y2T7;P67809	DNA-binding protein A	Transcription/translation regulation	[illegible]
HIST1H2BK	U3KQK0	Histone H2B type 1-K	Transcription/translation regulation	[illegible]
ZEB1	P37275	Zinc finger E-box-binding homeobox 1	Transcription/translation regulation	[illegible]
EEF1A2	Q05639	Elongation factor 1-alpha 2	Ribosomes	[illegible]
EEF1D	E9PRY8;P29692;A0A087X1X7;E9PK01;E9PQZ1;H0YCK7;E9PI39;E9PQ49;E9PL12;E9PPR1;H0YE72;E9PIZ1;E9PMW7;H0YE58	Elongation factor 1-delta	Ribosomes	[illegible]
EEF1G	P26641	Elongation factor 1-gamma	Ribosomes	[illegible]
CD36	P16671;E7EU05;E9PLT1	Platelet glycoprotein 4	Signal transduction	[illegible]
PACSIN3	Q9UKS6;A0A0C4DGG1;E9PIY1;E9PNM9;E9PJ75;E9PJ33;H0YDM6	Protein kinase C and casein kinase substrate in neurons protein 3	Signal transduction	[illegible]
YWHAE	P62258;K7EM20;K7EIT4;I3L3T1;B4DJF2;P31947;I3L0W5;A0A0J9YWE8;Q4VY19;A0A0J9YWZ2;Q4VY20	14-3-3 protein epsilon	Signal transduction	[illegible]
ATP2A1	O14983;H3BTW4;H3BVB2;H3BTF1;H3BUT9	Sarcoplasmic/endoplasmic reticulum calcium ATPase 1	ER/sarcoplasmic reticulum, Calcium handling	[illegible]
CASQ2	O14958	Calsequestrin-2	ER/sarcoplasmic reticulum, Calcium handling	[illegible]
RTN4	Q9NQC3;F8W914;H7C106;A0A0U1RQR6	Reticulon-4	ER/sarcoplasmic reticulum, Calcium handling	[illegible]
CASQ1	P31415;C9JAC8	Calsequestrin-1	ER/sarcoplasmic reticulum, Calcium handling	[illegible]
DYSF	O75923;Q9NZM1	Dysferlin	ER/sarcoplasmic reticulum, Calcium handling	[illegible]
HRC	P23327;M0QZ43	Sarcoplasmic reticulum histidine-rich calcium-binding protein	ER/sarcoplasmic reticulum, Calcium handling	[illegible]
NSFL1C	Q9UNZ2;F2Z2K0;R4GNE6;R4GMY2;G3V4V8	NSFL1 cofactor p47	ER/sarcoplasmic reticulum, Calcium handling	[illegible]
PDIA3	P30101	Protein disulfide-isomerase A3	ER/sarcoplasmic reticulum, Calcium handling	[illegible]
SRL	Q86TD4;B4DYT9	Sarcalumenin	ER/sarcoplasmic reticulum, Calcium handling	[illegible]
VCP	P55072	Transitional endoplasmic reticulum ATPase	ER/sarcoplasmic reticulum, Calcium handling	[illegible]
IGHM	A0A1B0GUU9;A0A075B6N9;A0A087WYJ9;P01871;P04220	Ig mu chain C region;Ig mu heavy chain disease protein	Inflammation/ Immune response	[illegible]
MIF	P14174	Macrophage migration inhibitory factor	Inflammation/ Immune response	[illegible]
HSPA9	P38646;H0Y8S0	Stress-70 protein, mitochondrial	Stress response	[illegible]
HSPB6	O14558;K7EP04	Heat shock protein beta-6	Stress response	[illegible]
HSPA1B	A0A0G2JIW1;P0DMV8;P0DMV9;V9GZ37	Heat shock 70 kDa protein 1B	Stress response	[illegible]
HSPB2	A8KAH6;Q16082;E9PN16	Heat shock protein beta-2	Stress response	[illegible]
HSPD1	P10809;E7ESH4;E7EXB4;C9JL25;C9JCQ4;C9JL19	60 kDa heat shock protein, mitochondrial	Stress response	[illegible]
HSPE1	S4R3N1;P61604;B8ZZL8	10 kDa heat shock protein, mitochondrial	Stress response	[illegible]
PRDX5	P30044	Peroxiredoxin-5, mitochondrial	Stress response	[illegible]
SOD1	P00441;H7BYH4	Superoxide dismutase [Cu-Zn]	Stress response	[illegible]
HBG2	P69892	Hemoglobin subunit gamma-2	Transport proteins	[illegible]
TF	P02787;H7C5E8;C9JVG0;F8WCI6;F8WEK9;C9JB55;F8WC57	Serotransferrin	Transport proteins	[illegible]

HBB	P68871;F8W6P5;A0A0J9YWK4	Hemoglobin subunit beta;LVV-hemorphin-7	Transport	30,06458	30,39754	31,01119	1122898204	1414397026	2164204976	1,927	1,260	1,530	92,7	26,0	53,0	0,0005293	0,00113043	+	DMD_CTR			14	14	8	93,9	93,9	67,3	15,998	0	11813000000
HBD	P02042;E9PFT6;E9PEW8;C9JRG0;A8MUF7	Hemoglobin subunit delta	Transport	25,05796	25,78472	26,22319	34929919,93	57806163,88	78336970,46	2,243	1,655	1,355	124,3	65,5	35,5	0,0027911	0,00195862	+	DMD_CTR			12	6	6	91,2	53,1	53,1	16,055	0	369710000
HBE1	P02100	Hemoglobin subunit epsilon	Transport	19,02071	19,81774	20,87065	531868,4742	924132,3775	1917307,093	3,605	1,738	2,075	260,5	73,8	107,5	0,0005483	0,00110638	+	DMD_CTR			3	2	2	20,4	13,6	13,6	16,203	7,3006E-20	7479000
ORM1	P02763;P19652	Alpha-1-acid glycoprotein 1	Transport	17,23304	17,93168	19,02221	154050,09	250019,3102	532421,7564	3,456	1,623	2,130	245,6	62,3	113,0	0,0012469	0,00087395	+	DMD_CTR			3	3	3	15,4	15,4	15,4	23,511	7,9247E-06	2051900
TTR	P02766;A0A087WV45;A0A087WT59	Transthyretin	Transport	20,21845	19,81674	21,73764	1220000,239	923492,0397	3496887,615	2,866	0,757	3,787	186,6	-24,3	278,7	0,0117342	0,00533333	+	DMD_CTR			2	2	2	30,6	30,6	30,6	15,887	1,7245E-20	12378000
EHD2	Q9NZN4;A0A024R571;Q9NZN3;Q9H4M9;C9JC03;C9IZH1;C9JIJ3	EH domain-containing protein 2	Caveolae	19,64379	19,87092	20,8247	819162,4166	958832,9752	1857202,89	2,267	1,171	1,937	126,7	17,1	93,7	0,0063344	0,0034152	+	DMD_CTR			4	4	4	9,9	9,9	9,9	61,161	7,1079E-13	8034300
PTRF	Q6NZI2	Polymerase I and transcript release factor	Caveolae	20,89645	21,25851	22,20795	1951903,096	2508702,561	4844613,022	2,482	1,285	1,931	148,2	28,5	93,1	4,195E-05	0	+	DMD_CTR			4	4	4	20,8	20,8	20,8	43,476	7,7256E-107	22045000
C3orf49	A0A087WV64;Q96BT1	Putative uncharacterized protein C3orf49	Other proteins	21,65403	21,87848	21,20943	3299989,492	3855482,53	2424792,728	0,735	1,168	0,629	-26,5	16,8	-37,1	0,0176602	0,00589091	+	CTR_DMD	BMD_CTR		1	1	1	2,7	2,7	2,7	33,491	0,0019146	22308000
CA2	P00918;E5RK37;E5RID5	Carbonic anhydrase 2	Other proteins	20,62943	19,9655	19,74586	1622098,503	1023798,24	879217,1487	0,542	0,631	0,859	-45,8	-36,9	-14,1	0,0295415	0,00845902	+	CTR_DMD	CTR_BMD		4	4	4	23,8	23,8	23,8	29,246	2,5423E-16	10592000
HIST1H4A	P62805	Histone H4	Other proteins	21,32619	24,08877	24,6329	2629195,518	17841950,44	26016075,26	9,895	6,786	1,458	889,5	578,6	45,8	4,67E-06	0	+	DMD_CTR	BMD_CTR		6	6	6	57,3	57,3	57,3	11,367	1,8822E-17	89581000
IGHG1	A0A0A0MS08;P01857;A0A0A0MS07	Ig gamma-1 chain C region	Other proteins	24,18056	25,17486	26,15933	19014016,74	37878083,46	74945057,38	3,942	1,992	1,979	294,2	99,2	97,9	1,666E-05	0	+	DMD_CTR	BMD_CTR		11	11	6	40,4	40,4	22,1	43,911	1,6252E-186	297580000
METTL7A	H0YI09;Q9H8H3;F8VQX6	Methyltransferase-like protein 7A	Other proteins	19,90707	20,64373	20,98503	983162,2522	1638256,699	2075503,595	2,111	1,666	1,267	111,1	66,6	26,7	0,0041807	0,00297436	+	DMD_CTR	BMD_CTR		2	2	2	11,1	11,1	11,1	28,188	2,2986E-13	10238000
MCM3AP	O60318;A0A0A0MSZ7	80 kDa MCM3-associated protein	Other proteins	19,72858	21,23922	19,48267	868749,0683	2475382,433	732601,9421	0,843	2,849	0,296	-15,7	184,9	-70,4	0,0087671	0,00413333	+		BMD_CTR	+	1	1	1	1,4	1,4	1,4	218,4	0,033547	7635200
NAP1L1	H0YHC3	Nucleosome assembly protein 1-like 1 (Fragment)	Other proteins	19,56536	18,05925	18,54272	775818,6866	273134,1159	381869,4548	0,492	0,352	1,398	-50,8	-64,8	39,8	0,0979767	0,027175	+		CTR_BMD	+	1	1	1	8,6	8,6	8,6	23,417	0,023383	4528800
VDAC3	Q9Y277;E5RHZ6;E5RJN6;E5RFP6;E5RK27;E5RHE1	Voltage-dependent anion-selective channel protein 3	Other proteins	23,39558	23,80863	23,5798	11034974,81	14693044,22	12537966,11	1,136	1,331	0,853	13,6	33,1	-14,7	0,0142819	0,00547573	+		BMD_CTR		8	8	8	45,9	45,9	45,9	30,658	6,4438E-222	98059000
AIFM1	O95831	Apoptosis-inducing factor 1, mitochondrial	Other proteins	20,62212	19,26531	18,92088	1613900,27	630138,748	496309,3127	0,308	0,390	0,788	-69,2	-61,0	-21,2	0,0059262	0,00326627	+	CTR_DMD			4	4	4	8,3	8,3	8,3	66,9	4,9841E-17	8267400
APOBEC2	Q9Y235	Probable C->U-editing enzyme APOBEC-2	Other proteins	23,09276	22,83675	22,60601	8945681,728	7491119,99	6383914,947	0,714	0,837	0,852	-28,6	-16,3	-14,8	0,0027953	0,00194521	+	CTR_DMD			7	7	7	48,7	48,7	48,7	25,703	6,7886E-191	62926000
CA1	P00915;E5RHP7;E5RH81;E5RFE7;H0YBE2;E5RG43;E5RIF9;E5RJI8;E5RG81;E5RFL2;E5RGU8;E5RJF6;E5RII2	Carbonic anhydrase 1	Other proteins	22,87951	23,41326	23,33593	7716472,203	11171038,7	10588023,75	1,372	1,448	0,948	37,2	44,8	-5,2	0,0239216	0,0073133	+	DMD_CTR			7	7	7	33,7	33,7	33,7	28,87	4,5158E-60	75095000
CRIP2	P52943;H0YFA4	Cysteine-rich protein 2	Other proteins	16,54234	17,10729	18,48978	95442,22131	141191,1367	368110,6592	3,857	1,479	2,607	285,7	47,9	160,7	0,0039524	0,00303268	+	DMD_CTR			1	1	1	15,4	15,4	15,4	22,492	6,1138E-33	1146700
HINT1	P49773;D6RD60;D6RE99;D6REP8	Histidine triad nucleotide-binding protein 1	Other proteins	18,67497	16,657	15,44286	418529,5612	103337,1876	44541,42209	0,106	0,247	0,431	-89,4	-75,3	-56,9	0,0032858	0,00235135	+	CTR_DMD			1	1	1	15,9	15,9	15,9	13,802	7,6898E-17	2399100
NR2C1	F8W1Q1	Nuclear receptor subfamily 2 group C member 1 (Fragment)	Other proteins	18,32316	19,07466	18,91887	327959,9227	552134,4139	495618,3232	1,511	1,684	0,898	51,1	68,4	-10,2	0,0363463	0,00985938	+	DMD_CTR		+	1	1	1	100	100	100	1,8852	0,0092874	2850600
PADI2	Q9Y2J8	Protein-arginine deiminase type-2	Other proteins	20,96466	21,53488	21,63954	2046404,626	3038399,2	3267011,268	1,596	1,485	1,075	59,6	48,5	7,5	0,0145444	0,00556522	+	DMD_CTR			9	9	9	18,6	18,6	18,6	75,563	1,9946E-71	21978000
PPIA	P62937;C9J5S7;F8WE65;A0A075B759;A0A075B767;A0A0B4J2A2;A0A0H2UH34;F5H284;P0DN26;P0DN37;Q9Y536	Peptidyl-prolyl cis-trans isomerase A;Peptidyl-prolyl cis-trans isomerase	Other proteins	21,39994	21,67116	22,66001	2767093,572	3339405,828	6627392,806	2,395	1,207	1,985	139,5	20,7	98,5	0,0002913	0,0013	+	DMD_CTR			6	6	6	38,2	38,2	38,2	18,012	1,1893E-27	24502000
SLC4A1	P02730;A0A0A0MS98	Band 3 anion transport protein	Other proteins	19,92351	20,53839	21,33859	994429,7968	1522900,231	2651890,913	2,667	1,531	1,741	166,7	53,1	74,1	0,0089173	0,00424044	+	DMD_CTR			6	6	6	9,8	9,8	9,8	101,79	1,2537E-16	12413000

Legend:

Significant Increment in protein abundance (Tukey HSD, p<0.01)

Significant Decrement in protein abundance (Tukey HSD, p<0.01)

Tendential Increment in protein abundance (Tukey HSD, p>0.01)

Tendential Decrement in protein abundance (Tukey HSD, p>0.01)
