# Supplementary material for: Comparative proteomic analyses of Duchenne muscular dystrophy and Becker muscular dystrophy muscles: changes contributing to preserve muscle function in Becker muscular dystrophy patients
Source: J Cachexia Sarcopenia Muscle. 2020 Jan 28;11(2):547–63. doi: 10.1002/jcsm.12527 (PMC7113522; doi:10.1002/jcsm.12527)

Figure S5. List of annotated spectra for MALDI–ToF identified proteins

A representative MALDI-ToF PMF spectrum of MYL3

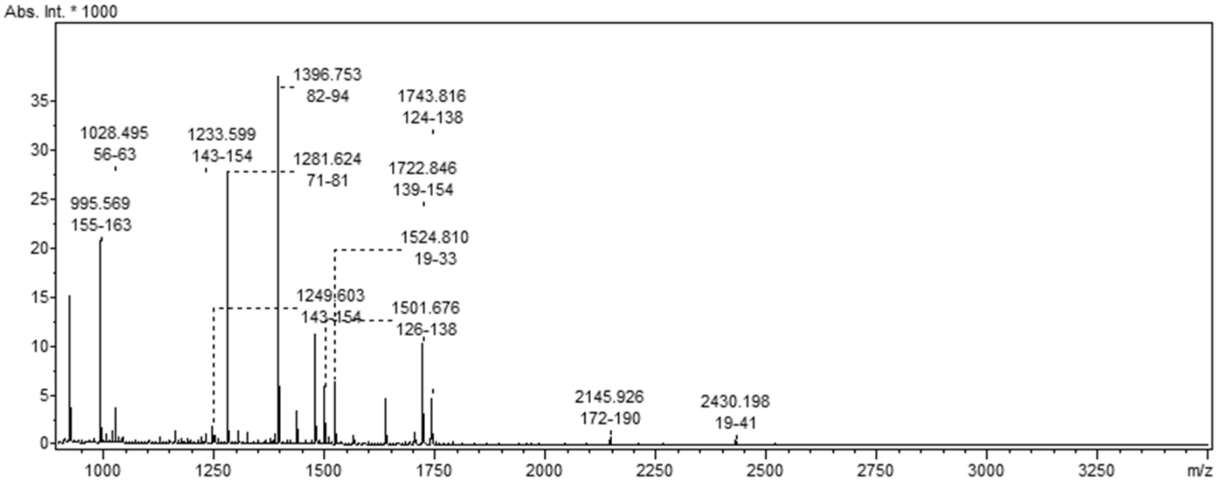

A representative MALDI-ToF PMF spectrum of TNNT3

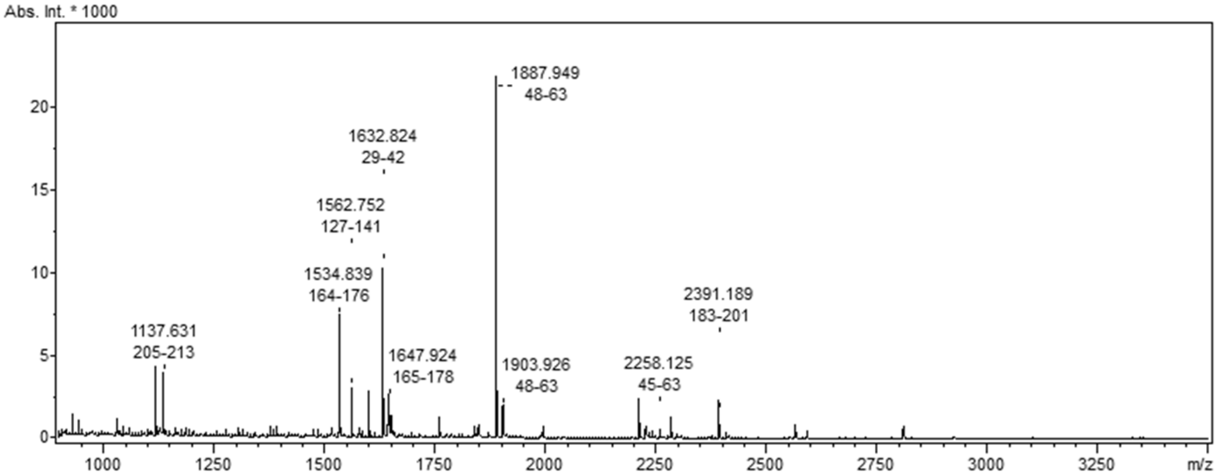

A representative MALDI-ToF PMF spectrum of TNNI2

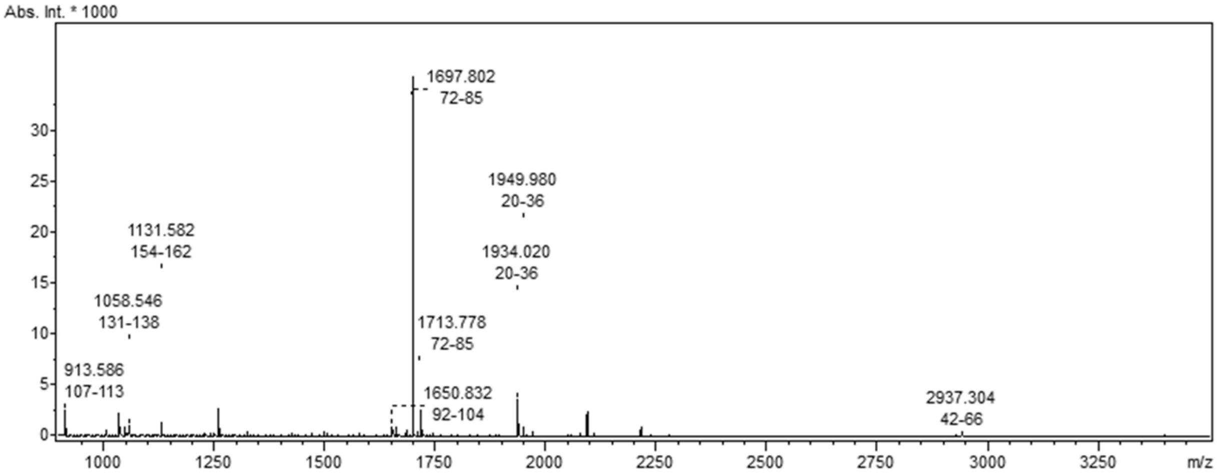

A representative MALDI-ToF PMF spectrum of PYGM

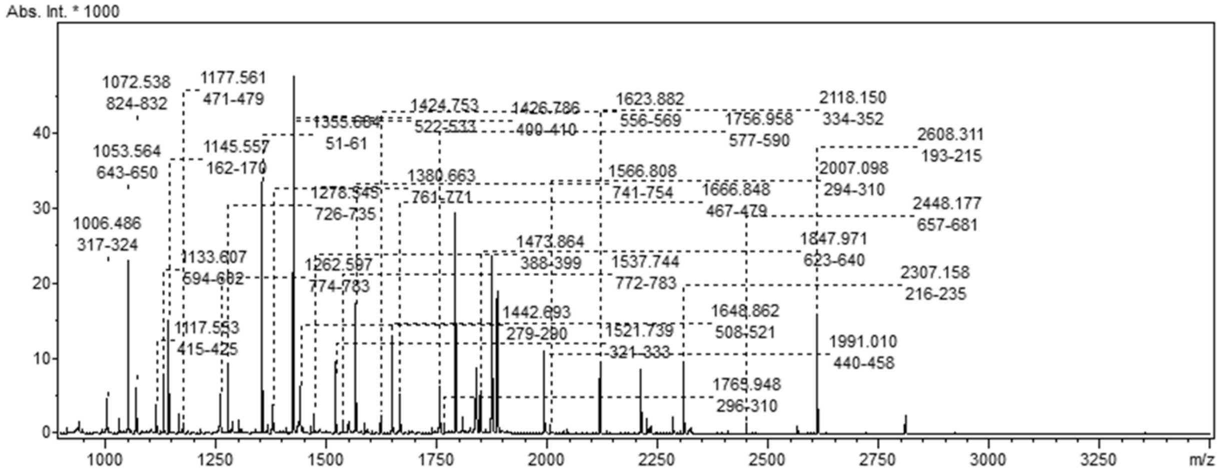

A representative MALDI-ToF PMF spectrum of ALDOA, proteoform a

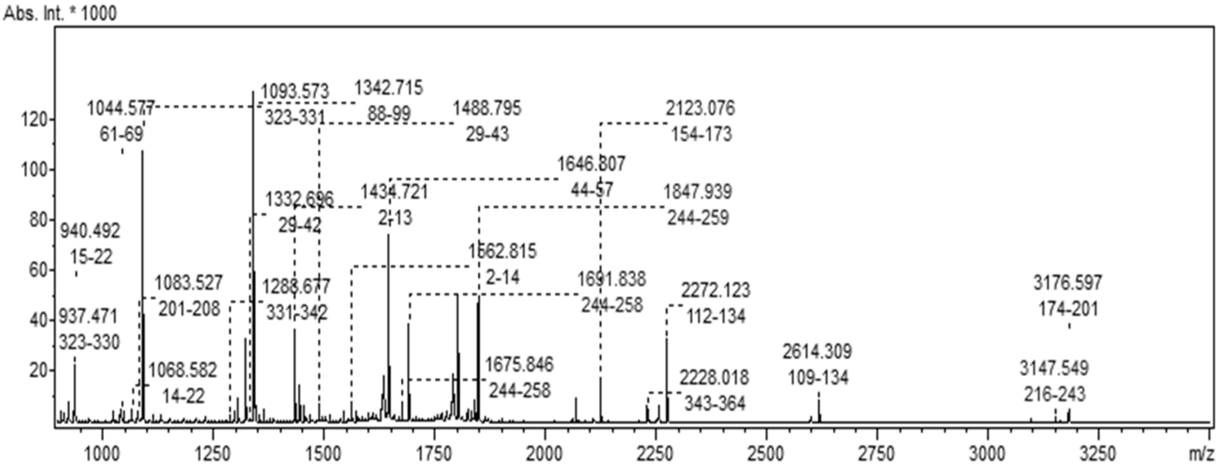

A representative MALDI-ToF PMF spectrum of ALDOA, proteoform b

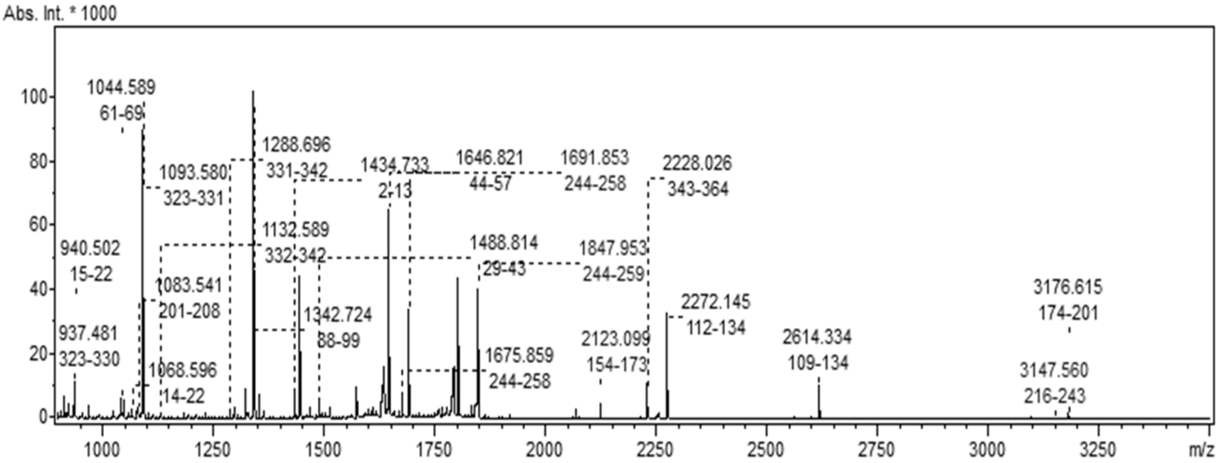

A representative MALDI-ToF PMF spectrum of PGK1

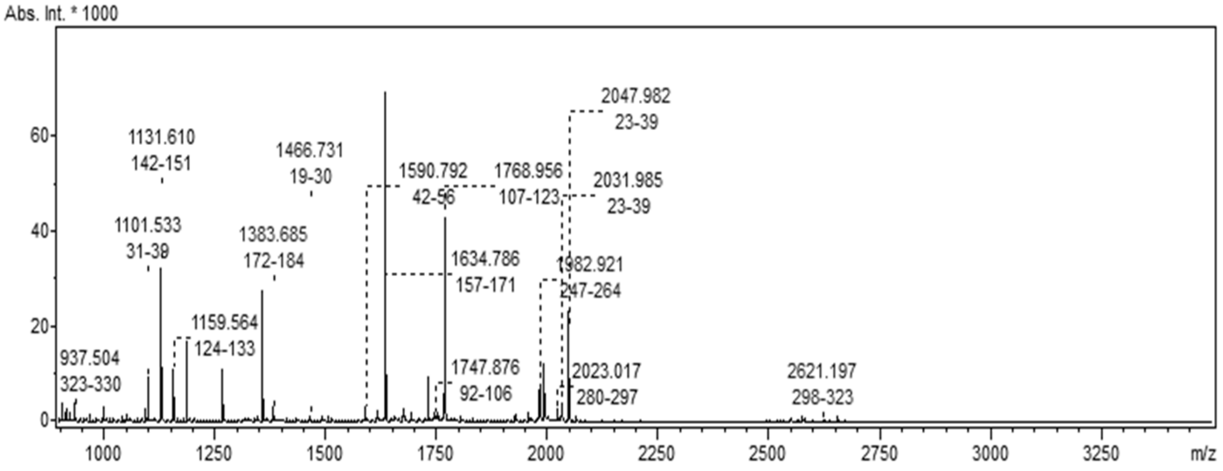

A representative MALDI-ToF PMF spectrum of TPI1, proteoform a

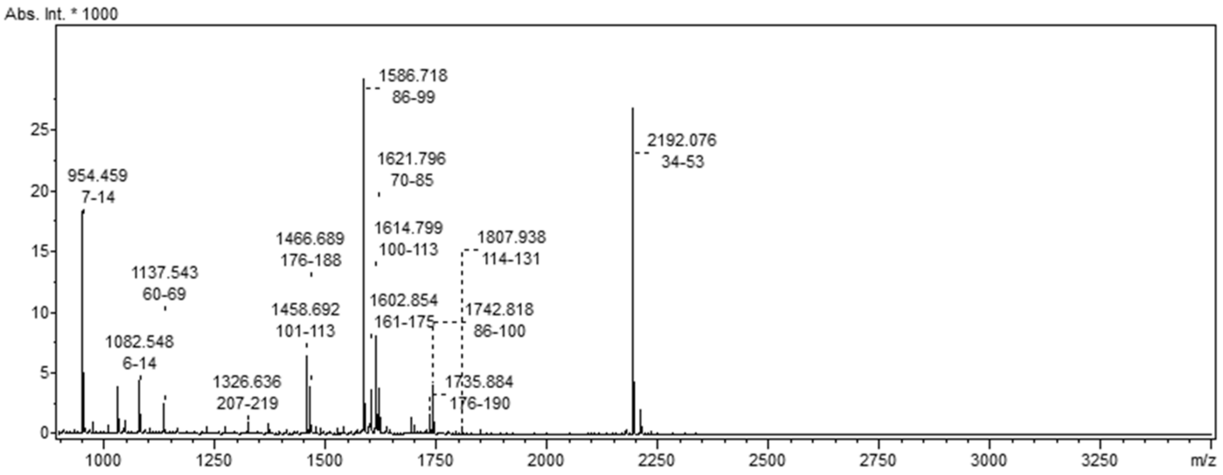

A representative MALDI-ToF PMF spectrum of TPI1, proteoform b

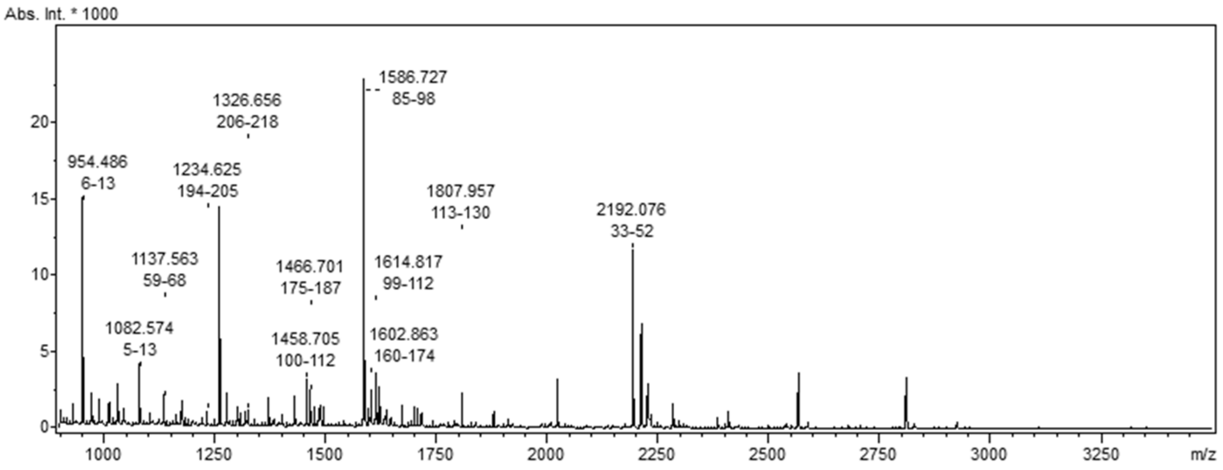

A representative MALDI-ToF PMF spectrum of PGM1

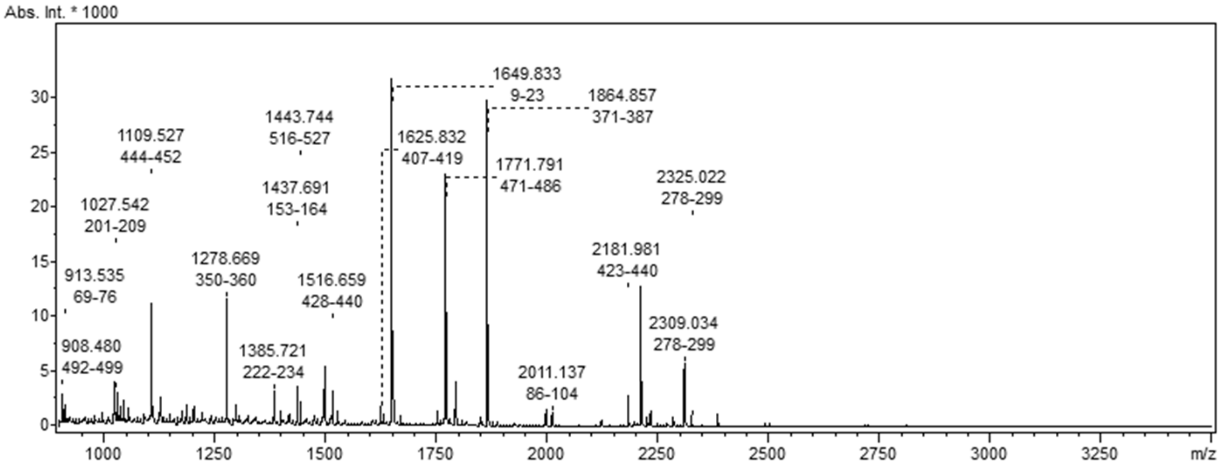

A representative MALDI-ToF PMF spectrum of PGAM2

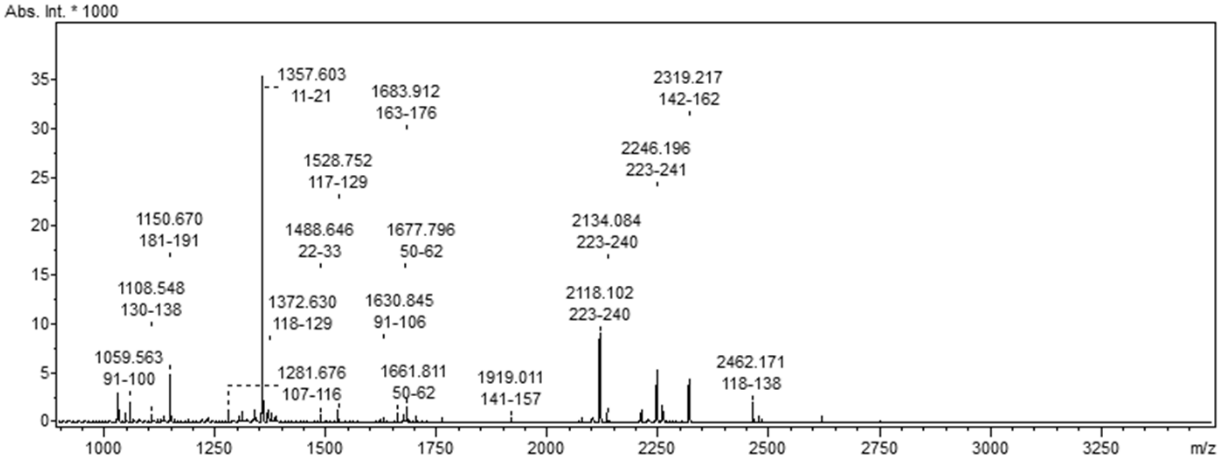

A representative MALDI-ToF PMF spectrum of MDH1

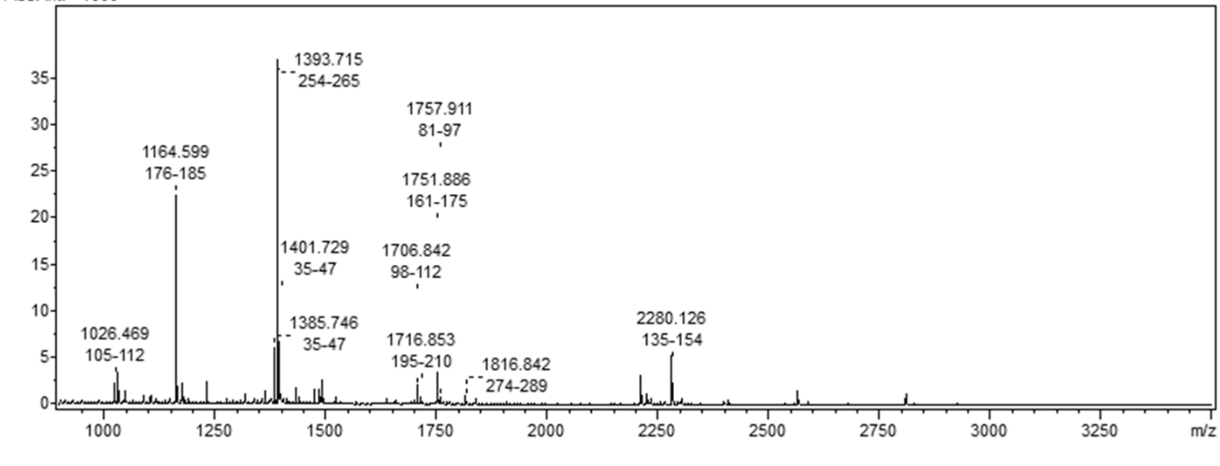

A representative MALDI-ToF PMF spectrum of SDHA

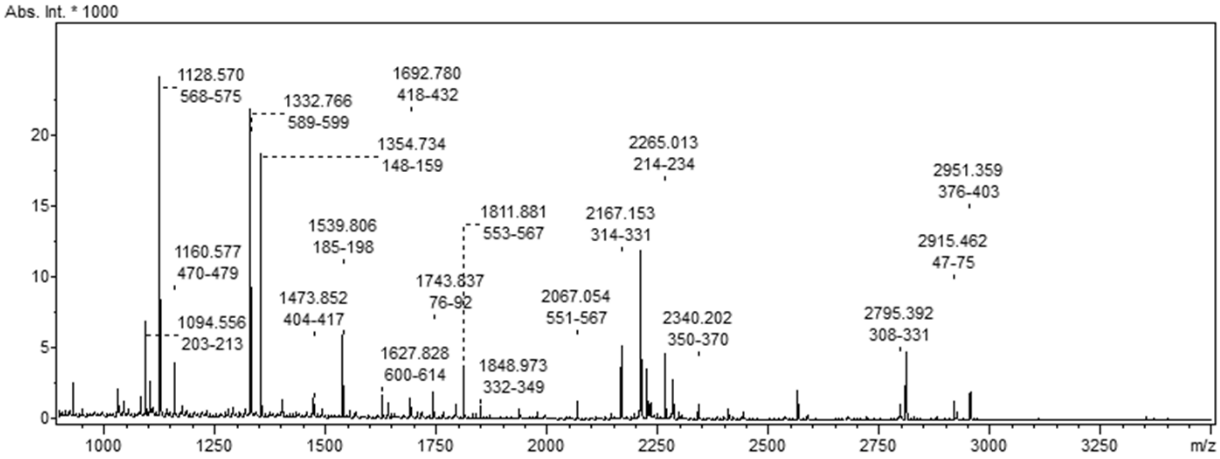

A representative MALDI-ToF PMF spectrum of SUCLA2

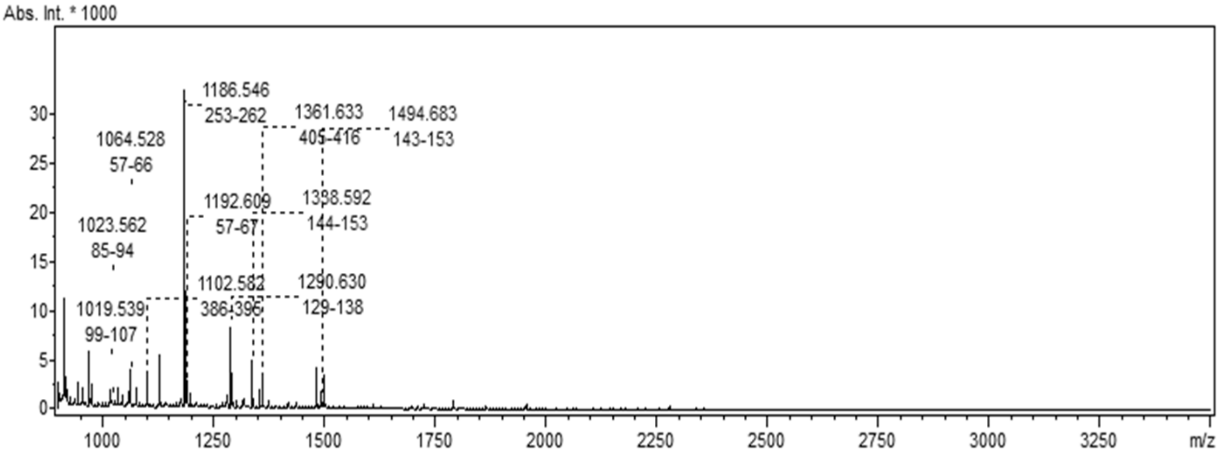

A representative MALDI-ToF PMF spectrum of ATP5B

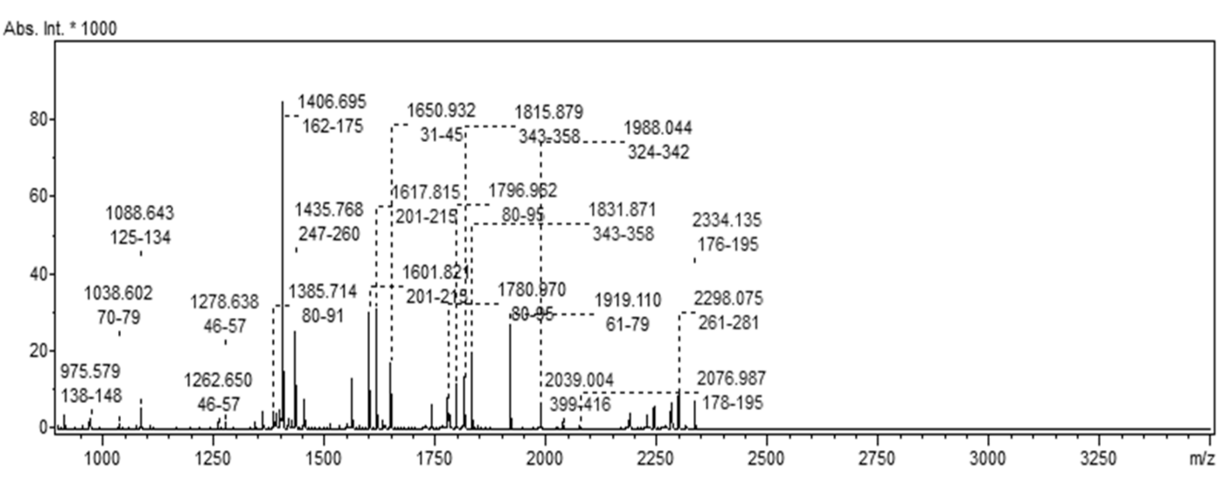

A representative MALDI-ToF PMF spectrum of GOT1

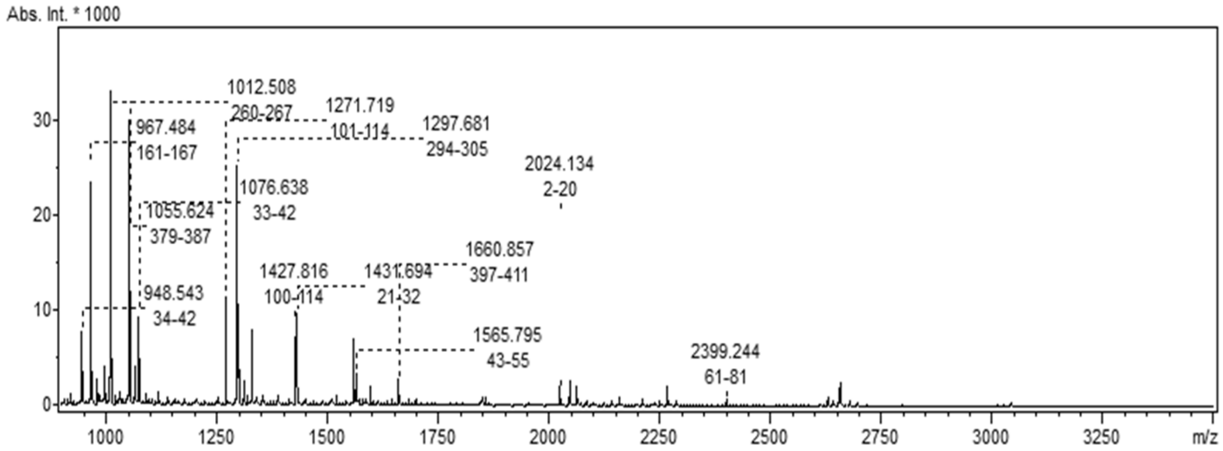

A representative MALDI-ToF PMF spectrum of GOT2

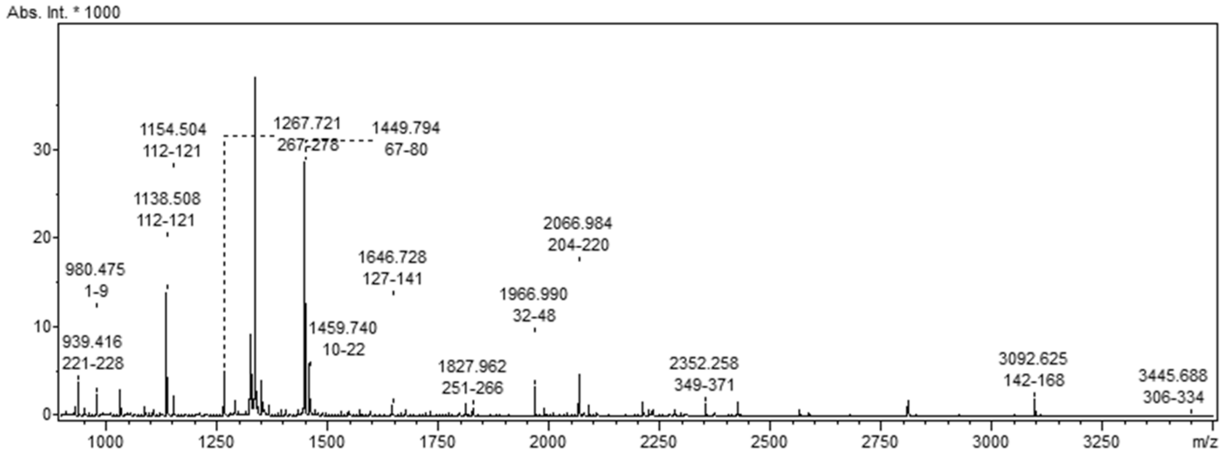

A representative MALDI-ToF PMF spectrum of CA3

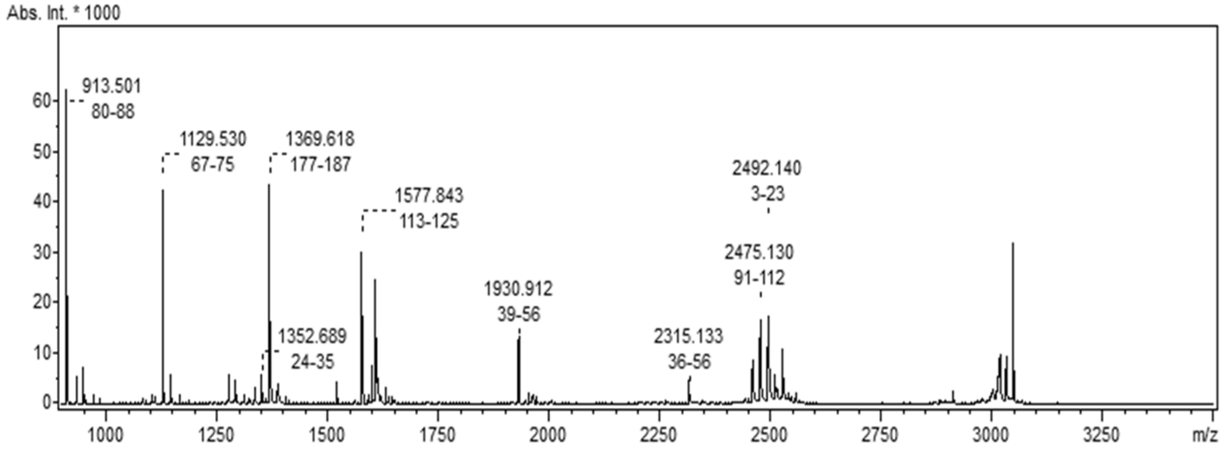

A representative MALDI-ToF PMF spectrum of PRDX6

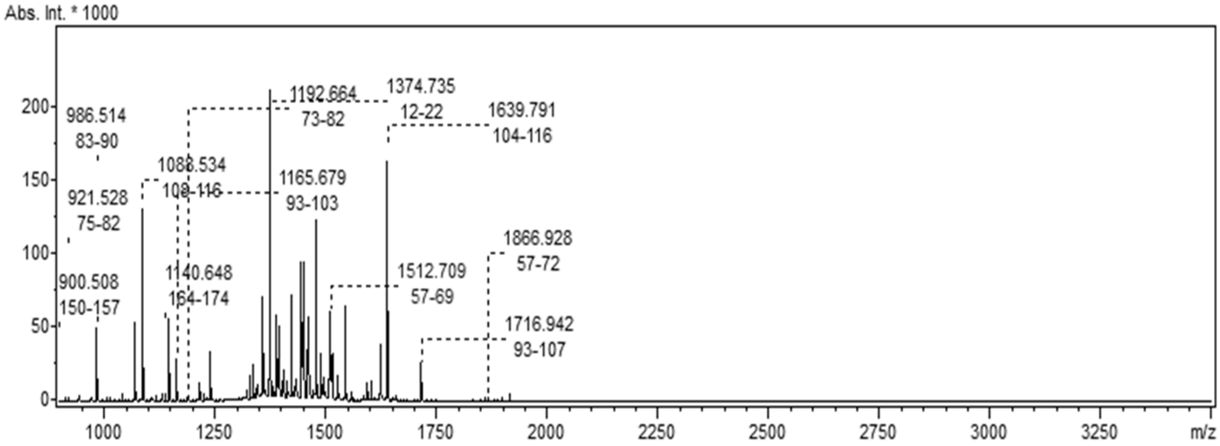

A representative MALDI-ToF PMF spectrum of CRYAB

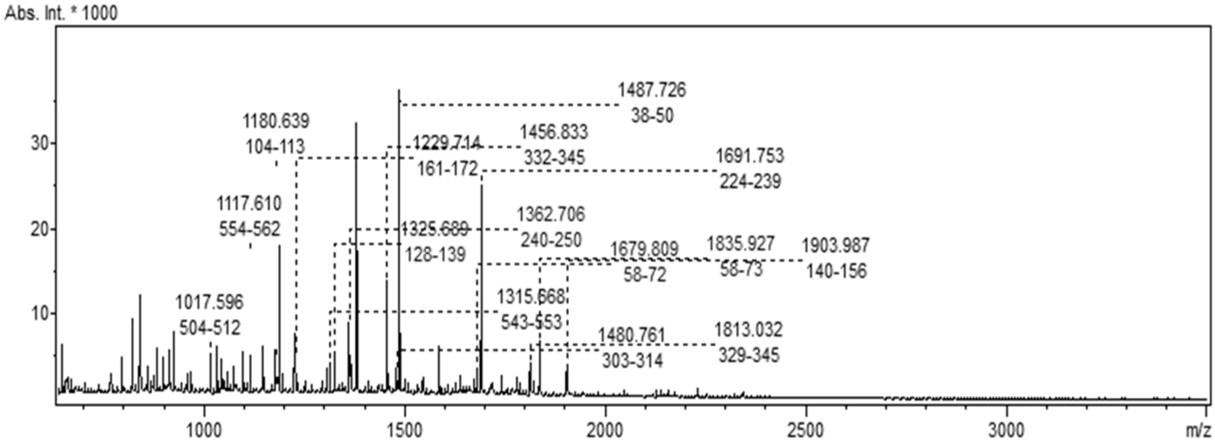

A representative MALDI-ToF PMF spectrum of HSPA2

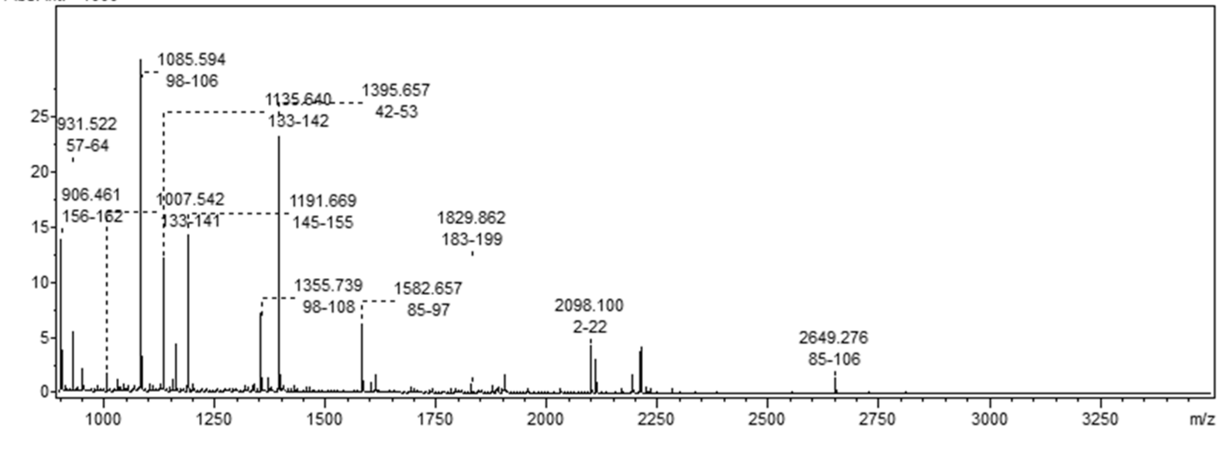

A representative MALDI-ToF PMF spectrum of SOD2, proteoform a

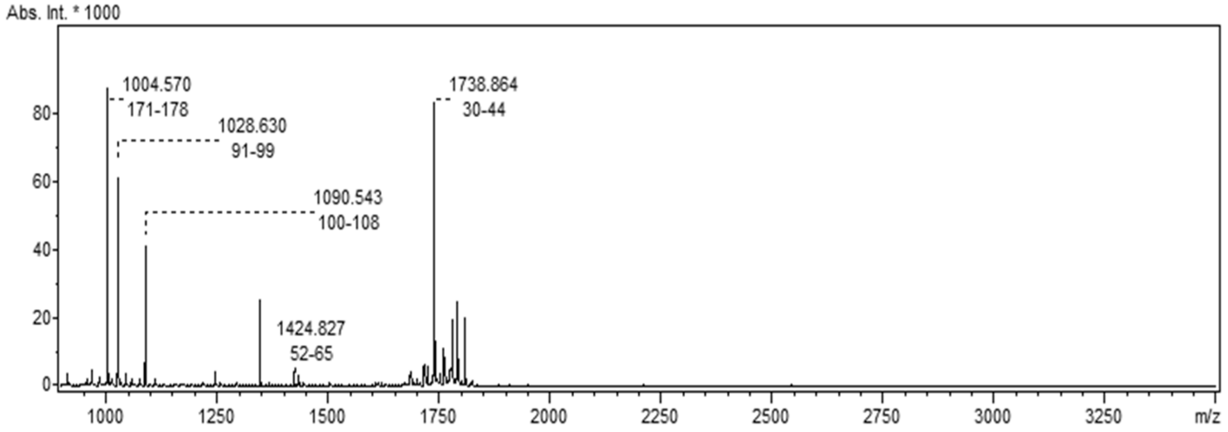

A representative MALDI-ToF PMF spectrum of SOD2, proteoform b

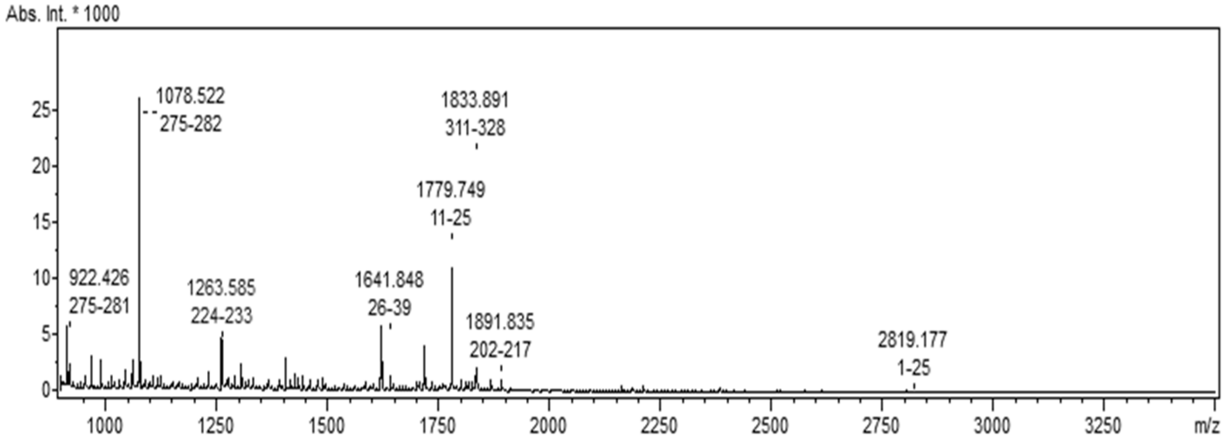

A representative MALDI-ToF PMF spectrum of SERPINA1

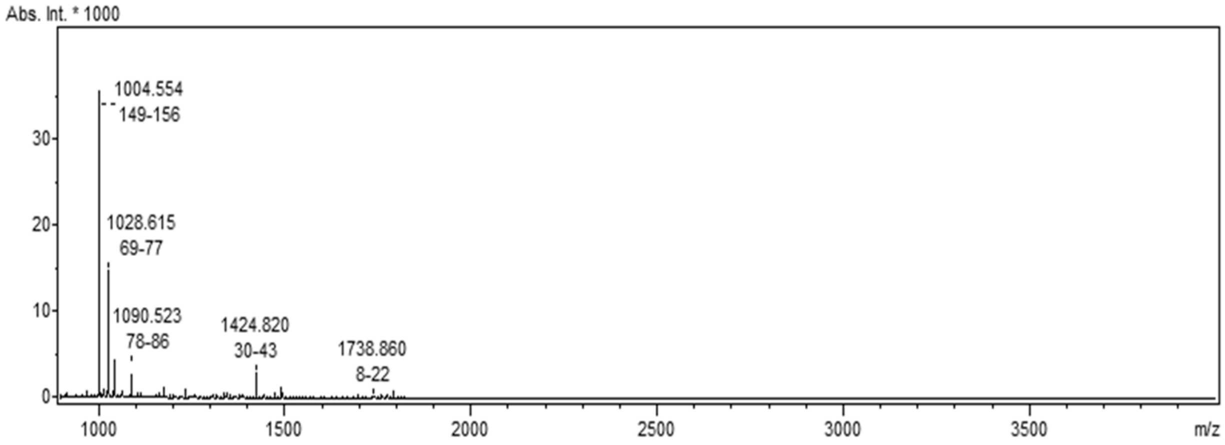

A representative MALDI-ToF PMF spectrum of MB

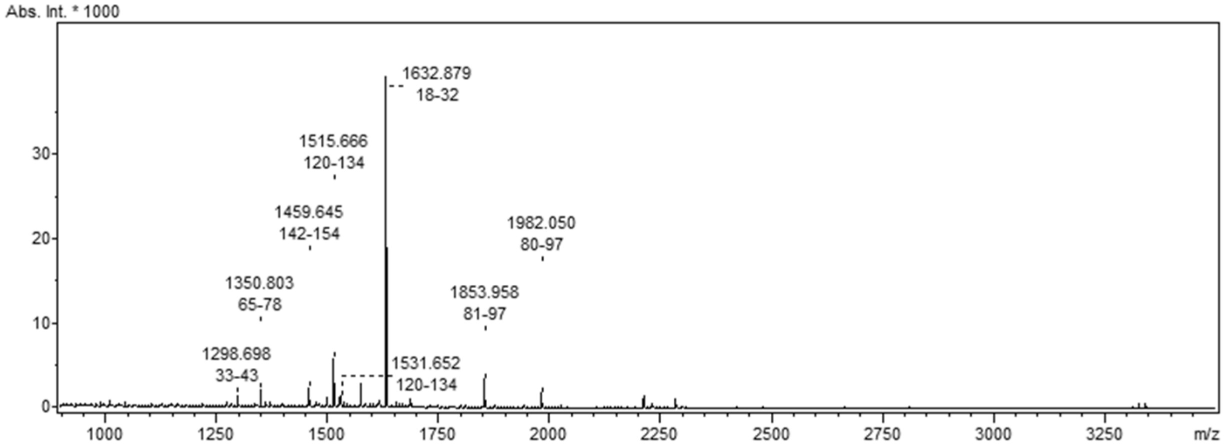

A representative MALDI-ToF PMF spectrum of ALB

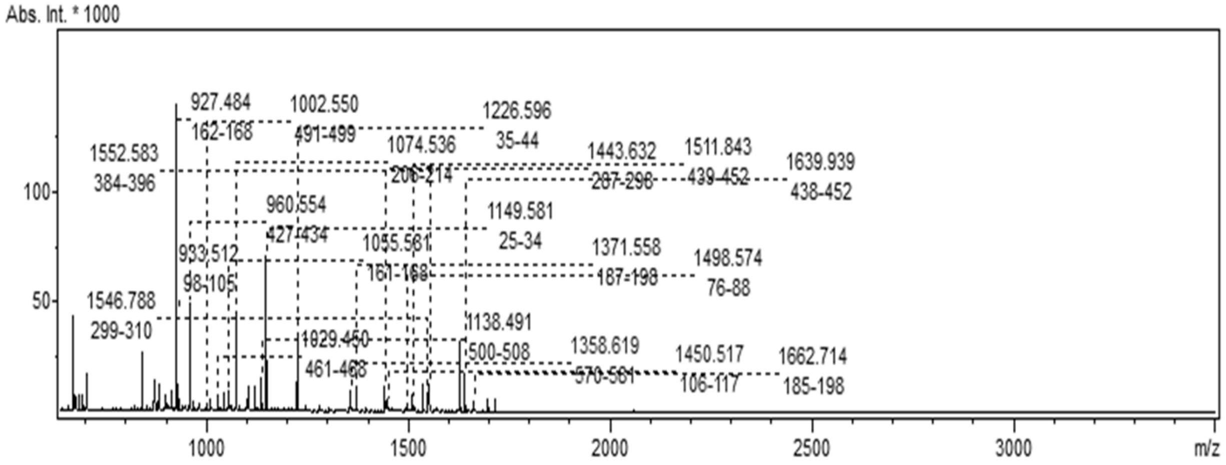

A representative MALDI-ToF PMF spectrum of TF, proteoform a

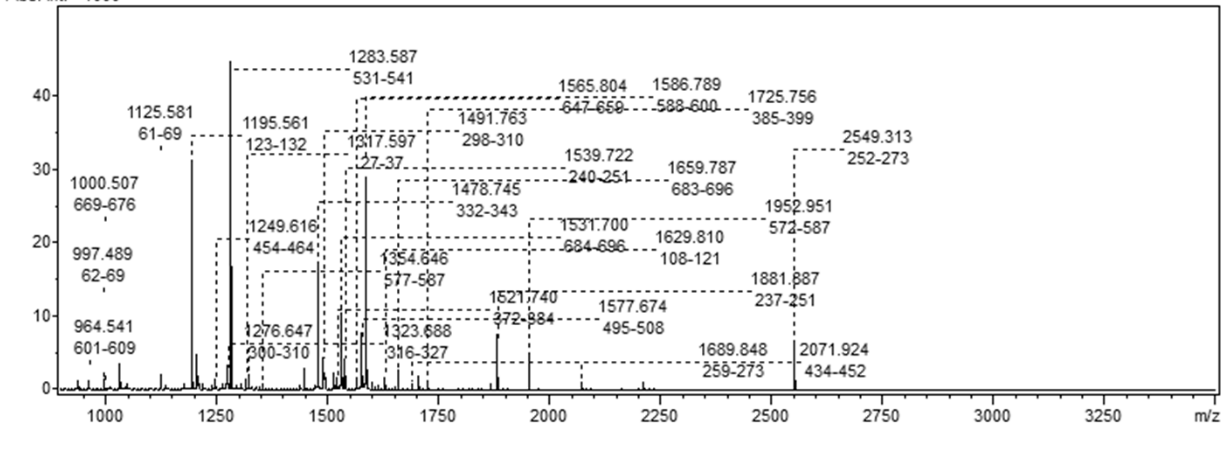

A representative MALDI-ToF PMF spectrum of TF, proteoform b

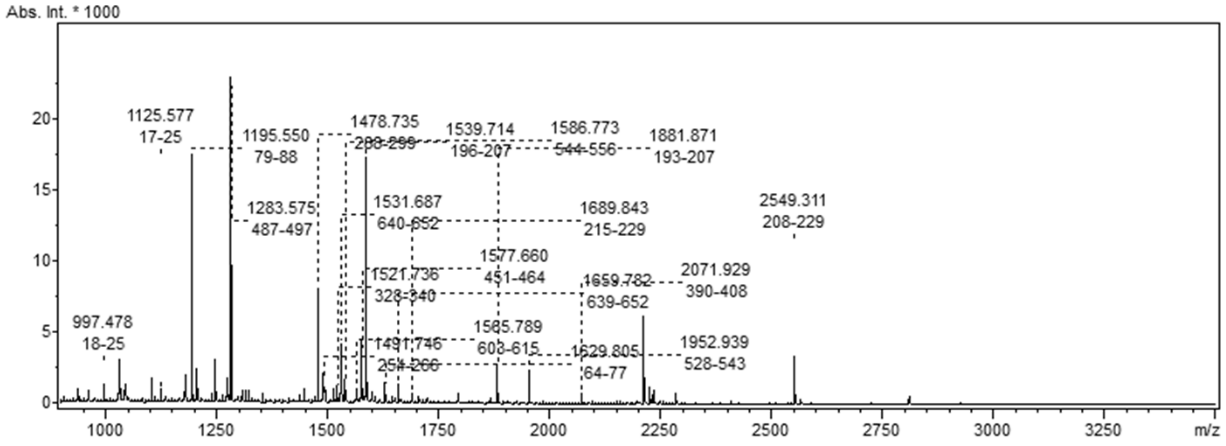

A representative MALDI-ToF PMF spectrum of TF, proteoform c

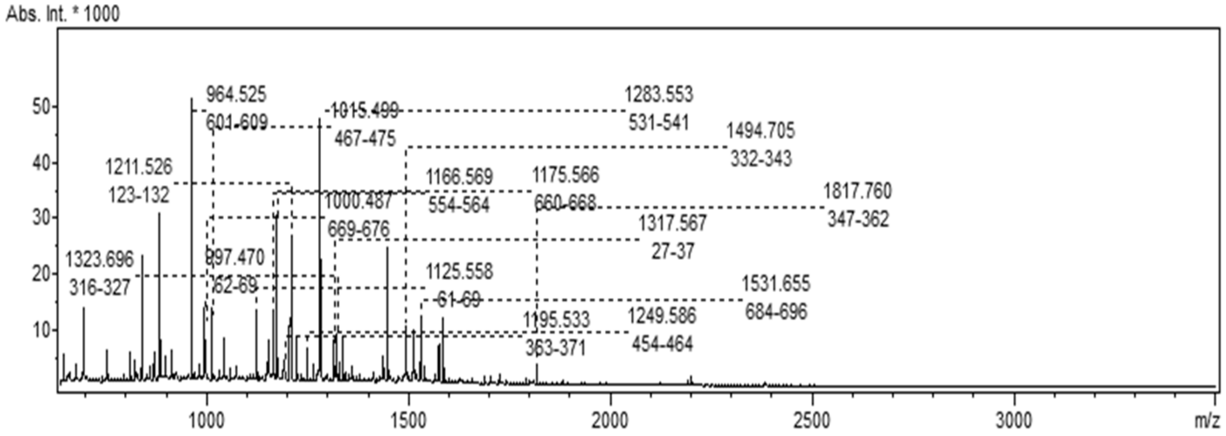

A representative MALDI-ToF PMF spectrum of GSN

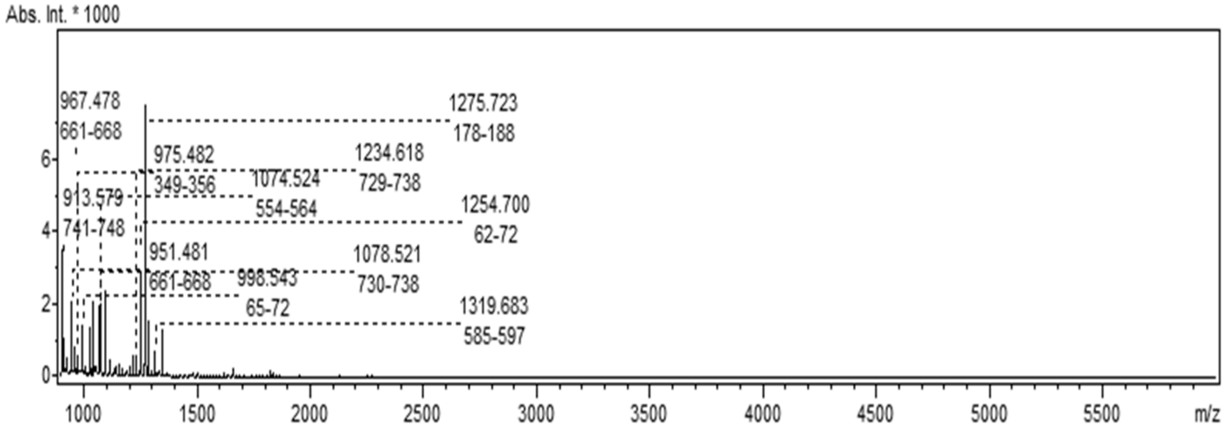

A representative MALDI-ToF PMF spectrum of DES, proteoform a

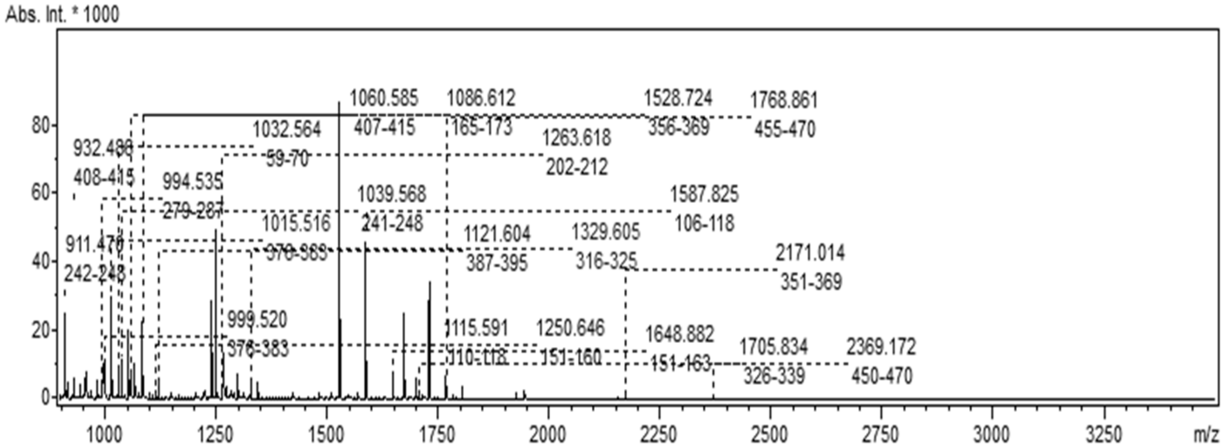

A representative MALDI-ToF PMF spectrum of DES, proteoform b

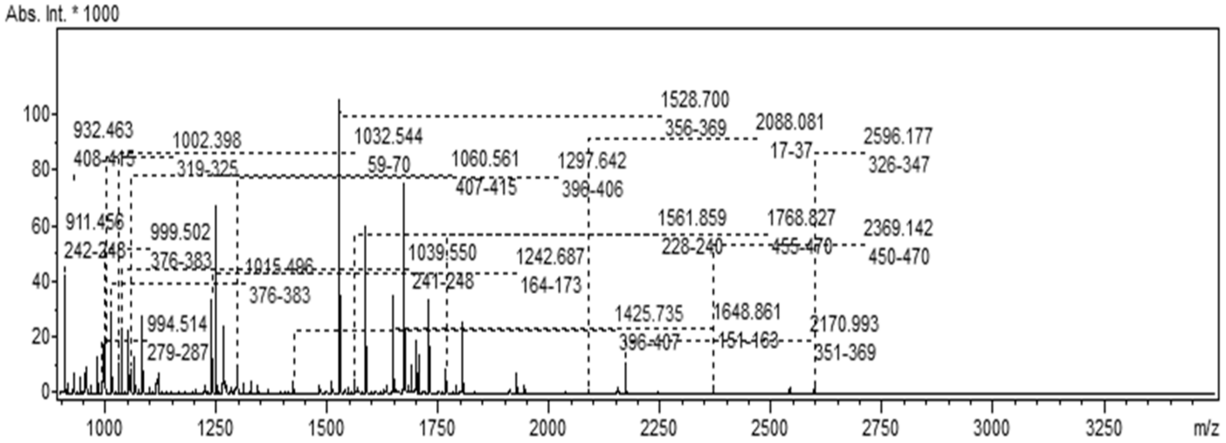

A representative MALDI-ToF PMF spectrum of MYH2

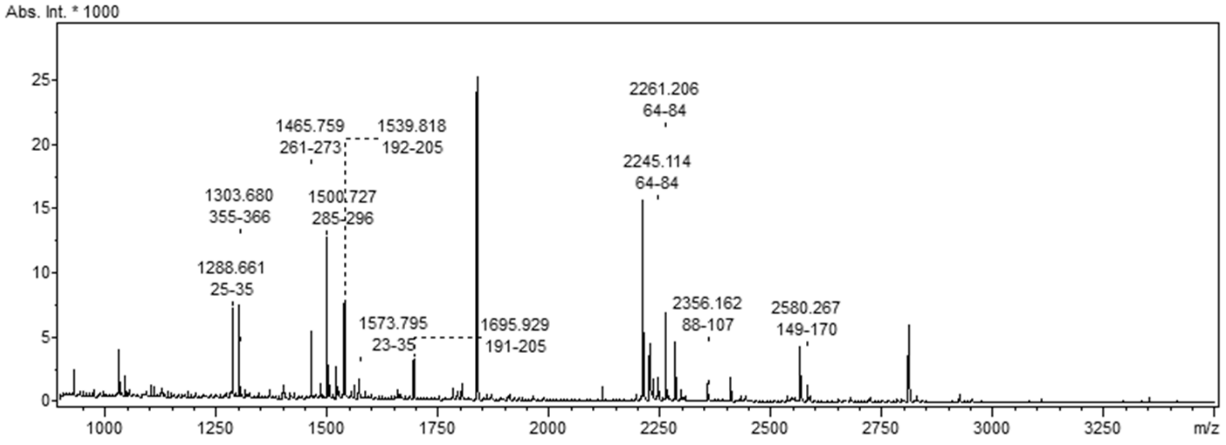

A representative MALDI-ToF PMF spectrum of ACTA1, proteoform a

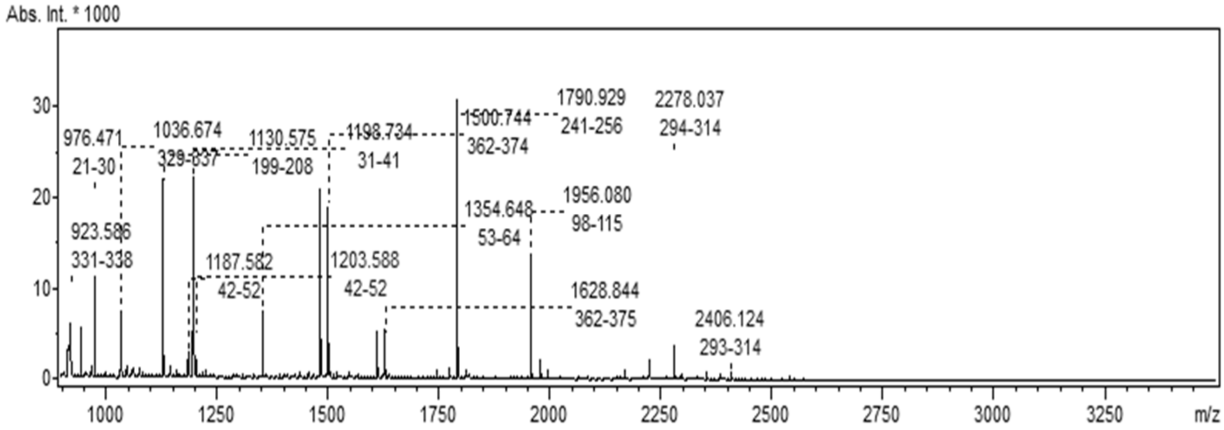

A representative MALDI-ToF PMF spectrum of ACTA1, proteoform b

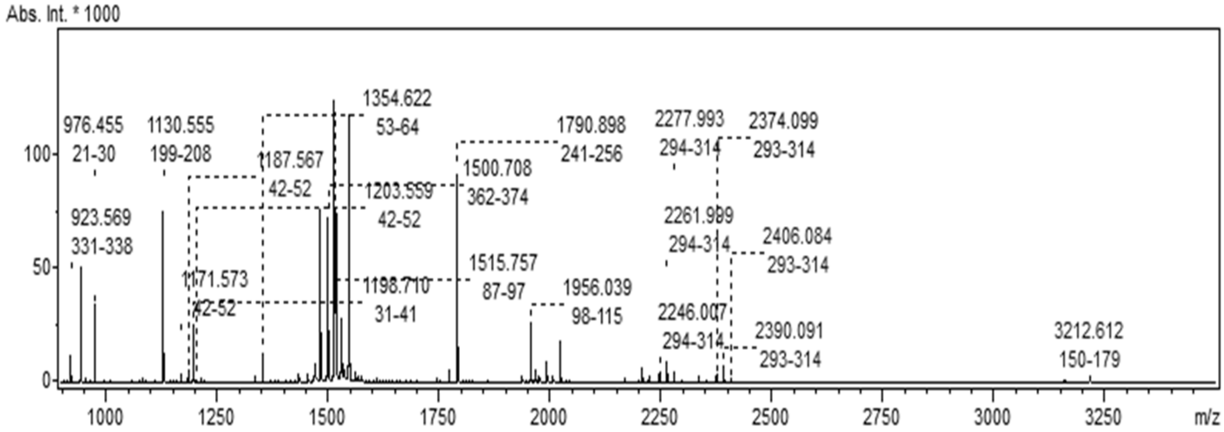

A representative MALDI-ToF PMF spectrum of ACTA1, proteoform c

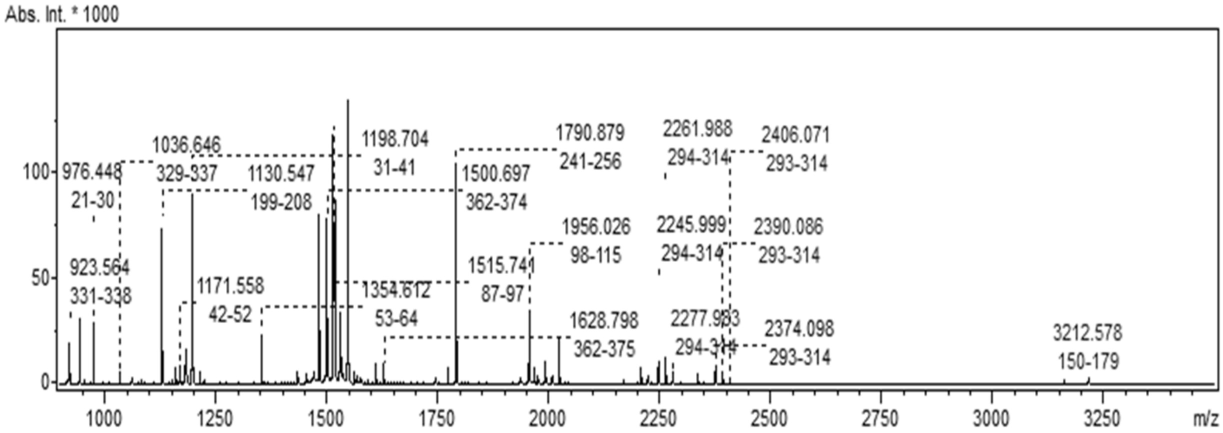

A representative MALDI-ToF PMF spectrum of ACTA1, proteoform d

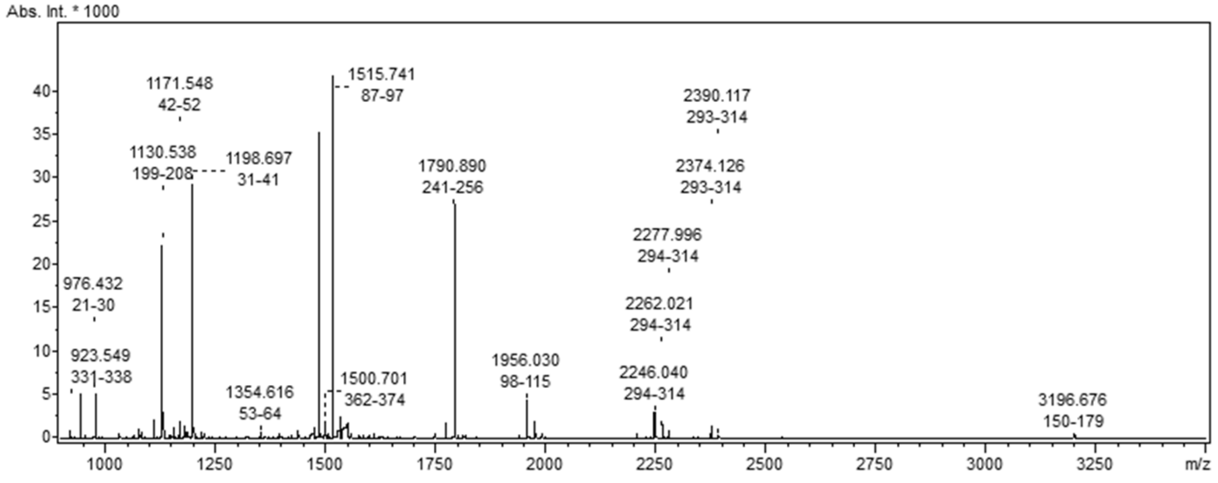

A representative MALDI-ToF PMF spectrum of ACTA1, proteoform e

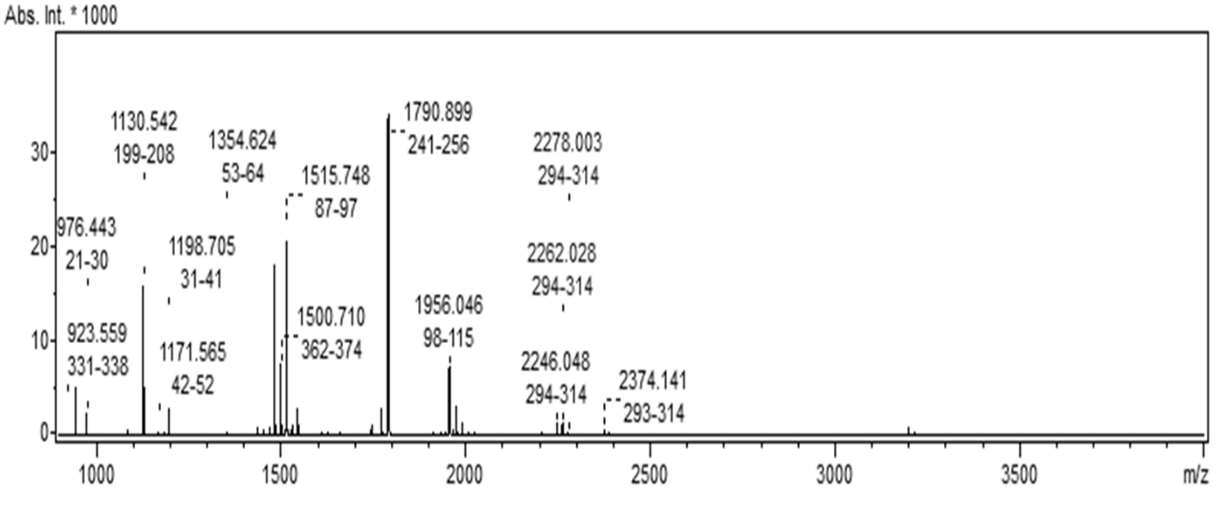

A representative MALDI-ToF PMF spectrum of FHL1

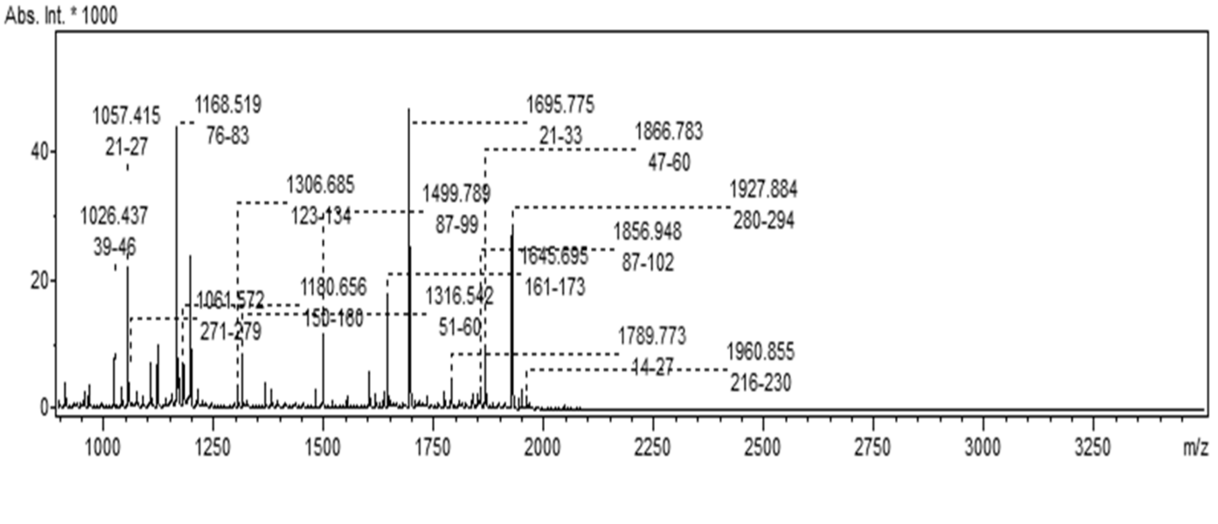

A representative MALDI-ToF PMF spectrum of LDB3

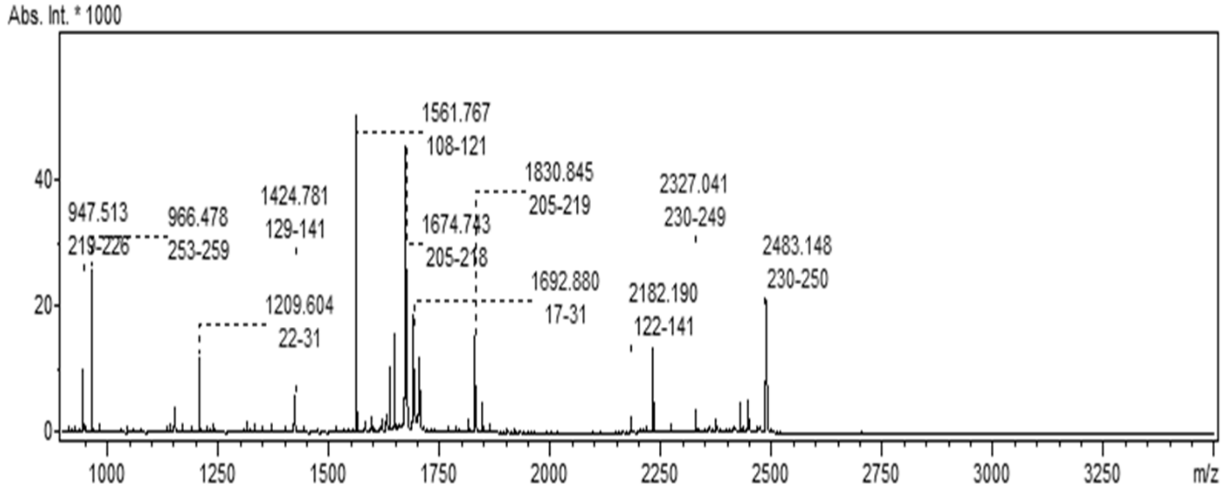

A representative MALDI-ToF PMF spectrum of MYLPF

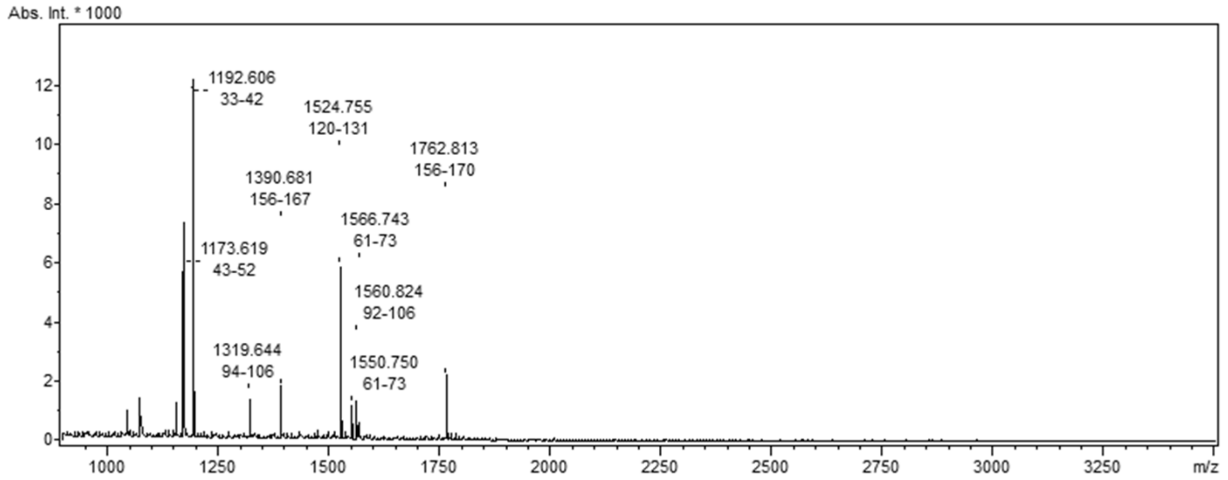

A representative MALDI-ToF PMF spectrum of ENO3

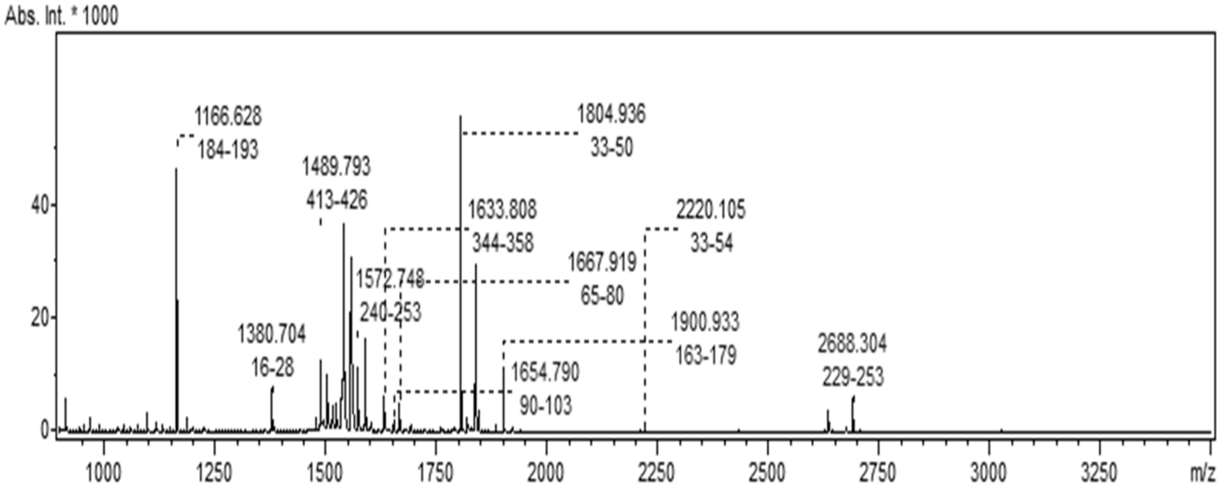

A representative MALDI-ToF PMF spectrum of PKM2

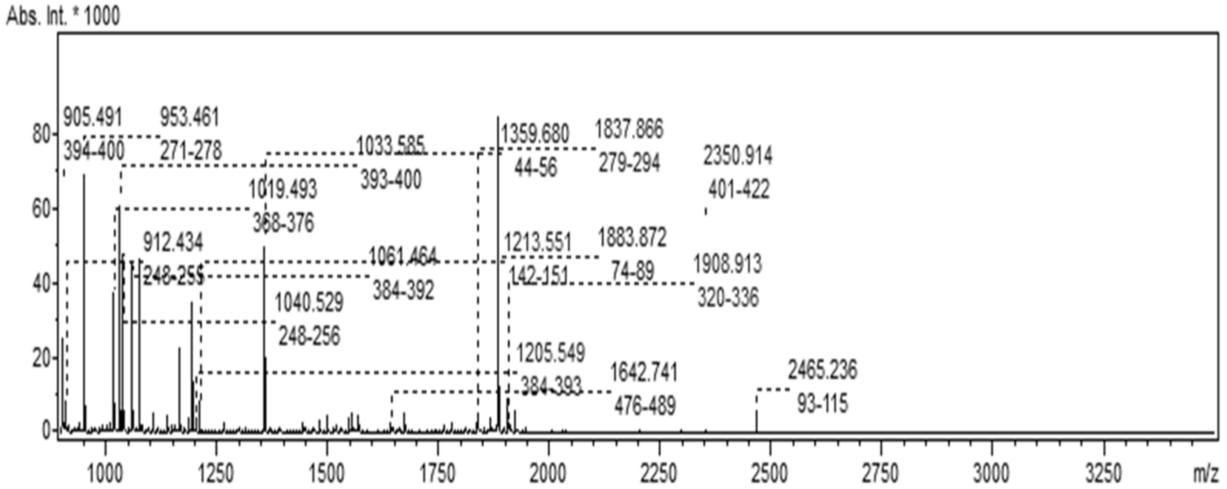

A representative MALDI-ToF PMF spectrum of GAPDH

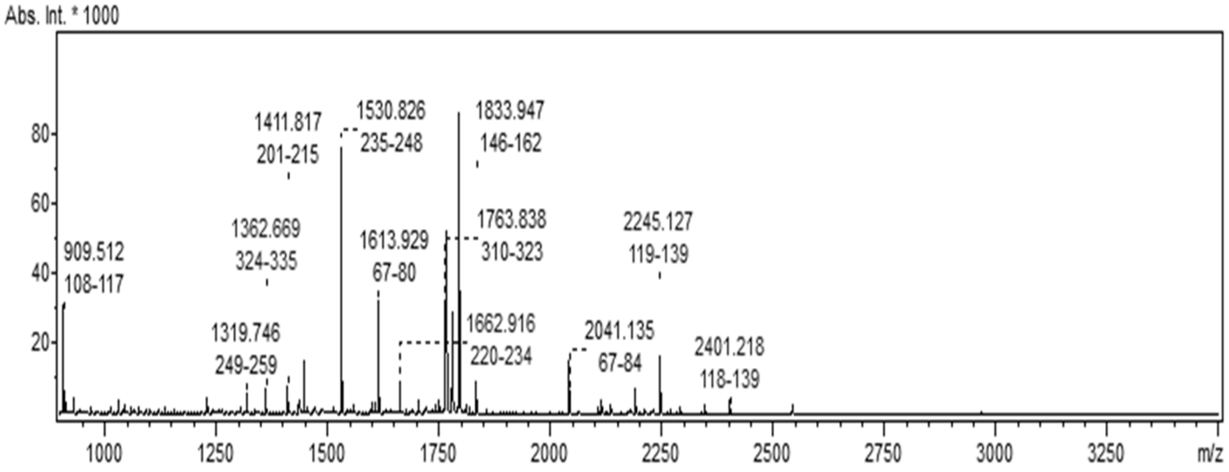

A representative MALDI-ToF PMF spectrum of IDH2

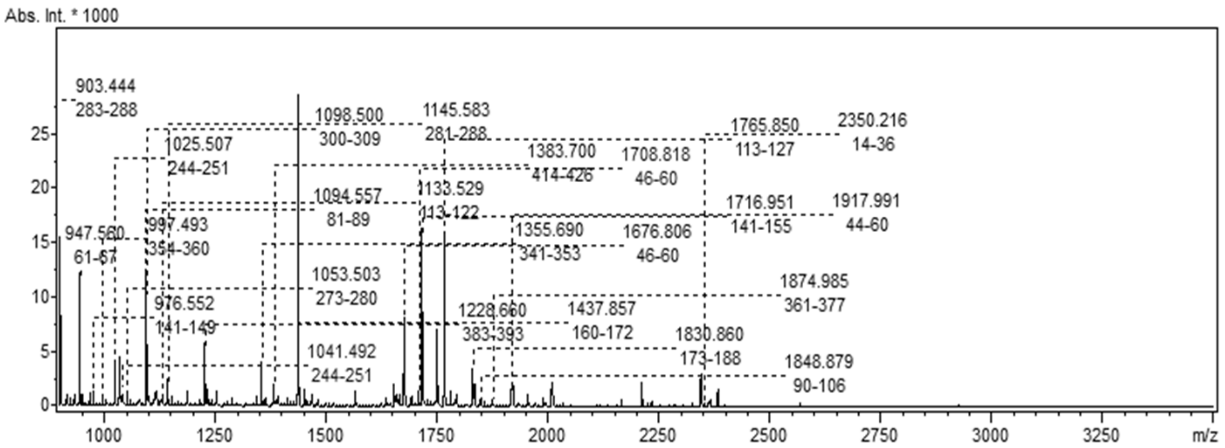

A representative MALDI-ToF PMF spectrum of CKMT2

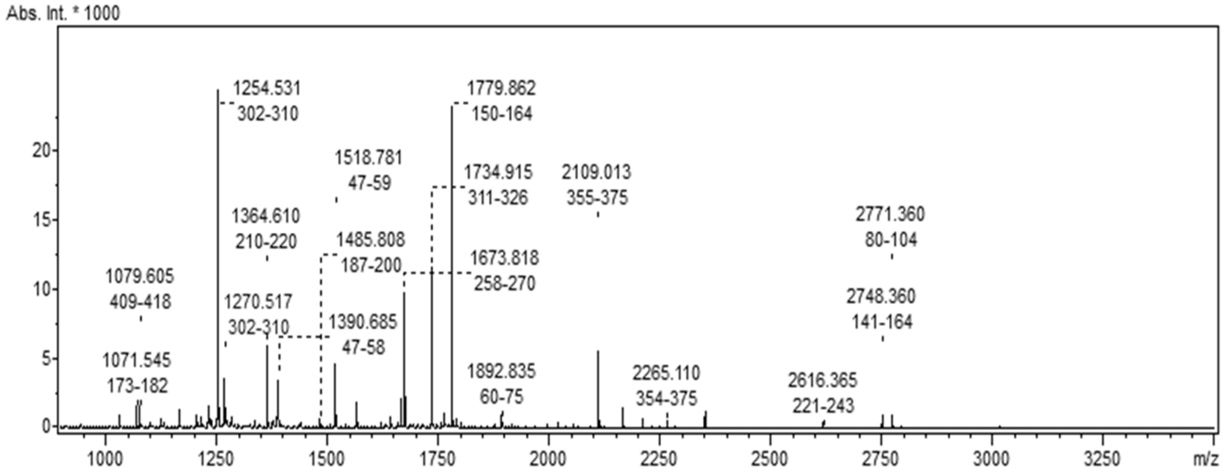

A representative MALDI-ToF PMF spectrum of UQCRC1

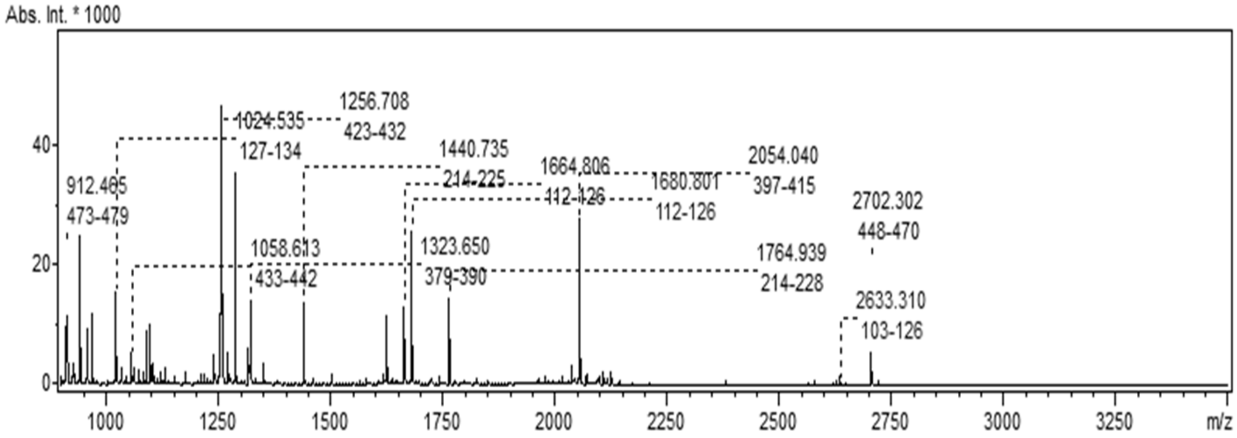

A representative MALDI-ToF PMF spectrum of TUFM

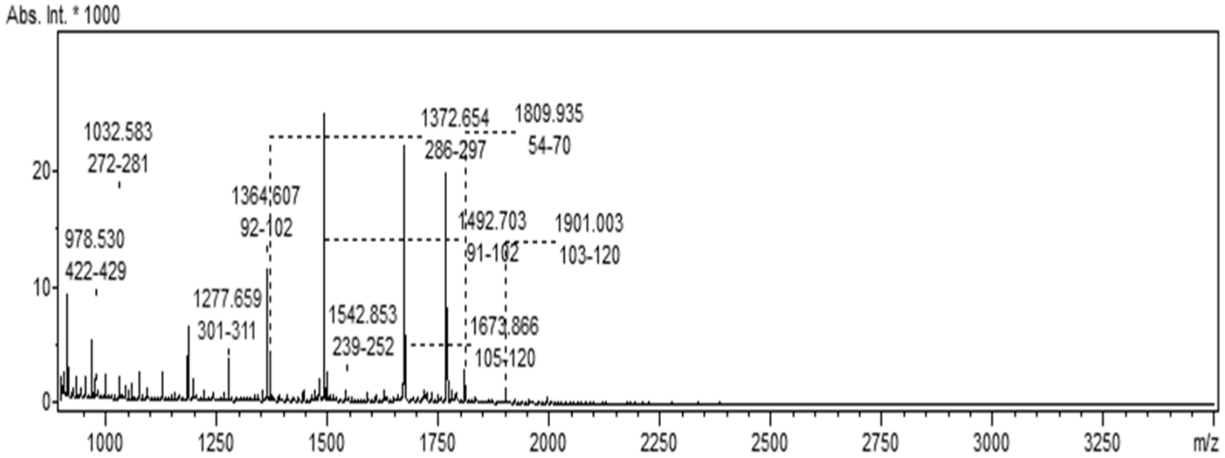

A representative MALDI-ToF PMF spectrum of EIF2S1

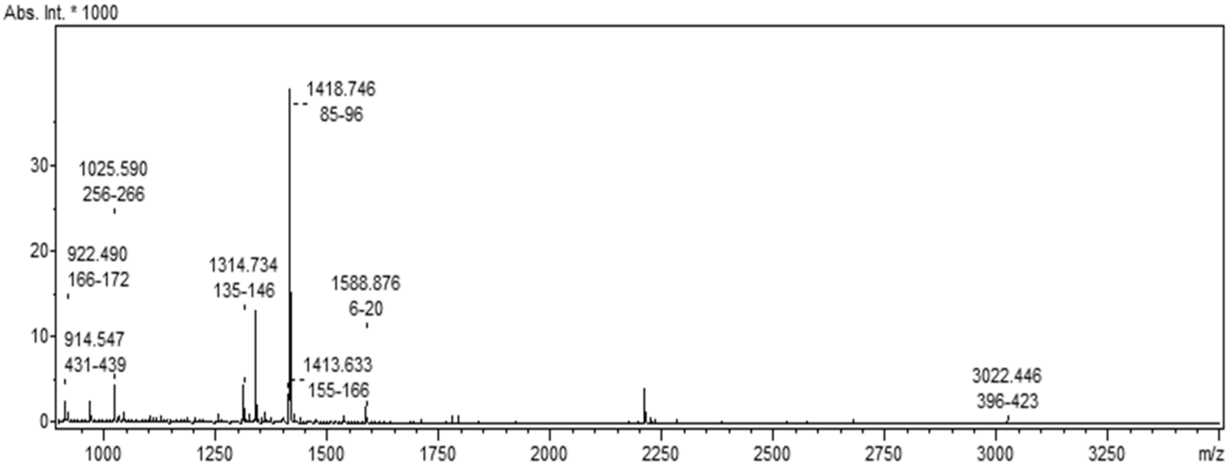

A representative MALDI-ToF PMF spectrum of HSP90AB1

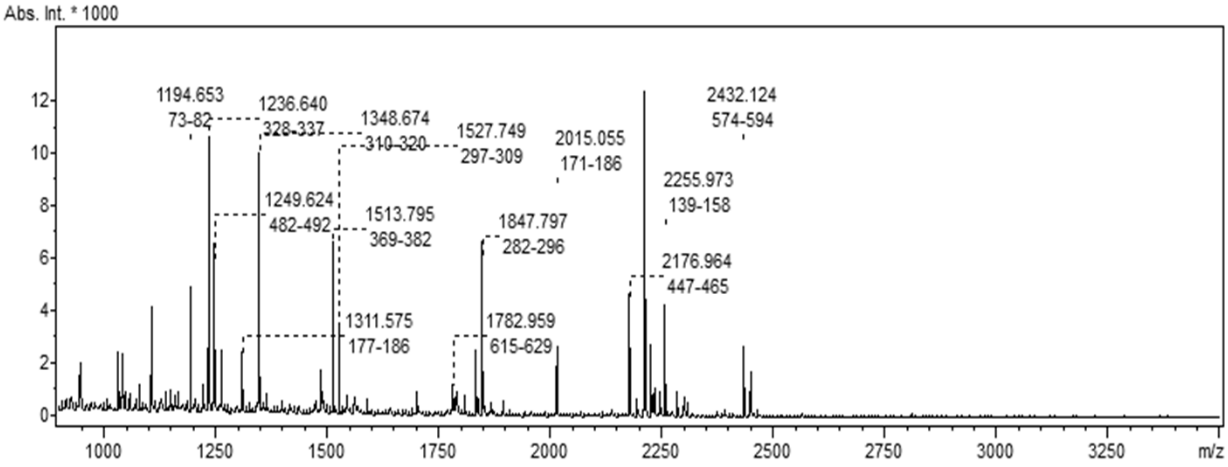

A representative MALDI-ToF PMF spectrum of PARK7

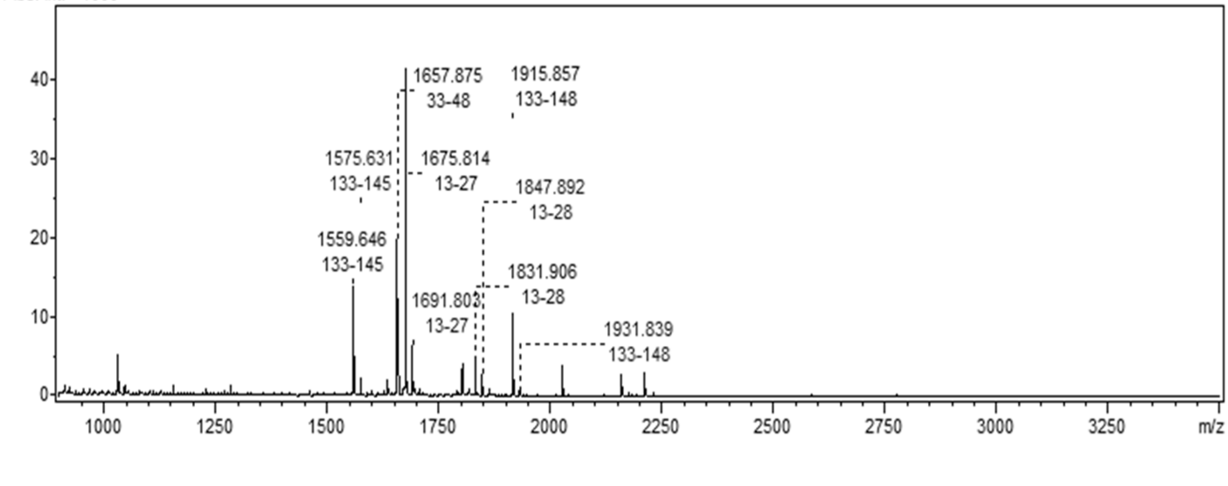

A representative MALDI-ToF PMF spectrum of TRIM72

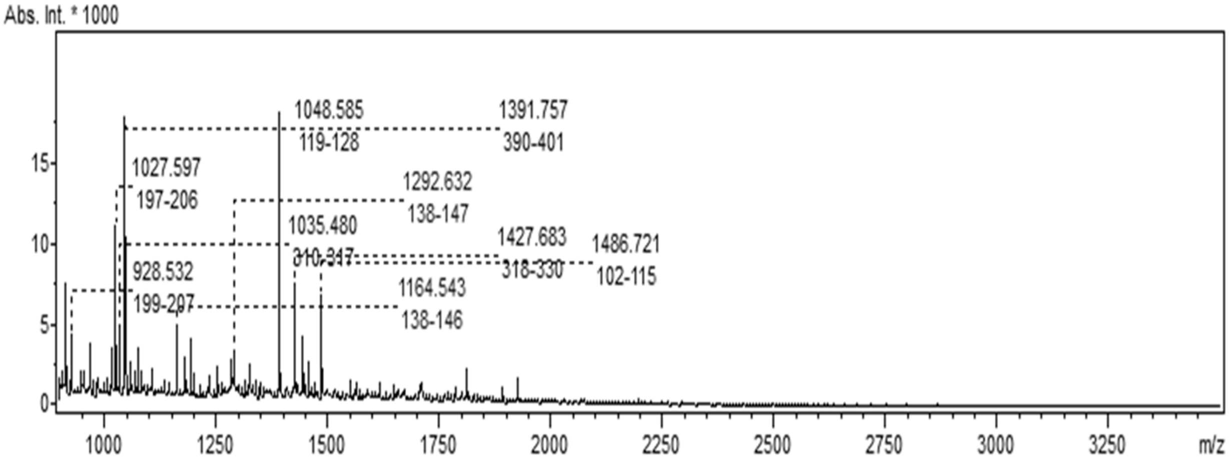

A representative MALDI-ToF PMF spectrum of HSPA5

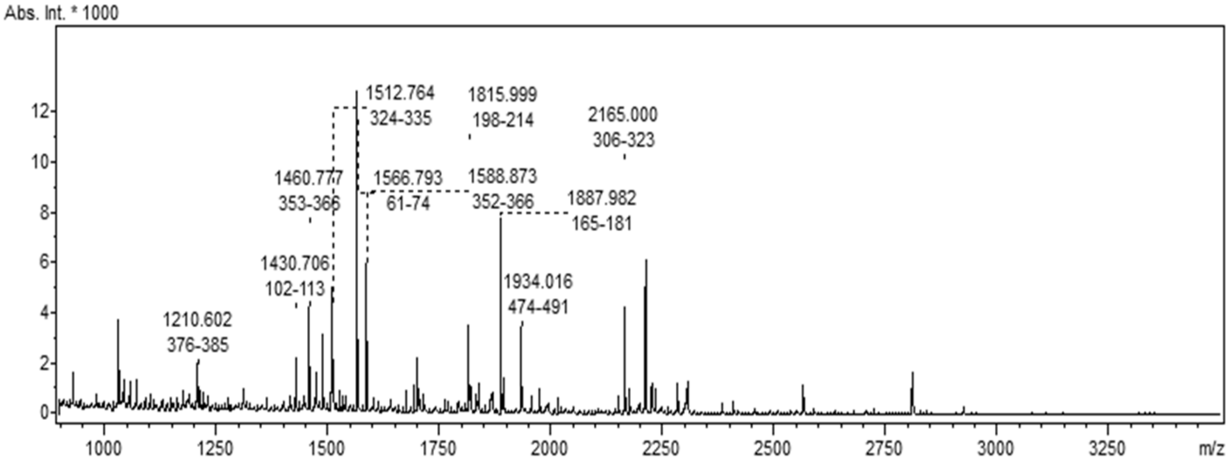

A representative MALDI-ToF PMF spectrum of HSPB1

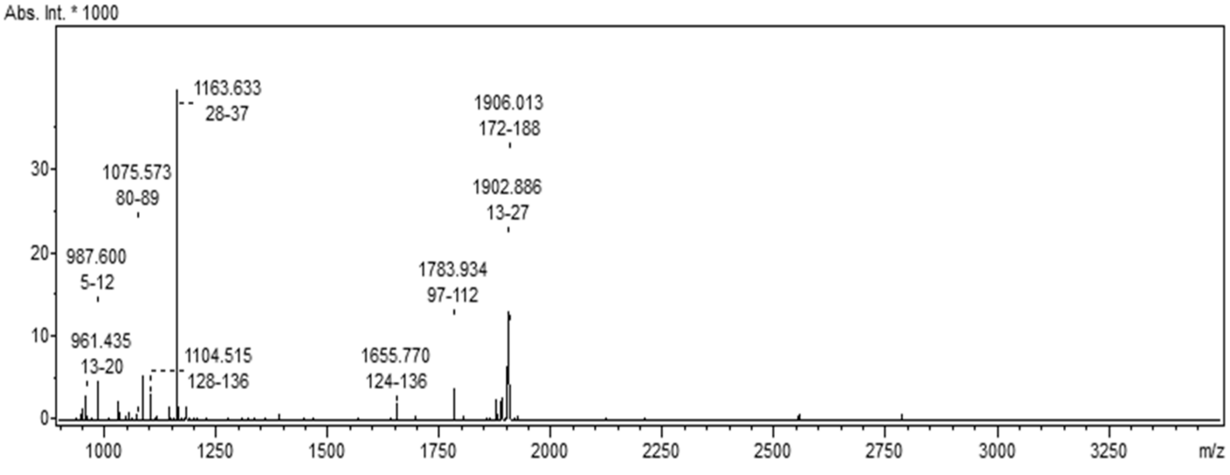

Supplement: Supplementary file 5 — Figure S5. List of annotated spectra for MALDI‐ToF identified proteins [file JCSM-11-547-s005.pdf]
