# Supplementary material for: Comparative proteomic analyses of Duchenne muscular dystrophy and Becker muscular dystrophy muscles: changes contributing to preserve muscle function in Becker muscular dystrophy patients
Source: J Cachexia Sarcopenia Muscle. 2020 Jan 28;11(2):547–63. doi: 10.1002/jcsm.12527 (PMC7113522; doi:10.1002/jcsm.12527)

**Figure S6.** Protein validation by immunoblotting. Representative histograms and immunoblot images of mitochondrial aconitase (ACO2), creatine kinase M-type (CKM), L-lactate dehydrogenase A chain (LDHA), succinate dehydrogenase flavoprotein subunit (SDHA), alpha-crystallin B chain (CRYAB) and vimentin (VIM) (n=3; mean ± S.D.; ANOVA and Tukey's test, $p<0.05$) in BMD (black bars) and DMD (gray bars) patients and healthy controls (white bars).

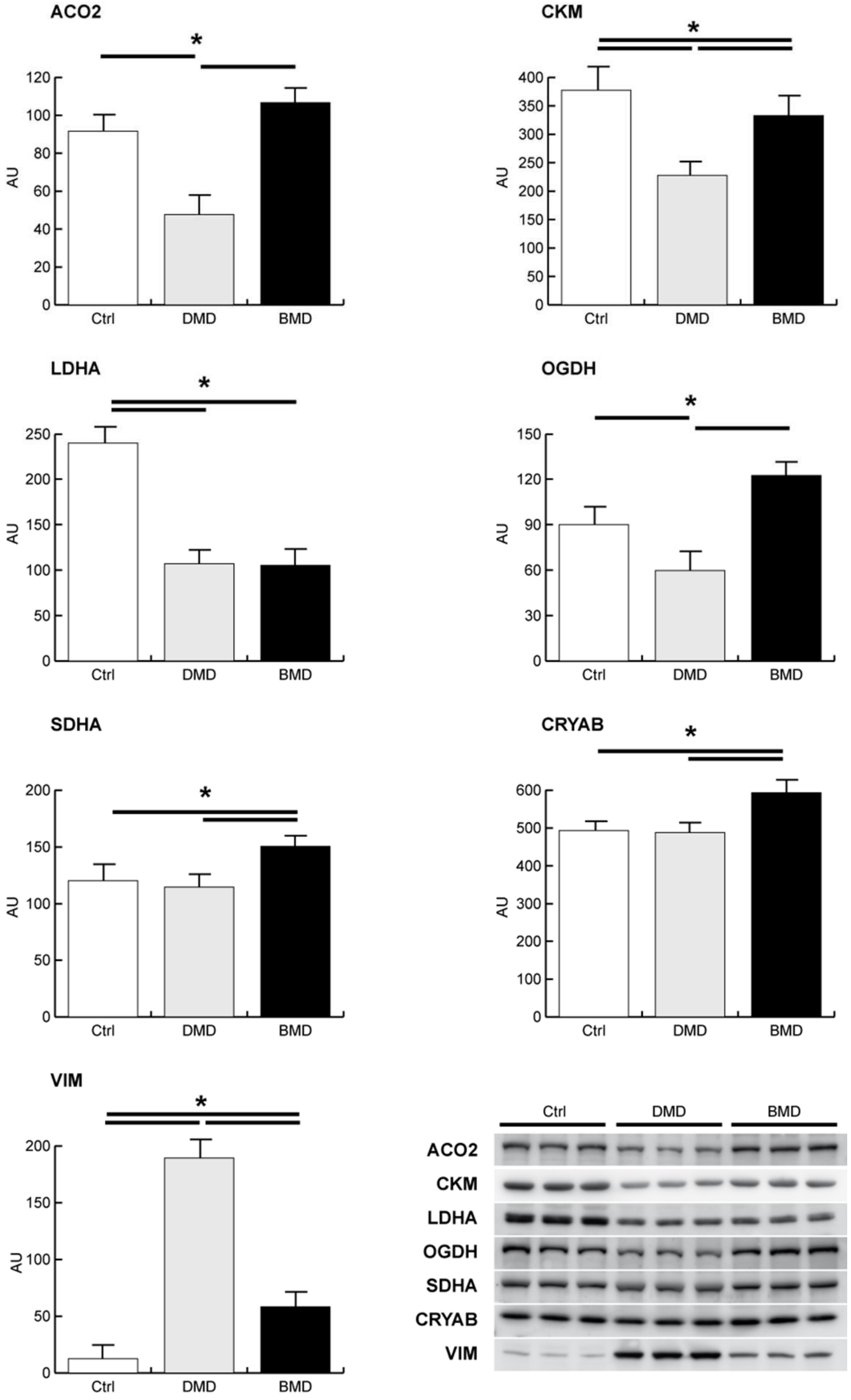

Supplement: Supplementary file 6 — Figure S6. Protein validation by immunoblotting. Representative histograms and immunoblot images of mitochondrial aconitase (ACO2), creatine kinase Mtype (CKM), L‐lactate dehydrogenase A chain (LDHA), succinate dehydrogenase flavoprotein subunit (SDHA), alpha‐crystallin B chain (CRYAB) and vimentin (VIM) (n = 3; mean ± S.D.; ANOVA and Tukey's test, P < 0.05) in BMD (black bars) and DMD (gray bars) patients and healthy controls (white bars). [file JCSM-11-547-s006.pdf]
